# Tracing neuroinflammation in neurodegeneration: insights from a scoping review on biofluid biomarkers

**DOI:** 10.1093/braincomms/fcag289

**Published:** 2026-08-06

**Authors:** Nadine A van de Zande, Charlotte C C Ruiter, Myrthe A Polman, Steffanie C M Brama, Raymund A C Roos, Philip H C Kremer, Susanne T de Bot

**Affiliations:** Department of Neurology, Leiden University Medical Center, Albinusdreef 2, 2300 RC Leiden, The Netherlands; Department of Neurology, Leiden University Medical Center, Albinusdreef 2, 2300 RC Leiden, The Netherlands; Department of Neurology, Medisch Spectrum Twente, Koningsplein 1, 7512 KZ Enschede, The Netherlands; Department of Neurology, Leiden University Medical Center, Albinusdreef 2, 2300 RC Leiden, The Netherlands; Department of Neurology, Leiden University Medical Center, Albinusdreef 2, 2300 RC Leiden, The Netherlands; Department of Neurology, Leiden University Medical Center, Albinusdreef 2, 2300 RC Leiden, The Netherlands; Centre for Human Drug Research (CHDR), Zernikedreef 8, 2333 CL Leiden, The Netherlands; Department of Neurology, Leiden University Medical Center, Albinusdreef 2, 2300 RC Leiden, The Netherlands

**Keywords:** neurodegenerative diseases, inflammation markers, clinical stratification, prognostic indicators

## Abstract

Neuroinflammation is increasingly recognized as a key pathological process in neurodegenerative disease and can be monitored using biofluid biomarkers. Objective biomarkers may aid diagnosis, prognosis and progression. We conducted a scoping review of neuroinflammation biomarkers across major neurodegenerative diseases covering the past 23 years, including Alzheimer’s disease, Parkinson’s disease, amyotrophic lateral sclerosis, frontotemporal dementia, Huntington’s disease, Lewy body dementia, multiple system atrophy and progressive supranuclear palsy. PubMed and Web of Science were systematically searched for observational studies from 2003 to 2025 reporting neuroinflammation biomarkers in adult human subjects. Included markers encompassed blood, cerebrospinal fluid, saliva and urine, providing possible complementary information. Original studies on non-neuroinflammatory mechanisms, cellular or post-mortem biomarkers, animal models, genetics and comparisons between diseases were excluded. Two reviewers independently screened articles; biomarkers reported in ≥3 independent cohorts per disease were analysed. A total of 388 studies were included, predominantly in Alzheimer’s disease/mild cognitive impairment (*n* = 214) and Parkinson’s disease (*n* = 92). Eight biomarkers were most frequently reported: IL-6, TNF-α, IL-1β, CRP/hs-CRP, IL-10, MCP-1, YKL-40 and neutrophil-to-lymphocyte ratio (NLR), measured in blood or cerebrospinal fluid (CSF) as indicators of inflammatory processes associated with neurodegeneration. Across biomarkers, the strength and scope of evidence varied. Most studies demonstrated higher biomarker levels in disease, with more advanced stages, greater clinical severity and faster progression. NLR showed the most consistent pattern across staging, severity and progression, but is currently under-represented across diseases. CSF YKL-40 generally increased with disease presence and advancement; IL-6 showed consistent increases in advanced stages and with severity, although significant results were limited; MCP-1, CRP and TNF-α were mostly linked to severity and progression; IL-1β and IL-10 remained largely inconsistent. Other markers, including GFAP, showed associations in Alzheimer’s disease but remain underexplored in other neurodegenerative diseases. Variability across studies, including differences in biofluid source, assay sensitivity, population characteristics and statistical approaches, limits interpretability and comparability. Although neuroinflammation is elevated in neurodegenerative diseases and generally intensifies as these diseases progress, potentially contributing to downstream pathology, the precise timing, role and predictive value of these biomarkers remain uncertain. A subset of markers, including NLR, YKL-40 and GFAP, shows relatively consistent associations and may warrant further investigation across diseases. In clinical practice, neuroinflammation biomarkers could serve as complementary tools to capture inflammatory processes related to disease heterogeneity and progression. Future longitudinal studies tracking pre-symptomatic and early-stage individuals, with standardized approaches, are needed to define temporal dynamics and explore their utility for monitoring disease progression and therapeutic response.

## Teaser

Van de Zande *et al*. conducted a scoping review analysing 388 studies mapping the current landscape of biofluid neuroinflammation biomarkers across multiple neurodegenerative diseases. The review identified associations between biomarker levels and disease presence, severity and progression, and evaluated their consistency, overlap, and potential clinical value.

## Graphical Abstract

**Graphical Abstract:**
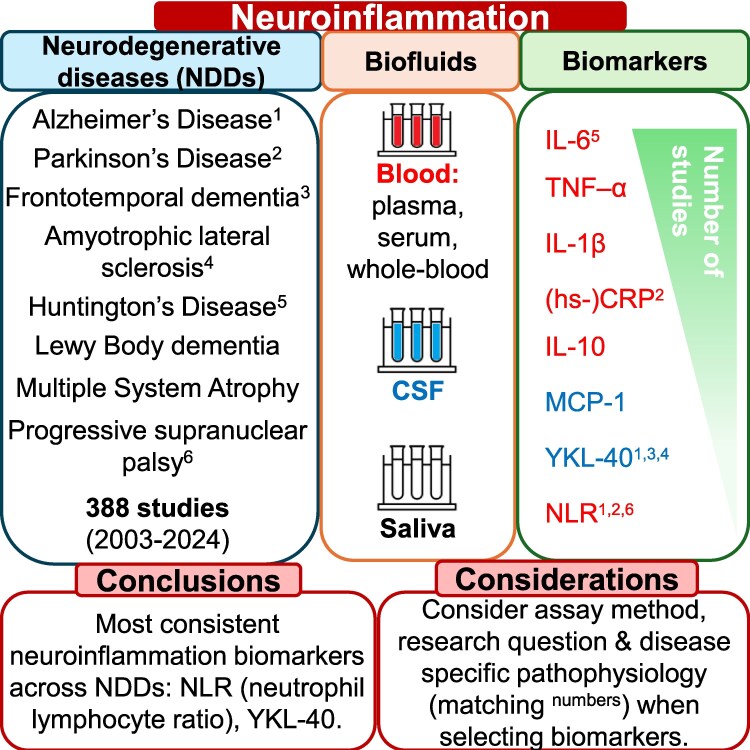

## Introduction

Identifying reliable biomarkers is a critical goal in biomedical research, aiming to improve diagnosis, predict prognosis, monitor disease progression and evaluate treatment response in neurodegenerative diseases. Biomarkers are defined as characteristics that are objectively measured and evaluated as indicators of normal biological processes, pathogenic processes or pharmacologic responses to a therapeutic intervention.^1^ Multiple pathophysiological mechanisms contribute to neurodegeneration, resulting in a diversity of biomarkers, many of which overlap across different neurodegenerative disorders.^2,3^ Among these mechanisms, neuroinflammation has gained increasing attention as a key contributor to disease progression.^4^

Neuroinflammation is characterized by an immune response within the central nervous system (CNS) triggered by diverse insults, including trauma, ischaemia, genetic mutations and protein misfolding disorders. In response to neuronal injury, inflammatory mediators activate microglia and astrocytes, leading to chemokine production and recruitment of peripheral immune cells.^5^ Microglia, the resident immune cells of the CNS, play a crucial role in this process and are found to be significantly involved in Alzheimer’s disease, Parkinson’s disease, progressive supranuclear palsy and other neurodegenerative conditions.^6^ While microglial responses have historically been described as ‘activation’, current evidence suggests a more complex spectrum of microglial states during neurodegeneration.^7^ These responses are associated with the release of pro-inflammatory cytokines, oxidative stress and complement system activation, which can contribute to neuronal damage and synaptic dysfunction.^8,9^

Although neuroinflammation initially serves a protective role by containing damage and clearing cellular debris, persistent or dysregulated inflammatory responses lead to chronic neuroinflammation, which exacerbates neurodegeneration.^10^ Neuroinflammation contributes to disease progression in Alzheimer’s disease, Parkinson’s disease and amyotrophic lateral sclerosis.^11^ In most neurodegenerative diseases, protein misfolding and aggregation induce neuronal toxicity, which in turn triggers neuroinflammation and neurodegeneration.^12,13^ Although the exact causal relationship between neuroinflammation and neurodegeneration remains unclear, an impaired homeostasis of inflammatory molecules may accelerate the disease onset and progression. Identifying these inflammatory markers in the early or pre-symptomatic stages of a disease could improve early diagnosis of neurodegenerative disorders. Beyond its role in disease onset and progression, neuroinflammation is a key target for developing preventive and disease-modifying therapies aimed at slowing or halting neurodegeneration.^14^

This scoping review examines neuroinflammation biomarkers identified in neurodegenerative diseases over the past 23 years, including Alzheimer’s disease (AD), Parkinson’s disease (PD), amyotrophic lateral sclerosis (ALS), frontotemporal dementia (FTD), dementia Lewy body (DLB), Huntington’s disease (HD), multiple system atrophy (MSA) and progressive supranuclear palsy (PSP). Studies on neuroinflammation biomarkers are often disease-specific, limiting cross-disease insights. Therefore, the primary aim of this study is to identify potential biomarker overlap across different neurodegenerative diseases. Given the partial shared pathophysiological mechanisms of neurodegenerative diseases, a secondary aim is to identify neuroinflammation biomarkers that are well established in one disease and may warrant further investigation in others. As our aim is to identify objective biomarkers that reflect disease presence, stage, severity and progression, we focus on biomarkers that change with the natural course of the disease rather than those primarily used for treatment monitoring, where changes may reflect treatment effects rather than underlying pathology.

## Materials and methods

The structure of the search strategy for this scoping review was based on the Preferred Reporting Items for Systematic Reviews and Meta-Analyses (PRISMA) guidelines.^15^ We used a scoping review approach, because our aim was to comprehensively map the extent, characteristics, and variability of evidence on neuroinflammation biomarkers in neurodegenerative diseases. For the purpose of this review, biomarkers were included if the original studies reported them as indicators of neuroinflammation. We note that some of these biomarkers, particularly when measured in blood, may also reflect systemic inflammation.

The protocol for this scoping review was not preregistered.

### Search strategy

With the support from a medical librarian, N.A.v.d.Z. performed a multi-database literature search in PubMed and Web of Science to identify relevant full-text articles in English published between 2003 and 2025 (see Supplementary Materials: ‘Search Strategy’). Search terms included disease-specific terms (i.e. AD, PD, ALS, HD, MSA, FTD and PSP) and biomarker-related terms such as ‘inflammation’, ‘disease progression’, ‘blood’, CSF’, ‘MRI’ and ‘PET’. The results of the literature search were imported and managed into Covidence, a web-based collaboration platform that streamlines the production of systematic and other literature reviews and ensures transparent and reproducible screening and data management.

### Selection of articles

Articles were selected based on the inclusion of the specified neurodegenerative diseases, including Alzheimer’s disease, Parkinson’s disease, amyotrophic lateral sclerosis, frontotemporal dementia, dementia Lewy body, Huntington’s disease, multiple system atrophy and progressive supranuclear palsy. We acknowledge that DLB was not included as a specific search term; however, it was part of the inclusion criteria and studies on dementia Lewy body were included to ensure coverage of this disease. Eligible studies were observational and investigated biomarkers related to disease presence, severity or progression in living adult humans. Included biomarkers comprised wet biomarkers and imaging markers. If studies shared the same cohort, they were still considered separate studies if the sample size differed, as this may indicate that data were collected at different time points or that different participants were included.

Exclusion criteria included studies focusing on other conditions, interventional trials, and studies comparing biomarkers between neurodegenerative diseases but not with controls. To maintain a focused and concise overview of biomarkers directly reflecting neuroinflammatory processes, studies investigating biomarkers primarily linked to other biological pathways (e.g. oxidative stress, hormonal regulation, infections or gut microbiota) were excluded, even though these may influence neuroinflammation. To improve comparability, studies examining cellular or subcellular biomarkers (e.g. monocyte-specific levels, specific immune cell populations, microvesicles), post-mortem tissue, *ex vivo* models, lab animal data, genetic or transcriptomic analyses (DNA-, RNA- or gene expression based) or top-down proteomics were excluded, as well as studies that only reported correlations between neuroinflammation biomarkers, without relating them to clinical or disease outcomes. Only original research articles published in English were included, excluding reviews, meta-analyses, letters to the editor, methodological papers or pathophysiological descriptions.

All titles and abstracts were reviewed, and full texts of the selected articles were examined independently by N.A.v.d.Z. and M.A.P., who also determined study eligibility. Any discrepancies were resolved through mutual consensus and, when necessary, consultation with S.T.d.B. For further details, refer to Supplementary Table 1: ‘Inclusion and Exclusion Criteria’.

### Data analysis

Data were extracted by three authors (N.A.v.d.Z., S.C.M.B. and C.C.C.R.), each extracting separate articles using a pre-defined extraction template and consulting each other as needed to ensure consistency and to resolve uncertainties regarding study characteristics or reported outcomes. The selected articles were evaluated with respect to study design, population characteristics, biomarkers assessed and their reported outcomes. To enable conclusions to be drawn that reflect the majority of available evidence, for each disease a minimum of three independent study cohorts including both disease group and controls were required in order for a biomarker to be included in the further analysis. The robustness of findings was assessed by N.A.v.d.Z., under supervision of S.T.d.B., by comparing the proportion of significant versus non-significant results and evaluating the consistency of direction across studies within the same medium for each biomarker. We evaluated the biomarkers’ performance in differentiating disease from controls, across disease stages, and in relation to clinical characteristics and longitudinal progression.

## Results

### Study selection

A total of 8215 records were identified through database searches (PubMed: 4271; Web of Science: 4235), with overlapping records removed during screening in Covidence. The first search was done on 23 August 2022, on Pubmed, for articles from January 2003 up until that point, followed by a search on Web of Science on 9 January 2023, for articles from January 2003 up until the search date, to capture studies from the past 20 years. The search was extended to include the years 2023 and 2024 (search on 10 January 2025).

After automatic removal in Web of Science of 109 studies in non-English, 273 studies with different study designs like reviews and editorials, and automatic removal of 1276 duplicates in the Covidence platform, 6848 records remained. Title and abstract screening excluded 6083 records, and 231 full-text articles were excluded after eligibility assessment, resulting in 452 articles for data extraction. Only three MRI studies were found, even though both MRI and magnetic resonance spectroscopy were included in the search strategy. There were 29 original PET studies (18 Alzheimer’s disease, 1 amyotrophic lateral sclerosis, 1 dementia Lewy body, 2 frontotemporal dementia, 2 Huntington’s disease, 5 Parkinson’s disease). Given the predominance of wet biomarker studies compared with neuroimaging studies, and to maintain a focused, interpretable and readable analysis, PET and MRI studies were excluded from further analysis prior to data extraction. Including all study types would have disproportionately weighted the review towards biofluid biomarkers and reduced both clarity and comparability of the findings.

**Figure 1:**
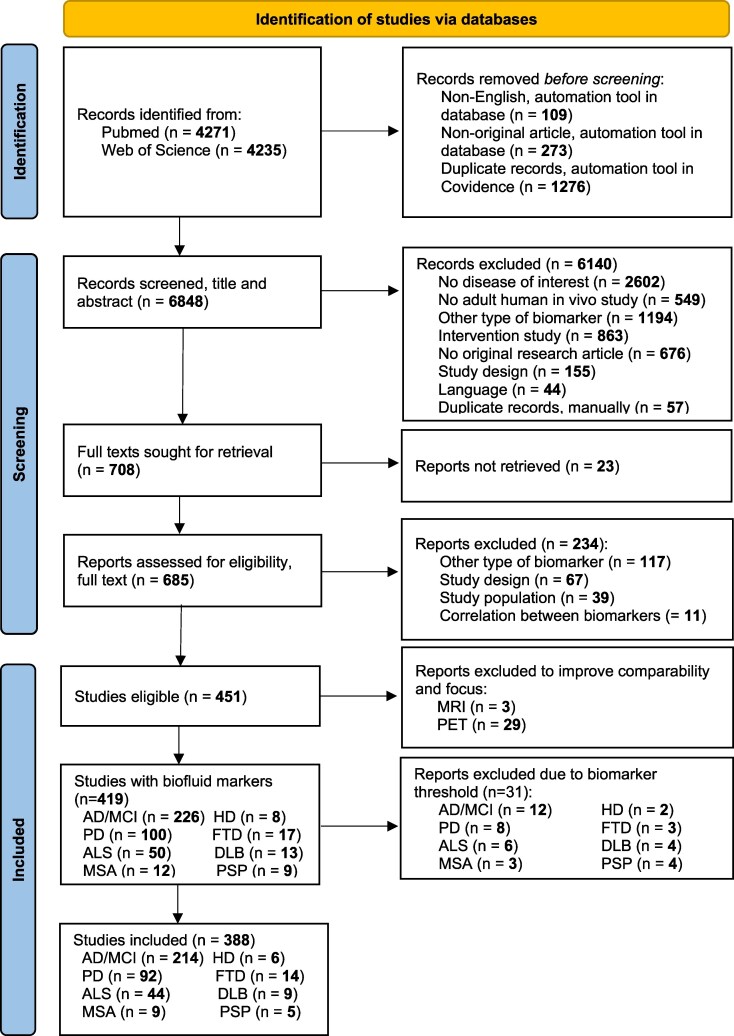
**PRISMA flow diagram. Adapted from the PRISMA 2020 statement.** PRISMA flow diagram summarising the study selection process for this scoping review. The diagram illustrates the identification, screening, eligibility assessment, and inclusion of studies. It shows the number of records identified through database searches, duplicates removed, records screened by title and abstract, and full-text articles assessed for eligibility, including reasons for exclusion. The final included studies are shown and categorised by disease. AD, Alzheimer’s disease; ALS, amyotrophic lateral sclerosis; DLB, dementia with Lewy bodies; FTD, frontotemporal dementia; HD, Huntington’s disease; MCI, mild cognitive impairment; MRI, magnetic resonance imaging; MSA, multiple system atrophy; *n,* number; PET, positron emission tomography; PD, Parkinson’s disease; PSP, progressive supranuclear palsy.

As some articles cover multiple diseases, the total number of articles per disease may exceed the overall number of articles reviewed. A total of 419 articles studying neuroinflammation biofluid markers were found. From these articles, biomarkers were selected that were measured in ≥3 different research cohorts, resulting in the exclusion of a large number of markers (>300) that were only studied in one or two cohorts, as well as 31 unique articles (presented in the Supplementary Materials). This resulted in the inclusion of a total of 388 studies, distributed as follows: Alzheimer’s disease/mild cognitive impairment (Alzheimer’s disease/MCI; *n* = 214), Parkinson’s disease (*n* = 92), amyotrophic lateral sclerosis (*n* = 44), Huntington’s disease (*n* = 6), frontotemporal dementia (*n* = 14), multiple system atrophy (*n* = 9), dementia Lewy body (*n* = 9) and progressive supranuclear palsy (*n* = 5). The PRISMA flow diagram of the selection process is illustrated in Fig. 1.

### Results of the analyses

For the purpose of calculating the total number of biomarkers investigated, white blood cell-derived measures (including ratios and cell counts) were counted as one biomarker. In Alzheimer’s disease/MCI, 64 biomarkers were assessed in ≥3 studies. Table 1 summarizes the most frequently studied biomarkers, while a complete list of the biomarkers measured in Alzheimer’s disease is listed in Supplementary Table 2. For Alzheimer’s disease, most study populations were defined based on NINCDS-ARDRA,^16^ DSM-IV-TR^17^ and/or NIA-AA^18^ criteria. MCI was generally defined based on clinical diagnostic criteria.^19,20^ Nineteen studies applied the ATN (Amyloid/Tau/Neurodegeneration) framework for group classification. As these classifications are not directly comparable with the combined diagnostic criteria in other studies, they were not included in the tables; however, their findings are described in the main text. In Parkinson’s disease, 40 biomarkers were measured in ≥3 studies. The most investigated biomarkers are presented in Table 2, and a complete list of the biomarkers is reported in Supplementary Table 3. In frontotemporal dementia, 20 biomarkers were measured in ≥3 studies; the most frequently studied biomarkers are shown in Table 3 and a complete list of the biomarkers in Supplementary Table 4. For clarity, different forms of frontotemporal dementia (e.g. phenotypic or genetic variants) were combined, with subtypes specified only when results applied to a specific subtype. In amyotrophic lateral sclerosis, 19 biomarkers were measured in ≥3 studies, with the most frequently investigated biomarkers presented in Table 4 and a complete list of the biomarkers in Supplementary Table 5. In dementia Lewy body, 20 biomarkers met this criterion, with the most frequently studied biomarkers presented in Table 5A and a complete list of the biomarkers in Supplementary Table 6. In multiple system atrophy, all six identified biomarkers are shown in Table 5B; in Huntington’s disease, all three in Table 5C; and in progressive supranuclear palsy, the single identified biomarker in Table 5D. This structural grouping reflects practical considerations and should not be interpreted as suggesting comparability of underlying inflammatory mechanisms across these diseases.

**Table 1:** Alzheimer’s disease results in different studies

Biomarker (number of studies)	Number of studies by biofluid type					
	Ns. Blood	Ns. CSF	↑ Blood	↓ Blood	↑ CSF	↓ CSF
**IL-6 (83):** **blood (67), CSF (21)**						
Below detection limit	**1** ^21^					
AD versus control	**26** ^22-47a^	**8** ^25,38,48-53^	**12** ^37b,54-64^	**1** ^65^	**2** ^66,67^	**1** ^65^
AD versus MCI	**18** ^29,31-46,68^	**7** ^38,48-53^	**5** ^22,61,68-70^			
MCI versus control	**16** ^31-46^	**9** ^38,48-53,71,72^	**5** ^29,63,68,73,74^	**3** ^65,71,75a^		**1** ^65^
**TNF-α (63):** **blood (53), CSF (14)**						
Below detection limit	**1** ^21^	**5** ^30,49,51,52,71^				
AD versus control	**20** ^23,24,26-29,31-35,39,40,42,44,46,47,60,61,76^	**4** ^25,50,65,77^	**11** ^22,54,55,62-65,68,78-80^	**3** ^25,81,82,a^	**2** ^66,67^	
AD versus MCI	**13** ^22,29,31,32,34,35,39,40,42,44,46,61,69^	**2** ^50,65^	**4** ^33,70,79,80^			
MCI versus control	**14** ^22,29,31,32,34,35,39,40,42,44,46,61,74,79^	**2** ^50,65^	**7** ^63,65,68,71,73,80,83^	**1** ^84c^		
**IL-1β (50):** **blood (43), CSF (11)**						
Below detection limit	**3** ^21,34,69^	**5** ^30,49,51,52,71^				
AD versus control	**15** ^23-27,29,31-33,35,37,46,61,82a,85a^	**3** ^25,65,66^	**12** ^28,37b,38,55,60,63-65,68,86-88^		**2** ^38,67^	
AD versus MCI	**8** ^29,31,32,38,46,61,68,85^	**2** ^38,65^	**4** ^37b,70,86,88^	**2** ^33,35^		
MCI versus control	**7** ^29,31,32,46,61,71,85^	**1** ^65^	**11** ^35,38,63,65,68,71,73,83,84c,87,88^	**1** ^75a^	**1** ^38^	
**MCP-1 (49):** **blood (34), CSF (23)**						
AD versus control	**10** ^31,32,34,41,46,68,81,89-91^	**12** ^30,49,51-53,66,89,90,92-95a^	**4** ^23,55,60,96^	**5** ^27,65,82a,97,98^	**2** ^99,100^	**1** ^65^
AD versus MCI	**9** ^31,32,34,41,68,69,81,90,91^	**7** ^49,51-53,90,94,99^	**1** ^96,a^	**2** ^31,46^		
MCI versus control	**7** ^32,34,41,68,81,91,101a^	**6** ^49,51,53,71,90,94^	**4** ^31,46,71,90^	**3** ^65,71,75^	**2** ^72,99^	**1** ^65^
**IL-8 (43):** **blood (33), CSF (15)**						
AD versus control	**10** ^27,31-33,40,44,46,60,65,82a^	**9** ^25,30,49,51-53,65,95a,100^	**4** ^23,43,68,102^	**3** ^25,55,81^	**1** ^66^	
AD versus MCI	**9** ^31-33,40,44,46,65,68,81^	**5** ^49,51-53,65^				
MCI versus control	**8** ^31-33,40,46,65,68,71^	**5** ^49,51-53,65^	**2** ^43,71^	**2** ^44,81^	**2** ^71,72^	
**IL-10 (42):** **blood (36), CSF (11)**						
Below detection limit	**1** ^69^	**4** ^49,51,52,71^				
AD versus control	**17** ^25-27,31-35,38-40,44,46,47,65,81,82,a^	**3** ^38,50,65^	**2** ^28,55^	**6** ^27,30,33,35,38,68^	**2** ^25,66^	
AD versus MCI	**11** ^31,32,34,35,38-40,46,65,70,81^	**3** ^38,50,65^	**2** ^44,61^	**2** ^27,68^		
MCI versus control	**12** ^31,32,34,35,38-40,46,65,68,74,81^	**3** ^38,50,65^	**2** ^71,84,c^	**4** ^35,38,44,71^		
**YKL-40 (41):** **blood (11), CSF (36)**						
AD versus control	**4** ^41,90,103,104^	**2** ^52,90^	**1** ^105^		**19** ^30,53d,92,99,105-115,95a,103a,116a,117c^	
AD versus MCI	**3** ^41,90,103^	**8** ^49,52,103,109-112,117c^			**3** ^99,110,114^	**1** ^118^
MCI versus control	**5** ^41,71,90,103,94c^	**6** ^52,99,103,108,111,119a^	**2** ^101d,105^		**13** ^53e,71,90,105,109,112-115,118,120,94a,117c^	

Most frequently studied biomarkers in AD, ranked by number of studies. Biomarkers and total number of studies in bold. AD, Alzheimer’s disease; CN, cognitive normal; CSF, cerebrospinal fluid; EOAD, early-onset Alzheimer’s disease; IL, interleukin; LOAD, late-onset Alzheimer’s disease; MCI, mild cognitive impairment; MCP, monocyte chemoattractant protein; Ns., non-significant; TNF-α, tumour necrosis factor-alpha; YKL-40, chitinase-3-like protein 1.

^a^ Corrected for covariates; ^b^ Only in EOAD, not in LOAD; ^c^ Corrected for multiple testing; ^d^ Only in Hispanic subgroup; ^e^ AD and MCI higher than HC, but not higher than CN.

**Table 2:** PD results in different studies

	Number of studies by biofluid type					
Biomarker (number of studies)	Ns. Blood	Ns. CSF	↑ Blood	↓ Blood	↑ CSF	↓ CSF
**TNF-α (32):** **Blood (28), CSF (8)**						
PD versus control	**11** ^121-131a^	**5** ^127,128,132-134,b^	**10** ^131c,132,135-141,142^	**2** ^131d,143^	**1** ^143^	**1** ^134e^
Asymptomatic mutation carriers versus controls					**1** ^133^	
**IL-6 (30):** **Blood (26), CSF (9)**						
PD versus control	**13** ^121-129,138,143-145^	**7** ^127-129,133,134b,143,146^	**8** ^125,130,136,140,144f,147,148,142^		**1** ^134e^	
**CRP/hs-CRP (27/10): Blood (27/6), CSF (9/0)**						
PD versus control	**15** ^121,122,127,138,142b-144,147-154^	**5** ^127,143,146,152,155^	**CRP**: **5** ^139,144f,156,157,158g^; **hs-CRP**: **5** ^159-163h^	**CRP**: **1** ^155^		
**IL-10 (22):** **Blood (19), CSF (7)**						
Below detection limit	**2** ^143,164^	**3** ^133,143,165^				
PD versus control	**8** ^121,125,127-130,142b,166^	**4** ^127,128,130,167^	**3** ^123,131,138^	**1** ^140^		
**IL-1β (21):** **Blood (16), CSF (5)**						
Below detection limit	**2** ^142,143^	**3** ^133,143,165^				
PD versus control	**3** ^127,128,168b^	**3** ^127,128,134b^	**8** ^121,130,132,141,169-172^	**1** ^168e^	**2** ^132,134e^	
Asymptomatic mutation carriers versus controls			**1** ^173i^			
**WBC**: **LYM (20)**: **Blood (20); NEU (15)**: **Blood (15); LEUK (16)**: **Blood (16), CSF (1); Mono (12)**: **Blood (12); NLR (10)**: **Blood (10); Baso (4)**: **Blood (4); Eos (5)**: **Blood (4)**						
PD versus control	**LYM: 10** ^152,174-182i^; **NEU: 9** ^156,174-176,178,179,182ijk-184^; **Mono: 6** ^176-178,181,182i,k,183^; **LEUK: 5** ^162,176,182i,185,186,e^; **NLR: 2** ^162,174^; **Baso: 3** ^175,178,182l^; **Eos: 2** ^178,182l^	**LEUK: 1** ^152^	**LYM: 3** ^136,140,179^; **NEU: 5** ^153,162,177,181,182l^; **Mono: 2** ^158g,162^; **LEUK: 2** ^153,179^; **NLR: 5** ^158g,178,179,183,182i,j,k,l^	**LYM: 10** ^153,156,158g,162,177,178,181,183,184,182j,k,l^; **Mono: 1** ^182j,l^		
**IL-8 (19):** **Blood (16), CSF (9)**						
PD versus control	**7** ^123,127,128,142b,143,187,188^	**5** ^95,127,128,133,189m^	**2** ^125,164^	**1** ^143^	**1** ^146^	
**IFN-γ (16):** **Blood (13), CSF (8)**						
Unreliable detection	**2** ^128,173^	**4** ^128,133,165,173^				
PD versus control	**5** ^123,127,129,138,142b^	**3** ^127,134b,143^	**1** ^131^	**2** ^136,143^		**1** ^134e^
**IL-2 (14):** **Blood (12), CSF (5)**						
Below detection limit	**3** ^128,143,164^	**3** ^128,133,143^				
PD versus control	**4** ^121,127,135,142b^	**1** ^127^	**1** ^122^			
**MCP-1 (12):** **Blood (7), CSF (6)**						
PD versus control	**1** ^127^	**5** ^95,127,133,146,189^	**1** ^135^			

Most frequently studied biomarkers in PD, ranked by number of studies. Biomarkers and total number of studies in bold. Baso, basophils; CRP, C-reactive protein; CSF, cerebrospinal fluid; Eos, eosinophils; GBA, glucosylceramidase beta (glucocerebrosidase gene); HC, healthy controls; hs-CRP, high-sensitivity C-reactive protein; IFN-γ, interferon-gamma; IL, interleukin; LEUK, leucocytes; LYM, lymphocytes; LRRK, leucine-rich repeat kinase; MCP, monocyte chemoattractant protein; Mono, monocytes; NEU, neutrophils; NLR, neutrophil-to-lymphocyte ratio; Ns., non-significant; PD, Parkinson’s disease; PINK1, PTEN-induced kinase 1; RBD, REM sleep behavior disorder; sPD, sporadic Parkinson’s disease; TNF-α, tumour necrosis factor-alpha; WBC, white blood cell; YKL-40, chitinase-3-like protein 1.

^a^1–3 years PD; ^b^ PD without cognitive impairment; ^c^ < 1-year PD; ^d^ >3 years PD; ^e^ PD with cognitive impairment; ^f^ Symptomatic biallelic Parkin/PINK1 mutation carriers; ^g^ PD-RBD; ^h^ PD Stage 2 versus HC; ^i^ LRKK mutation carriers versus HC; ^j^ sPD versus HC; ^k^ GBA mutation carriers versus HC; ^l^ Combined PD versus HC in both cohorts; ^m^ After correction for mulitple comparisons.

**Table 3:** FTD results in different studies

	Number of studies by biofluid type			
Biomarker (number of studies)	Ns. Blood	Ns. CSF	↑ Blood	↑ CSF
**YKL-40 (8):** **Blood (1), CSF (7)**				
FTD versus control		**1** ^106^		**6** ^109,110,115,190-192^
Asymptomatic FTD versus control	**1** ^193a^			
Symptomatic versus asymptomatic FTD	**1** ^193b^			
Prodromal versus asymptomatic FTD	**1** ^193b^			
**TNF-α (6):** **Blood (4), CSF (3)**				
Below detection limit		**1** ^51^		
FTD versus control	**3** ^194,195c,196d^	**3** ^66,195,196d^		
Asymptomatic FTD versus control	**2** ^193a,195e^			
Symptomatic versus asymptomatic FTD	**1** ^193b^		**1** ^196f^	
Prodromal versus asymptomatic FTD			**1** ^193b^	
**IL-6 (6):** **Blood (4), CSF (3)**				
FTD versus control	**3** ^194,195g,196d^	**3** ^51d,66,196^	**1** ^195e^	
Asymptomatic FTD versus control	**1** ^193a^	**1** ^195e^		
Symptomatic versus asymptomatic FTD	**1** ^196f^	**1** ^196f^	**1** ^193b^	
Prodromal versus asymptomatic FTD	**1** ^193b^			
**IL-1β (5):** **Blood (3), CSF (3)**				
Below detection limit	**1** ^196d^	**2** ^51d,196^		
FTD versus control	**1** ^194^	**1** ^66^		
**IL-17A (5):** **Blood (3), CSF (3)**				
Below detection limit	**1** ^197^	**2** ^51d,196^		
FTD versus control	**1** ^196d^	**1** ^66^	**1** ^194^	
**IFN-y (5):** **Blood (3), CSF (3)**				
Below detection limit	**2** ^196d,197^	**2** ^51d,196^		
FTD versus control	**1** ^194^	**1** ^66^		
**IL-10 (4):** **Blood (2), CSF (2)**				
Below detection limit		**1** ^51^		
FTD versus control	**1** ^194^	**1** ^66^		
**MCP-1 (4):** **Blood (2), CSF (3)**				
FTD versus control	**1** ^196,d^	**3** ^51d,66,196^		
Symptomatic versus asymptomatic FTD	**1** ^196f^	**1** ^196,f^		
**IL-2 (4):** **Blood (2), CSF (3)**				
Below detection limit		**2** ^51d,196^		
FTD versus control	**1** ^196d^	**1** ^66^	**1** ^194^	
**IL-4 (4):** **Blood (2), CSF (3)**				
Below detection limit	**1** ^196d^	**2** ^51d,196^		
FTD versus control	**1** ^194^	**1** ^66^		
**IL-8 (4):** **Blood (2), CSF (3)**				
FTD versus control	**1** ^196d^	**3** ^51d,66,196^		
Symptomatic versus asymptomatic FTD	**1** ^196f^	**1** ^196,f^		
**IL-12p70 (4):** **Blood (2), CSF (3)**				
Below detection limit		**2** ^51d,196^		
FTD versus control	**1** ^196d^	**1** ^66^	**1** ^194^	

Most frequently studied biomarkers in FTD, ranked by number of studies. Biomarkers and total number of studies in bold. CHMP2B, charged multivesicular body protein 2B; CSF, cerebrospinal fluid; C9orf72, chromosome 9 open reading frame 72; FTD, frontotemporal dementia; GRN, granulin; IFN-γ, interferon-gamma; IL, interleukin; IL-12p70, interleukin-12 subunit p70; MAPT, microtubule-associated protein tau; MCP, monocyte chemoattractant protein; Ns., non-significant; TNF-α, tumour necrosis factor-alpha; YKL-40, chitinase-3-like protein 1.

^a^Participants with pathogenic C9orf72, GRN, or MAPT variants, compared with non-carrier family members; ^b^ Combined gene groups with pathogenic C9orf72, GRN, and MAPT variants; ^c^ With and without GRN mutation; ^d^ Combined symptomatic and pre-symptomatic CHMP2B mutation carriers versus non-carriers; ^e^ With GRN variant; ^f^ CHMP2B mutation carriers; ^g^ Without GRN mutation.

**Table 4:** ALS results in different studies

	Number of studies by biofluid type					
Biomarker (number of studies)	Ns. Blood	Ns. CSF	↑ Blood	↓ Blood	↑ CSF	↓ CSF
**IL-6 (13):** **Blood (9), CSF (3)**						
ALS versus control	**3** ^198-200^		**5** ^201-205^	**1** ^206^	**3** ^51,207,208^	
**TNF-α (11):** **Blood (9), CSF (2)**						
Below detection limit		**1** ^51^				
ALS versus control	**5** ^200,201,203,204,209^	**1** ^208^	**3** ^202,205,210^	**1** ^206^		
**IFN-y (10):** **Blood (8), CSF (2)**						
Below detection limit		**1** ^51^				
ALS versus control	**4** ^200,204,206,211^		**3** ^203,205,210^	**1** ^209^		**1** ^207^
**IL-10 (9):** **Blood (6), CSF (3)**						
Below detection limit		**1** ^51^				
ALS versus control	**3** ^200,201,204^	**1** ^208^	**1** ^205^	**2** ^203,206^		**1** ^207^
**MCP-1 (9):** **Blood (5), CSF (5)**						
ALS versus control	**3** ^198,204,212^	**1** ^51^	**1** ^213^	**1** ^206^	**4** ^207,213-215^	
**WBC: NLR (8):** Blood (8); **LEUK (6): Blood (5), CSF (1); Mono (6): Blood (6); LYM (5): Blood (4), CSF (1); NEU (4): Blood (4); NK cell (3): Blood (3), CSF (1)**						
ALS versus control	**NLR**: **2** ^200,208^; **LEUK**: **2** ^216,217^; **Mono**: **3** ^217-219^; **LYM**: **3** ^203,217,218^; **NEU**: **1** ^217^; **NK cell: 1** ^220^	**LEUK:** **1** ^219^; **NK cell:** **1** ^203^	**NLR: 2** ^216,218^**LEUK: 1** ^200^; **Mono: 1** ^221a^; **NEU: 1** ^218^; **NK cell:** **1** ^203^		**LYM: 1** ^203^	
**IL-8 (8):** **Blood (5), CSF, (8)**						
ALS versus control	**2** ^198,202^	**3** ^51,207,208^	**3** ^201,204,206^			
**CRP (7):** **Blood (5), CSF (2)**						
ALS versus control	**1** ^200^	**2** ^51,222^	**2** ^218,223^			
**IL-2 (7):** **Blood (4), CSF (3)**						
Below detection limit		**1** ^51^				
ALS versus control	**2** ^204,209^		**1** ^205^	**1** ^206^	**2** ^207,208^	
**YKL-40 (6):** **Blood (2), CSF (6)**						
ALS versus control	**2** ^224,225^	**1** ^224^			**5** ^214,225-228^	
**IL-1β (6):** **Blood (4), CSF (2)**						
Below detection limit		**1** ^51^				
ALS versus control	**1** ^200^	**1** ^208^	**2** ^203,209^	**1** ^206^		
**CHIT1 (6):** **Blood (1), CSF (5)**						
ALS versus control	**1** ^225^	**1** ^227^			**4** ^214,215,225,228^	
**IL-4 (5):** **Blood (3), CSF (2)**						
Below detection limit		**1** ^51^				
ALS versus control	**2** ^204,206^	**1** ^208^	**1** ^209^			
**MIP-1α (5):** **Blood (2), CSF (4)**						
Below detection limit		**1** ^51^				
ALS versus control		**1** ^208^	**2** ^207,229^	**1** ^206^	**1** ^229^	

Most frequently studied biomarkers in ALS, ranked by number of studies. Biomarkers and total number of studies in bold. ALS, amyotrophic lateral sclerosis; CHIT1, chitotriosidase-1; CRP, C-reactive protein; CSF, cerebrospinal fluid; IFN-γ, interferon-gamma; IL, interleukin; LEUK, leucocytes; LYM, lymphocytes; MCP, monocyte chemoattractant protein; MIP-1α, macrophage inflammatory protein-1 alpha; Mono, monocytes; NEU, neutrophils; NK cell, natural killer cell; NLR, neutrophil-to-lymphocyte ratio; Ns., non-significant; TNF-α, tumour necrosis factor-alpha; WBC, white blood cell; YKL-40, chitinase-3-like protein 1.

^a^ Compared to normal range.

**Table 5:** DLB, MSA, HD, and PSP results in different studies

	Number of studies by biofluid type					
Biomarker (number of studies)	Ns. Blood	Ns. CSF		↑ Blood	↓ Blood	
**(A) DLB**						
**IL-6 (6):** **Blood (5), CSF (1)**						
DLB versus control	**3** ^33,84,230^	**1** ^51^		**2** ^62,231^		
**IL-8 (6):** **Blood (4), CSF (2)**						
DLB versus control	**3** ^33,84,231^	**2** ^51,95^			**1** ^230^	
**TNF-α (5):** **Blood (4), CSF (1)**						
Below detection limit		**1** ^51^				
DLB versus control	**3** ^33,230,231^				**1** ^84^	
**IL-1β (5):** **Blood (4), CSF (1)**						
Below detection limit	**1** ^230^	**1** ^51^				
DLB versus control	**1** ^33^			**2** ^84,231^		
**IL-10 (5):** **Blood (4), CSF (1)**						
Below detection limit		**1** ^51^				
DLB versus control	**3** ^33,230,231^			**1** ^84^		
**IL-13 (5):** **Blood (4), CSF (1)**						
Below detection limit	**2** ^33,84^	**1** ^51^				
DLB versus control	**2** ^230,231^					
**(B) MSA**	**Ns. Blood**	**Ns. CSF**		**↑ Blood**	**↓ CSF**	
**CRP (4): Blood (3), CSF (1)**						
MSA versus control	**2** ^149,153^	**1** ^146^				
**MCP-1 (4): Blood (1), CSF (3)**						
MSA versus control	**1** ^135^	**3** ^146,167,232^				
**IL-8 (4): Blood (1), CSF (3)**						
MSA versus control	**1** ^135^	**2** ^146,167^			**1** ^189^	
**IL-6 (3): Blood (1), CSF (2)**						
MSA versus control	**1** ^135^	**2** ^146,167^				
**Fractalkine (3): Blood (2), CSF (1)**						
MSA versus control	**1** ^135^	**1** ^167^		**1** ^233^		
**TGF-α (3): Blood (1), CSF (2)**						
MSA versus control	**1** ^135^	**1** ^167^			**1** ^189^	
**(C) HD**	**Ns.** **Blood**	**Ns.** **saliva**	**Ns.** **CSF**	**↑** **Blood**	**↓** **Blood**	**↑** **Saliva**
**IL-6 (5):** **Blood (4), CSF (1), saliva (1)**						
Manifest HD versus HC		**1** ^234^		**3** ^234-236a^		
Premanifest HD versus HC	**3** ^234,236a,237^	**1** ^234^				
Premanifest & Manifest HD versus HC			**1** ^238^			
Manifest HD versus Premanifest HD						**1** ^234^
**CRP (3):** Blood (3), saliva (1)						
Manifest HD versus HC	**1** ^234^	**1** ^234^		**1** ^235^	**1** ^239^	
Premanifest HD versus HC	**2** ^234,239^					**1** ^234^
Manifest HD versus Premanifest HD	**1** ^234^	**1** ^234^				
**IL-1β (3):** Blood (3), saliva (1)						
Manifest HD versus HC	**1** ^234^					**1** ^234^
Premanifest HD versus HC	**2** ^234,237^	**1** ^234^				
Premanifest & Manifest HD versus HC			**1** ^238^			
Manifest HD versus Premanifest HD	**1** ^234^	**1** ^234^				
**(D) PSP**	**Ns. Blood**			**↑ Blood**	**↓ Blood**	
**NLR (4): Blood (4)**						
PSP versus controls				**2** ^183,240^	**1** ^241^	

Most frequently studied biomarkers in DLB and MSA and all biomarkers studied in HD and PSP, ranked by number of studies. Biomarkers, total number of studies and diseases in bold. CRP, C-reactive protein; CSF, cerebrospinal fluid; DLB, dementia with Lewy bodies; HD, Huntington’s disease; IL, interleukin; MCP, monocyte chemoattractant protein; MSA, multiple system atrophy; NLR, neutrophil-to-lymphocyte ratio; Ns., non-significant; PSP, progressive supranuclear palsy; TNF-α, tumour necrosis factor-alpha; TGF-α, transforming growth factor-alpha.

^a^ Corrected for covariates.

For consistency and comparability across diseases, the control groups included healthy individuals and cognitively normal (CN) participants, as well as individuals with non-neurodegenerative conditions; in Alzheimer’s disease/MCI studies, it also included participants with subjective cognitive decline (SCD) who did not meet study criteria for MCI or Alzheimer’s disease. For group comparisons, the specific biofluid used is indicated in the table. For all other analyses, unless otherwise specified, results are reported from blood. Blood refers collectively to plasma, serum, or whole blood, which were combined due to the large number of studies and to improve comparability.

In the following section, we focus on eight biomarkers (IL-6, TNF-α, IL-1β, (hs-)CRP, IL-10, MCP-1, YKL-40, WBC) that were most frequently assessed across diseases, providing more detailed results.

#### Interleukin-6

**Figure 2:**
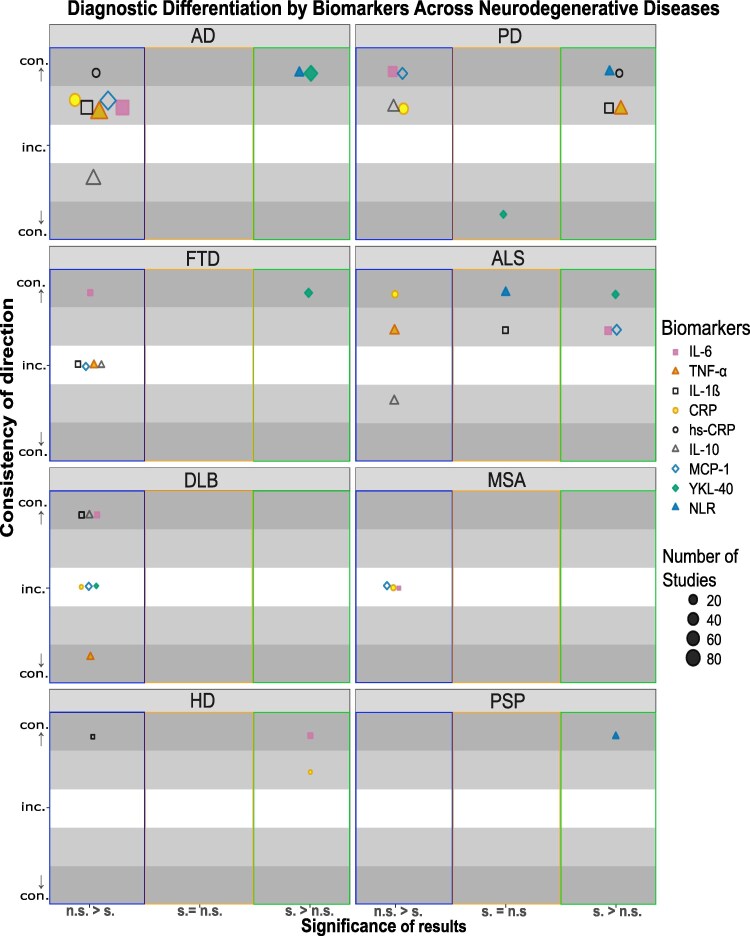
**Diagnostic differentiation by biomarkers across neurodegenerative diseases.** Performance of biomarkers for distinguishing disease from controls, shown separately for each neurodegenerative disease. Shape size indicates the number of studies, shape and colour represent different biomarkers. The *x*-axis reflects the proportion of significant results: left (n.s. > s., blue outline) indicates more non-significant than significant results, middle (s. = n.s., orange outline) indicates an equal number of significant and non-significant results, and right (s. > n.s., green outline) indicates more significant than non-significant results. The *y*-axis shows the consistency of direction among significant results, with the top arrow (↑) indicating fully consistent higher biomarker levels in diseases (dark grey), the bottom arrow (↓) indicating fully consistent lower biomarker levels in diseases (dark grey), the middle indicating either inconsistent direction of significant results or no significant results (white), and intermediate positions reflecting partial consistency (light grey), with the overall direction based on the majority of reported findings. AD, Alzheimer’s disease; ALS, amyotrophic lateral sclerosis; con., consistent; CRP, C-reactive protein; DLB, dementia with Lewy bodies; FTD, frontotemporal dementia; HD, Huntington’s disease; hs-CRP, high-sensitivity C-reactive protein; IL, interleukin; inc., inconsistent; MCP, monocyte chemoattractant protein; MSA, multiple system atrophy; NLR, neutrophil-to-lymphocyte ratio; n.s., non-significant; PD, Parkinson’s disease; PSP, progressive supranuclear palsy; s., significant; TNF-α, tumour necrosis factor-alpha; YKL-40, chitinase-3-like protein 1.

Interleukin-6 (IL-6), a pro-inflammatory cytokine which mediates acute-phase responses and regulates immune cell recruitment, is the most extensively studied neuroinflammation biomarker across neurodegenerative diseases, reported in 83 Alzheimer’s disease, 30 Parkinson’s disease, 13 amyotrophic lateral sclerosis, 6 frontotemporal dementia, 6 dementia Lewy body, 5 Huntington’s disease and 3 multiple system atrophy studies. In the majority of the studies, IL-6 was assessed in blood. Significant differences were observed in 44 studies (41% of IL-6 studies examining group differences), with the majority (41 studies, 93%) showing higher IL-6 levels in patients compared with controls, as reported in Tables 1–5. Importantly, in amyotrophic lateral sclerosis and Huntington’s disease, the majority of the studies showed significant differences of which most studies showed a significantly higher level in patients compared with controls (Fig. 2). A single Alzheimer’s disease saliva study, not included in the table, found significantly lower IL-6 concentrations in Alzheimer’s disease and MCI compared with controls, and lower levels in Alzheimer’s disease versus MCI.^242^ One study using the ATN classification found significantly higher CSF IL-6 levels in the A+T−APOE4 group compared with healthy controls and the A+T+APOE4 group, while no differences were observed between A+T+Alzheimer’s disease patients and healthy controls irrespective of APOE genotype.^243^ Three additional CSF ATN studies reported no significant differences between A−T−(N−), A+T−(N−), A+T+(N−/+) groups.^244-246^

Receiver operating characteristic (ROC) analyses demonstrated robust discriminatory ability of IL-6, with Area under the Curve (AUC) values ranging from 0.648 to 0.77 for distinguishing Alzheimer’s disease,^29,67^ with the highest value (0.77) measured in CSF^67^; 0.97 in a small Parkinson’s disease cohort^140^; and 0.933 for amyotrophic lateral sclerosis^205^ from controls. However, two Alzheimer’s disease studies reported that inclusion of IL-6 did not improve predictive models for disease classification.^46,247^ In amyotrophic lateral sclerosis, higher IL-6 levels were also associated with an increased likelihood of diagnosis.^204^ Population-based Parkinson’s disease studies further showed that IL-6 was higher in established female cases compared with controls, with no difference for newly diagnosed Parkinson’s disease,^145^ and another study found a positive association with Parkinson’s disease risk.^147^

##### Disease stages

Within-disease comparisons showed higher IL-6 levels in Alzheimer’s disease compared with MCI in five studies,^22,61,68-70^ and one study demonstrated progressive increases across advancing Alzheimer’s disease stages.^64^ In Huntington’s disease, one saliva study found significantly higher levels in manifest compared with premanifest gene expansion carriers,^234^ and also in frontotemporal dementia, one study reported a significantly higher level in symptomatic compared with asymptomatic mutation carriers.^193^ In Parkinson’s disease, one study found lower levels in later stages, and with longer disease duration.^130^ Four Parkinson’s disease studies found no correlation with disease duration,^126,143,144,165^ although one reported a significant association in PRKN/PINK1 mutation carriers but not in idiopathic Parkinson’s disease.^144^

##### Cross-sectional clinical correlations

Significant clinical correlations were identified across several diseases, with higher IL-6 levels consistently corresponding to worse clinical outcomes. In Alzheimer’s disease, higher IL-6 was negatively correlated with cognitive scores.^29,33,70,74,75,83,248,249^ In Parkinson’s disease, higher levels were associated with increased motor severity,^124,140,148^ overall disease severity^140,148^ and depression scores.^250^ Two studies (one in plasma, one in CSF^134^) reported significantly higher IL-6 levels in Parkinson’s disease patients with cognitive impairment compared with those without.^134,138^ In amyotrophic lateral sclerosis, IL-6 correlated positively with disease burden^202^ and negatively with clinical assessments.^199,205,251^ In behavioural variant frontotemporal dementia, one study found positive associations with both cognition and disease severity.^194^ In dementia Lewy body, higher IL-6 levels were linked to more severe cognitive impairment.^33^ In Huntington’s disease, IL-6 was negatively correlated with functional capacity scales,^236^ and saliva IL-6 showed significant correlations with a functional capacity scale and motor scales.^234^ In contrast, numerous studies reported no significant associations. Sixteen Alzheimer’s disease studies found no correlations with cognitive^21,37,40,42,45,59,70,142,252-259^ or functional scores^45,255^ of which two were measured in CSF.^255,256^ Eight Parkinson’s disease studies reported no associations with clinical measures^121,125-127,138,143,146,165^ or disease severity,^126,127,143^ including two conducted in CSF^146,165^ and two in both CSF and blood.^127,143^ One frontotemporal dementia study found no correlation with cognitive impairment regardless of PGRN mutation status^195^ and one dementia Lewy body study reported no associations with clinical assessments.^231^ No significant associations with disease severity were observed in one Huntington’s disease study^235^ and one multiple system atrophy study.^167^

##### Correlation with longitudinal disease progression

Higher IL-6 levels were generally positively correlated to disease progression. In Alzheimer’s disease, higher IL-6 was linked to progression of dementia,^260^ earlier age of onset^37^ and faster cognitive decline,^66^ of which the last study was measured in CSF.^66^ One study reported that higher levels were associated with an increased risk of cognitive decline over a 5-year period in models adjusted for age and sex;^261^ whereas another found that, in APOE ε4 non-carriers, higher CSF IL-6 levels were associated with better memory outcomes over time.^262^ In Parkinson’s disease, higher IL-6 was associated with a greater cognitive decline,^138^ faster progression of non-motor symptom severity^263^ and more rapid disease progression.^130^ In amyotrophic lateral sclerosis, IL-6 was positively associated with progression rate^205^ and in frontotemporal dementia, higher baseline IL-6 was associated with steeper decline among C9orf72 mutation carriers.^193^ Conversely, seven Alzheimer’s disease studies found no correlation with progression to MCI,^259^ or cognitive decline;^69,72,249,252,253,258,264^ of which one was conducted in CSF^72^ and one in both blood and CSF.^264^ Three studies reported that inclusion of IL-6 did not improve models for prediction of disease progression,^265-267^ of which one was done in CSF.^265^ In Parkinson’s disease, two studies reported no associations with progression^125,138^ and one found no correlation between CSF IL-6 and cognitive decline.^165^ In frontotemporal dementia, one study found no significant difference in IL-6 levels between non-converters and converters.^193^

**Figure 3:**
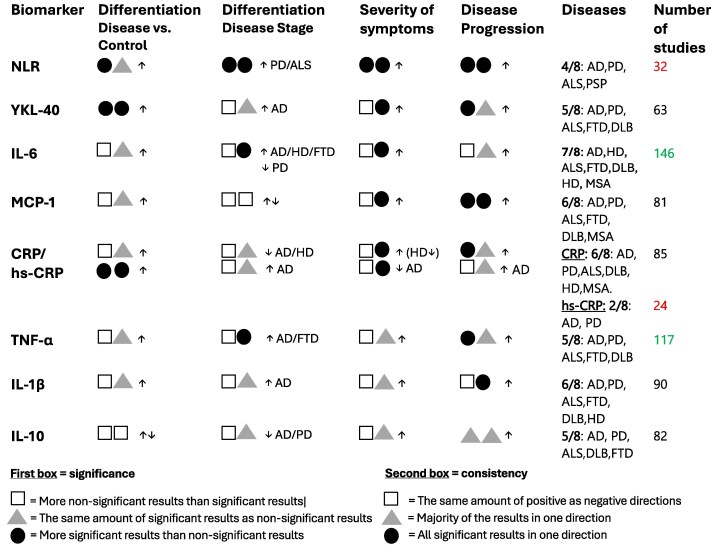
**Significance and medium-specific consistency of results across studies.** Performance of biomarkers in differentiating disease from controls, across disease stage and duration, and in relation to severity and progression. Shape and colour indicate the robustness of significance and consistency among significant results, as shown in figure. Arrows indicate the direction of change: ↑ indicates higher levels in disease versus controls, higher levels in advanced versus early or pre-symptomatic stages, and higher levels corresponding to worse outcomes or faster progression; ↓ indicates lower levels in disease versus controls, lower levels in advanced versus early or pre-symptomatic stages, and lower levels corresponding to worse outcomes or faster progression. AD, Alzheimer’s disease; ALS, amyotrophic lateral sclerosis; CRP, C-reactive protein; DLB, dementia with Lewy bodies; FTD, frontotemporal dementia; HD, Huntington’s disease; hs-CRP, high-sensitivity C-reactive protein; IL, interleukin; MCI, mild cognitive impairment; MCP, monocyte chemoattractant protein; MSA, multiple system atrophy; NLR, neutrophil-to-lymphocyte ratio; PD, Parkinson’s disease; PSP, progressive supranuclear palsy; TNF-α, tumour necrosis factor-alpha; YKL-40, chitinase-3-like protein 1.

As summarized in Fig. 3, IL-6 is the most studied neuroinflammatory marker, typically elevated in patients and in more advanced stages and showing moderate to good diagnostic utility in present studies, aside from one Parkinson’s disease study reporting lower levels with longer disease duration. Although higher IL-6 often relates to worse clinical status and faster progression, many studies find no significant differences or associations, limiting its clinical value.

#### Tumour necrosis factor-alpha

Tumour necrosis factor-alpha (TNF-α), an innate immune molecule that plays a key role in maintaining cellular, tissue and overall homeostasis, was analysed in 63 Alzheimer’s disease , 32 Parkinson’s disease, 11 amyotrophic lateral sclerosis, 6 frontotemporal dementia and 5 dementia Lewy body studies. In the majority of the studies, TNF-α was assessed in blood. Significant group differences were observed in 38 studies (41% of all TNF-α studies examining group differences), of which the majority (31 studies, 82%) showed significantly higher levels in patients compared with controls, as presented in Tables 1–5. Importantly, in Parkinson’s disease, the majority of the studies showed significant differences of which most studies showed a significantly higher level in patients compared with controls (Fig. 2). An Alzheimer’s disease saliva study found significantly lower level in both Alzheimer’s disease and MCI compared with controls, and in Alzheimer’s disease relative to MCI.^242^ Two CSF studies applying the ATN classification found no significant differences in TNF-α between A−T−(N−), A+T−(N−), A+T+(N−/+).^243,244^

Reported ROC analyses demonstrated that TNF-α shows moderate to high discriminatory ability. In Alzheimer’s disease, AUC values for distinguishing patients from controls ranged from 0.587 to 0.766, with the highest measured in CSF.^67,76^ For differentiating MCI from controls, the AUC was lower (0.562).^29^ In multivariate prediction models, some studies concluded that TNF-α was one of the best markers to include,^31,40,82^ whereas others excluded it from their optimal model,^46,247,268^ of which one measured TNF-α in CSF.^268^ In Parkinson’s disease, AUC values ranged from 0.66 (ref. ^269^) to 1.00,^140^ with one study reporting 0.794 for disease onset <1 year,^131^ and another CSF study finding 75% correct classification of individuals into Parkinson’s disease or control groups.^143^ In a population-based study, TNF-α was higher in established male cases, but no difference was observed for newly diagnosed Parkinson’s disease compared with controls.^145^

##### Disease stages

Within the Alzheimer’s disease spectrum, four studies reported significantly higher TNF-α levels in Alzheimer’s disease compared with MCI.^33,70,79,80^ One Parkinson’s disease study showed that TNF-α decreased with increasing disease duration,^131^ whereas a CSF study found a positive correlation with disease duration,^165^ and a second CSF study found no correlation with disease duration.^143^ In frontotemporal dementia, TNF-α was significantly higher in symptomatic^196^ and prodromal^193^ compared with asymptomatic mutation carriers.

##### Cross-sectional clinical correlations

Significant clinical correlations were limited, with heterogeneous results in Alzheimer’s disease. TNF-α level was positively correlated with the Mini-Mental State Examination (MMSE) in one Alzheimer’s disease study,^82^ but negatively correlated with cognition in several others.^29,33,42,83^ In the other diseases, the significant outcomes were more consistent. In Parkinson’s disease, TNF-α levels were positively associated with motor severity and disease stage,^140^ and one study reported a negative correlation with cognition.^138^ In amyotrophic lateral sclerosis, TNF-α showed mild positive correlations with disease burden.^202^ In behavioural variant frontotemporal dementia, TNF-α levels were positively associated with disease severity,^194^ and in CHMP2B mutation carriers, TNF-α correlated positively with disease burden.^196^ In dementia Lewy body, higher TNF-α levels were linked to greater severity of cognitive impairment.^33^ In contrast, several studies reported no significant correlations. Nine Alzheimer’s disease studies found no associations with cognitive scores.,^21,29,79,142,252-254^ Ten Parkinson’s disease studies reported no correlation with motor symptoms, cognition or disease severity,^121,126,127,134,140,143,165,263,269,270^ of which two studies were performed in both CSF and blood^127,143^ and two were measured in CSF.^134,165^ One frontotemporal dementia study found no correlation with cognitive impairment,^195^ and one dementia Lewy body study reported no association with clinical assessments.^231^

##### Correlation with longitudinal disease progression

Findings on TNF-α and disease progression were heterogeneous in Alzheimer’s disease but more consistent in other neurodegenerative diseases. In Alzheimer’s disease, higher CSF TNF-α was positively associated with progression to Alzheimer’s disease^271^ and higher blood TNF-α was positively correlated to progression of dementia.^64,272^ In contrast, another study found significantly lower levels in the progressed group.^40^ Several Alzheimer’s disease studies found no significant difference between converters and non-converters^260,271^ or between slow and fast decliners,^253^ and three studies reported no correlation with cognitive decline,^69,252,264^ of which one was performed in CSF.^264^ In Parkinson’s disease, TNF-α levels were positively associated with cognitive decline^138,165^ and disease progression rate,^130^ of which two studies were performed in CSF.^130,165^ In amyotrophic lateral sclerosis, TNF-α was positively associated with progression rate.^205^ In frontotemporal dementia, higher baseline TNF-α was associated with more rapid cognitive decline, worsening of disease severity and conversion with an AUC of 0.72 among pathogenic C9orf72, GRN or MAPT mutation carriers.^193^

TNF-α is the second most studied neuroinflammatory marker, typically elevated in patients and showing variable diagnostic utility. Although higher TNF-α is often linked to worse clinical status and faster progression, numerous non-significant and inconsistent findings, particularly in Alzheimer’s disease, limit its overall clinical value (Fig. 3).

#### Interleukin-1β

IL-1β, an pro-inflammatory molecule involved in cell proliferation, apoptosis and differentiation, was measured in 50 Alzheimer’s disease, 21 Parkinson’s disease, 6 amyotrophic lateral sclerosis, 5 frontotemporal dementia, 5 dementia Lewy body and 3 Huntington’s disease studies. In the majority of the studies, IL-1β was assessed in blood. Detection was below the limit of detection in seven blood studies^21,34,69,142,143,196,230^ and nine CSF studies.^30,49,51,52,71,133,143,165,196^ Significant differences were reported in 36 studies (47% of all IL-1β studies examining group differences), of which 33 studies showed a higher level in patients compared with controls. Notably, in Parkinson’s disease, the majority of the studies showed significant differences of which 91% showed a significantly higher level in patients compared with controls. More details are provided in Tables 1–5 and Fig. 2. One CSF study using the ATN classification found no significant differences in IL-1β between A+T−, A+T+ and healthy controls, regardless of APOE genotype.^243^

Reported ROC analyses demonstrated modest discriminative ability for IL-1β. In Alzheimer’s disease, one CSF study reported an AUC of 0.713 for distinguishing patients from controls.^67^ For differentiating MCI from controls, one study reported an AUC of 0.595.^29^ IL-1β was excluded from prediction models that differentiated Alzheimer’s disease from controls^247^ and MCI from controls^46,268^ in three studies. However, it was included in the best-performing model that distinguished MCI from controls in another study.^75^ In Parkinson’s disease, one study reported an AUC of 0.618 for differentiating patients from controls.^141^

##### Disease stages

Across the disease stages, findings were inconsistent. Four studies reported significantly higher IL-1β levels in Alzheimer’s disease compared with MCI,^37,70,86,88^ whereas two found significantly lower levels in Alzheimer’s disease relative to MCI.^33,35^ One Alzheimer’s disease study reported increased IL-1β levels with advancing disease stages.^64^ In Parkinson’s disease, one study found a significant positive association with disease duration^141^ and another study found a negative association.^171^ In Huntington’s disease, there was no significant difference between manifest and premanifest gene expansion carriers.^234^

##### Cross-sectional clinical correlations

In Alzheimer’s disease, IL-1β was not consistently associated with cognition across all tests, and correlations were not always in the same direction. Some studies suggested that higher IL-1β was associated with better cognition, showing positive correlations with the digit span test,^29^ and in MCI, with MoCA.^75^ One study found that lower IL-1β levels were associated with greater cognitive impairment.^33^ In contrast, other Alzheimer’s disease studies indicated that higher IL-1β was associated with worse cognition or function, reporting positive correlations with functional impairment,^26^ negative correlations with MMSE,^70^ and negative associations with memory performance.^83^ Another study found negative correlations between IL-1β in both CSF and blood and MMSE for Alzheimer’s disease and MCI, and with MoCA in blood for Alzheimer’s disease and in CSF for MCI.^87^ In Parkinson’s disease, two studies reported positive correlations with motor scores,^141,172^ and two found positive associations with disease severity.^130,172^ Conversely, another study found significantly lower IL-1β CSF levels in patients with cognitive impairment compared with Parkinson’s disease without cognitive impairment.^134^ In dementia Lewy body, two studies also reported significant correlations between lower IL-1β levels and more severe cognitive impairment^33,84^ and in one study with higher motor scores.^84^ In Huntington’s disease, higher saliva IL-1β levels correlated significantly with worse functional capacity and more severe motor impairment.^234^ Numerous studies reported no significant cross-sectional associations. Seven Alzheimer’s disease studies found no correlation with cognitive scores;^21,26,37,142,252-254^ eight Parkinson’s disease studies reported no correlation with clinical measures,^121,127,128,130,134,168,170,171,173^ and five Parkinson’s disease studies reported no association with disease severity;^121,127,128,168,169,173^ and one dementia Lewy body study found no correlation with clinical assessments.^231^

##### Correlation with longitudinal disease progression

Higher IL-1β levels were generally associated with disease progression. In Alzheimer’s disease, higher CSF IL-1β levels were associated with a greater cognitive decline after 1 year^66^ and higher blood IL-1β with a more rapid decline.^69^ In Parkinson’s disease, two studies reported positive correlations between IL-1β and faster decline.^130,263^ Similarly, in amyotrophic lateral sclerosis, IL-1β levels were significantly higher in fast progressors compared with slow progressors, and showed a positive correlation with disease progression.^203^ Five Alzheimer’s disease studies showed no correlation with cognitive decline,^69,252,253,264,272^ of which one was performed in both CSF and blood,^264^ and in Parkinson’s disease, one study showed no direct correlation with disease progression.^169^

As illustrated in Fig. 2, IL-1β is a frequently studied neuroinflammatory marker, generally higher in patients, though detection in blood and CSF is often unreliable and its discriminative utility is moderate. Elevated IL-1β is generally associated with faster progression, but clinical associations are often inconsistent or non-significant, limiting its overall clinical value.

#### C-reactive protein and high-sensitivity C-reactive protein

C-reactive protein (CRP), an acute-phase protein reflecting immune activation, was investigated in 41 Alzheimer’s disease, 27 Parkinson’s disease, 7 amyotrophic lateral sclerosis, 4 multiple system atrophy, 3 dementia Lewy body and 3 Huntington’s disease studies. High-sensitivity (hs)-CRP, a more sensitive assay for CRP, was measured in 14 Alzheimer’s disease and 10 Parkinson’s disease studies. In the majority of the studies, CRP was assessed in blood. For the studies with non-significant results between groups, these markers were combined in the tables. Significant group differences were reported in 20 CRP studies and 8 hs-CRP studies (resp. 28% and 67% of all CRP/hs-CRP studies examining group differences).

For CRP, 15 of these studies showed higher levels in patients compared with controls. More details are documented in Tables 2, 4, 5B and C and Supplementary Tables 2 and 6. When looking at individual diseases, in Huntington’s disease, all 3 studies showed significant results, 2/3 showed a significantly higher level in disease compared with controls (Fig. 2). One CSF study using the ATN classification reported no significant differences in CRP between A−T−, A+T− and A+T+ groups.^246^ hs-CRP levels were always higher in patients than in controls, as outlined in Table 2 and Supplementary Table 1. In Parkinson’s disease, all five studies showed significantly higher hs-CRP levels in disease compared with controls (Fig. 2).

ROC analyses indicated limited discriminatory value of CRP for differentiating Alzheimer’s disease from controls, with AUCs of 0.539 (ref. ^273^) and 0.559,^274^ and classification accuracy of 58.6–59.5% in a CSF study.^51^ In prediction models for distinguishing disease from controls, CRP improved the model in one study,^268^ but not in others,^46,103^ of which one was performed in CSF.^103^ In Parkinson’s disease, reported AUCs ranged from 0.683 (ref. ^156^) to 0.721,^275^ although CRP did not improve prediction models in other studies.^164,180^ A population-based Alzheimer’s disease study reported that individuals with CRP levels in the highest quartile had a 1.148-fold increased risk of dementia.^268^ In Parkinson’s disease, two population-based studies reported no association between CRP and newly diagnosed Parkinson’s disease,^180,276^ whereas two other studies suggested lower CRP levels were linked to increased Parkinson’s disease risk.^145,277^

For hs-CRP, ROC analyses revealed various levels of discriminatory value. In Alzheimer’s disease, one study reported an AUC of 0.590 in differentiating cases from controls.^76^ In Parkinson’s disease, AUC ranged from 0.701 (ref. ^159^) to 0.907.^162^ Higher hs-CRP was also associated with increased odds of Alzheimer’s disease,^278^ while another study found a reduced risk, correcting for BMI and weight changes, though this effect disappeared after adjusting for APOE ε4 status^279^ and a third showed no significant association with risk for Alzheimer’s disease.^267^

##### Disease stages

Across disease stages, results were inconsistent for CRP. In Alzheimer’s disease, CRP levels were significantly higher compared with MCI in one study,^70^ but lower in three others.^46,52,280^ One study reported significantly lower CRP in both mild and moderate Alzheimer’s disease compared with healthy controls,^281^ while another showed a 3.6-fold increase in moderate Alzheimer’s disease and a 16-fold increase in advanced Alzheimer’s disease compared with age-matched controls.^282^ One Alzheimer’s disease study^103^ and two Parkinson’s disease studies^143,283^ found no significant association with disease duration, of which two studies were performed in both blood and CSF.^103,143^ In Huntington’s disease, CRP was significantly higher in early stages compared with more advanced stages.^235^

hs-CRP levels were higher in Alzheimer’s disease compared with MCI^284^ and in Parkinson’s disease, stage 2 patients had significantly higher levels compared with controls as well as compared with stages 1, 3, and 4.^163^ In Parkinson’s disease, one study showed no correlations with disease duration.^159^

##### Cross-sectional clinical correlations

CRP was generally associated with worse clinical outcome. In Alzheimer’s disease, higher CRP was associated with worse cognition and function,^51,70,285,286^ of which two were assessed in CSF.^51,285^ In Parkinson’s disease, higher CRP was linked to worse motor symptoms,^146,157^ cognition,^157^ psychiatric symptoms,^146,283^ and overall disease severity,^146,156,157^ of which one study was performed in CSF.^146^ CSF CRP was significantly higher in Parkinson’s disease patients with dementia compared with Parkinson’s disease without dementia,^146^ and blood CRP was significantly higher in Parkinson’s disease with MCI compared with Parkinson’s disease without MCI.^138^ In amyotrophic lateral sclerosis, higher CRP correlated with greater motor impairment^221^ and in multiple system atrophy, higher CRP levels were linked to worse cognition, functional scales, and disease severity.^149^ In contrast, in Huntington’s disease, CRP correlated positively with cognitive performance and functional capacity.^234^ Several studies reported no significant associations. Six Alzheimer’s disease studies found no correlation between CRP and cognitive scores,^52,58,70,83,257,258,281^ of which one study was performed in CSF.^52^ In Parkinson’s disease, four studies reported no significant association with motor severity,^127,138,143,283^ cognition,^127,138^ or disease severity,^127,143^ of which two studies were conducted in both CSF and blood.^127,143^ One Huntington’s disease study found no correlation between CRP and advancing disease stage, functional capacity or apathy score.^239^ In saliva, CRP levels were not significantly correlated with clinical assessments or functional capacity.^234^

For hs-CRP, little evidence was found for correlations with clinical outcome. One Alzheimer’s disease study reported that higher hs-CRP levels were associated with better cognitive performance and functional outcomes,^287^ but other studies found no association between hs-CRP and cognition^42,284,287,288^ or functional scores.^284,288^ In Parkinson’s disease, hs-CRP showed no correlations with motor symptoms,^160,263^ or disease severity.^148,159^

##### Correlation with longitudinal disease progression

With respect to disease progression, significant results were quite heterogeneous for CRP. In Alzheimer’s disease, two studies found significantly lower CRP in MCI converters compared with stable MCI, with findings remaining significant after adjustment for covariates or multiple comparison.^289,290^ In contrast, another study reported that higher CRP levels were associated with steeper cognitive declines after adjusting for demographic and cardiovascular risk factors.^291^ In Parkinson’s disease, high CRP was associated with increased risk of death, disease severity and rapid disease progression,^292^ with a greater motor worsening over time^138,293^ and greater cognitive decline.^154^ In amyotrophic lateral sclerosis, CRP levels positively correlated with mortality and hospitalization,^294^ and disease progression;^223^ while another study found no association with survival.^295^ In multiple system atrophy-C, but not multiple system atrophy-P, there was a significant positive correlation with mortality.^296^ Six Alzheimer’s disease studies found no correlation with MCI-to-Alzheimer’s disease conversion,^260,280,290^ or cognitive decline,^69,258,264,280,285,297^ of which one study was performed in both CSF and blood^264^ and one was performed in CSF.^285^ One Parkinson’s disease study found no correlation with cognitive decline.^138^

Longitudinal studies of hs-CRP showed inconsistent results. In Alzheimer’s disease, higher hs-CRP levels were associated with slower cognitive decline^288^ and slower functional decline,^288^ whereas another study reported that higher levels predicted greater decline in functional scores.^284^ Other studies reported no association with cognitive decline.^261,287^ In Parkinson’s disease, hs-CRP showed no correlations with cognitive decline.^161^

As outlined in Fig. 3, CRP is a frequently studied inflammatory marker, generally higher in patients than controls, though many findings are non-significant and its discriminative utility is modest. Elevated CRP is typically associated with worse clinical outcomes, with the exception of one study in Huntington’s disease in which the associations were reversed. hs-CRP has been investigated less frequently and only in Alzheimer’s disease and Parkinson’s disease but shows better differentiation for disease and disease stage. One Alzheimer’s disease study reported a significant association between higher hs-CRP levels and more favourable outcomes. Longitudinal findings for both CRP and hs-CRP in Alzheimer’s disease show conflicting results, limiting their prognostic value, whereas in Parkinson’s disease, amyotrophic lateral sclerosis and multiple system atrophy, CRP is more consistently positively associated with disease progression.

#### Interleukin-10

The anti-inflammatory marker IL-10, which inhibits pro-inflammatory cytokines, was measured in 42 Alzheimer’s disease, 22 Parkinson’s disease, 9 amyotrophic lateral sclerosis, 5 dementia Lewy body and 4 frontotemporal dementia studies. In the majority of the studies, IL-10 was assessed in blood. Detection was below the limit of detection in three blood studies^69,143,164^ and seven CSF studies.^49,51,52,71,133,143,165^ Significant differences were reported in 22 studies (34% of all IL-10 studies examining group differences), with approximately equal numbers reporting higher or lower levels in patients versus controls, and no disease showing significant results in more than 50% of studies, as detailed in Tables 1–5. Three CSF studies applying the ATN classification reported no significant differences in IL-10 between A−T−(N−), A+T−(N−), and A+T+(N−/+) groups.^243,244,246^

Limited reported ROC analyses suggested good discriminative ability for IL-10. In Alzheimer’s disease, one CSF study reported an AUC of 0.73 for distinguishing patients from controls.^66^ However, three studies did not include IL-10 in their optimal prediction models for differentiating Alzheimer’s disease from controls.^46,247,268^ In Parkinson’s disease, one study reported an AUC of 0.94 for distinguishing patients from controls.^140^

##### Disease stages

Within the Alzheimer’s disease spectrum, results were inconsistent: Two studies found significantly higher IL-10 in Alzheimer’s disease compared with MCI,^44,61^ whereas two others reported significantly lower levels in Alzheimer’s disease relative to MCI.^27,68^ In Parkinson’s disease, IL-10 levels significantly decreased across H&Y stages.^140^ Other Parkinson’s disease studies reported no significant associations with disease duration,^127,131,166^ of which one was performed in CSF and blood.^127^

##### Cross-sectional clinical correlations

Significant cross-sectional findings were also inconsistent in direction. In Alzheimer’s disease, higher CSF IL-10 levels were positively correlated with cognitive status,^66^ and higher blood levels correlated negatively with cognitive scores.^83,298^ In Parkinson’s disease, one study found a positive correlation between blood IL-10 and anxiety and depression, but no associations with cognitive function, motor symptoms, or disease severity; CSF IL-10 showed no clinical correlations.^130^ However, another study reported that reduced IL-10 was significantly associated with greater motor impairment.^140^ In amyotrophic lateral sclerosis, IL-10 was positively correlated with forced vital capacity (FVC).^205^ In contrast, several studies reported no significant cross-sectional associations. In Alzheimer’s disease, two studies found no correlation with cognitive scores.^142,254^ In Parkinson’s disease, five studies reported no significant associations with clinical assessments^121,127,131,138,166^ and disease severity,^131,138^ of which one study was assessed in both CSF and blood.^127^ In dementia Lewy body, one study reported no correlation with clinical assessments.^231^

##### Correlation with longitudinal disease progression

Longitudinal findings on IL-10 were also mixed. In Alzheimer’s disease, higher baseline IL-10 was significantly associated with greater cognitive decline,^40,66^ of which one study was conducted in CSF.^66^ One Alzheimer’s disease study reported significantly higher IL-10 levels in rapid decliners compared with slow decliners,^32^ another found no difference,^69^ and a third reported higher levels in patients who remained stable.^260^ In Parkinson’s disease, one study found higher IL-10 levels to be associated with faster cognitive decline.^263^ In contrast, in frontotemporal dementia, lower levels correlated with higher longitudinal functional decline rate.^197^ Two Alzheimer’s disease studies found no significant correlation with cognitive decline,^69,264^ of which one was done in CSF and blood^264^ and one Parkinson’s disease study reported no significant association with motor and cognitive decline.^138^

As summarized in Fig. 3, IL-10 shows poor discriminatory ability across diseases and stages. Associations with severity and disease progression vary by outcome and disease, and are often non-significant or inconsistent, limiting its clinical and prognostic value.

#### Monocyte chemoattractant protein-1

Monocyte chemoattractant protein-1 (MCP-1), also known as CCL2 (C-C motif chemokine ligand 2), is a chemokine that regulates migration and infiltration of monocytes. It was measured in 48 Alzheimer’s disease, 12 Parkinson’s disease, 9 amyotrophic lateral sclerosis, 4 frontotemporal dementia, 4 multiple system atrophy and 4 dementia Lewy body studies. Studies were approximately evenly distributed between CSF and blood. Significant differences were reported in 23 studies (38% of all MCP-1 studies examining group differences), with a majority showing higher levels in disease compared with controls. In amyotrophic lateral sclerosis, most studies demonstrated significant group differences (*n* = 5), with four reporting elevated levels in patients compared with controls. Details are reported in Tables 1–5, Supplementary Table 6 and Fig. 2. One CSF study applying ATN classification reported significantly higher MCP-1 levels in A+T+ compared with A+T−, but not compared with A−T−; this significance was lost after adjustment for multiple comparisons.^246^ Another CSF study found no significant differences in MCP-1 between A+T−, A+T+, and healthy controls, irrespective of APOE genotype.^243^ A further plasma study reported no significant difference between the A+ and A− group.^299^

The diagnostic performance of MCP-1, as assessed by ROC analyses, was highly variable. In Alzheimer’s disease, reported AUC values for distinguishing patients from controls ranged from 0.503 to 0.61,^89,92,247^ of which the lowest and the highest AUC were obtained in CSF studies.^89,92^ One study identified CSF MCP-1 as the most consistently selected parameter across 10 predictive models for differentiating Alzheimer’s disease from controls.^264^ MCP-1 also showed potential as an additive marker, with multivariate models achieving AUCs of 0.58–0.90 for differentiating Alzheimer’s disease from controls, and 0.56–0.93 for differentiating Alzheimer’s disease from MCI,^46,268^ of which one was assessed in CSF.^268^ In Parkinson’s disease, MCP-1 was not retained in an optimal prediction model for differentiating patients from controls.^139^

##### Disease stages

Findings across disease stages were inconsistent. Within the Alzheimer’s disease spectrum, one study reported higher MCP-1 levels in Alzheimer’s disease compared with MCI,^96^ whereas two studies found lower levels in Alzheimer’s disease compared with MCI.^31,46^ In Parkinson’s disease, lower CSF levels were observed in Parkinson’s disease dementia compared with Parkinson’s disease without dementia.^146^ Another CSF study reported a positive correlation with disease duration, but only in females.^165^ In amyotrophic lateral sclerosis, one study reported that MCP-1 levels increased with longer symptom duration.^212^ In frontotemporal dementia, no significant difference between symptomatic and asymptomatic mutation carriers was found in both CSF and blood.^196^

##### Cross-sectional clinical correlations

Cross-sectional significant associations were consistently showing worse performance with higher levels. In Alzheimer’s disease, one study reported negative correlations of CSF MCP-1 with cognitive scores, while blood levels showed no such associations.^255^ In Parkinson’s disease, one CSF study found a positive correlation with motor score, but only in females, and also reported negative correlations with cognitive performance.^165^ In contrast, four Alzheimer’s disease studies reported no significant correlations with cognitive scores,^92,254,256,300^ of which one was performed in both CSF and blood^256^ and one in CSF.^92^ Two Parkinson’s disease CSF studies showed no associations with motor scores^146^ or disease severity.^146,165^ In multiple system atrophy, CSF MCP-1 levels were not correlated with disease severity.^167^

##### Correlation with longitudinal disease progression

Longitudinal significant findings for MCP-1 supported a positive association with disease progression. In Alzheimer’s disease, three studies demonstrated a significant association between higher MCP-1 levels and greater cognitive decline,^96,300,301^ with one study observing this effect in CSF but not in blood.^301^ Higher MCP-1 levels in CSF were also significantly associated with shorter time to conversion from MCI to Alzheimer’s disease^301^; and overall disease progression.^264^ All longitudinal findings in Alzheimer’s disease were adjusted for relevant confounders. In Parkinson’s disease, one CSF study showed a positive correlation between MCP-1 and disease progression based on H&Y scores over 3 years; however, this association was no longer present at 10-year follow-up, and there were no correlations with motor or cognitive progression.^232^ In frontotemporal dementia, higher MCP-1 levels showed a positive correlation with cognitive decline.^197^ Other studies reported no significant associations with cognitive decline, in Alzheimer’s disease,^272^ and in CSF in MCI^72^ and Parkinson’s disease.^165^ In amyotrophic lateral sclerosis, CSF MCP-1 did not predict survival.^215^

MCP-1 is an inflammatory marker for which patient–control and stage differences are often inconsistent. Cross-sectional clinical associations are mostly non-significant, but when present, higher MCP-1 reflects worse performance. Longitudinally, higher MCP-1 is more reliably linked to faster progression, with significant findings outweighing null results, suggesting potential prognostic value (Fig. 3).

#### Chitinase-3-like protein 1

A relative new marker in neurodegenerative research, YKL-40 also known as chitinase-3-like protein 1 (CHI3L1), is a predominantly glial glycoprotein associated with tissue remodelling and neuroinflammatory processes. It was analysed in 41 Alzheimer’s disease, 8 frontotemporal dementia, 5 Parkinson’s disease, 6 amyotrophic lateral sclerosis and 3 dementia Lewy body studies. In the majority of the studies, YKL-40 was assessed in CSF. Significant differences were reported in 38 studies (73% of all YKL-40 studies examining group differences), with 97% showing higher levels in patients compared with controls (only one CSF study showed lower levels in Parkinson’s disease versus controls). These findings are presented in Tables 1, 3 and 4, Supplementary Tables 2 and 6. In the majority of the Alzheimer’s disease, frontotemporal dementia and amyotrophic lateral sclerosis studies, results were significantly different, showing consistent higher levels in disease compared with controls (Fig. 2). Two CSF studies applying the ATN classification reported higher YKL-40 levels in A+T+ compared with A−T− and A+T−, but found no difference between A+T− and A−T−.^120,245^ Another CSF study found significantly higher YKL-40 in groups with A−T+/−N+/− and A+T+/−N+/− compared with healthy controls (A−T−N−) and the A+T−N− group. The A−T+/−N+/− group also showed higher levels than the A−T−N− group with clinical symptoms of Alzheimer’s disease or MCI.^106^ A further CSF study reported significantly higher YKL-40 in A+T+, with or without SCD, compared with A+/−T−.^113^ A plasma study reported no significant difference between the A+ and A− groups.^299^

Reported AUC values highlight the diagnostic potential of YKL-40 across neurodegenerative diseases. In Alzheimer’s disease, AUCs for distinguishing patients from controls ranged from 0.67 to 0.88,^90,92,95,103,104,106,109^ of which six out of seven were performed in CSF.^90,92,95,103,106,109^ Higher CSF YKL-40 levels in CN controls were associated with increased risk of developing Alzheimer’s disease,^90^ with one population-based study reporting a 12–59% higher risk per standard deviation increase after multivariable adjustment.^302^ However, some studies reported no additional value of YKL-40 for disease classification or progression beyond established diagnostic groups,^109^ neurodegeneration markers,^48,265^or Alzheimer’s disease-specific biomarkers,^110^ of which three out of four studies were conducted in CSF.^109,110,265^ Reported AUCs for distinguishing patients from controls in other diseases were 0.92 for Parkinson’s disease;^303^ 0.79–0.83 for frontotemporal dementia^190,191,193^ of which two studies were analysed in CSF^190,191^; 0.80 for amyotrophic lateral sclerosis (CSF)^225^ and 0.74 for dementia Lewy body.^104^

##### Disease stages

Within the Alzheimer’s disease spectrum, findings were less consistent. Three CSF studies reported higher YKL-40 levels in Alzheimer’s disease compared with MCI,^99,110,114^ whereas one CSF study found lower levels in Alzheimer’s disease versus MCI.^118^ In frontotemporal dementia, no significant difference between symptomatic and prodromal and asymptomatic mutation carriers was found^193^ and in CSF no significant association with disease duration was reported.^192^

##### Cross-sectional clinical correlations

Significant cross-sectional associations were reported across diseases, with higher YKL-40 levels generally corresponding to worse clinical outcomes. In Alzheimer’s disease, higher YKL-40 levels were associated with worse cognition and function,^108,114,302,304,305^ of which four out of five studies were assessed in CSF.^108,114,304,305^ In Parkinson’s disease, one CSF study found a negative correlation with MMSE.^95^ In amyotrophic lateral sclerosis, higher CSF YKL-40 levels were associated with worse motor scores^225^ and higher blood YKL-40 levels were correlated with lower FVC.^224^ In contrast, several studies reported no significant associations: Six Alzheimer’s disease studies found no correlation with cognitive scores,^92,95,104,285,305,306^ of which four out of six were analysed in CSF.^92,95,285,305^ One Parkinson’s disease CSF study found no correlation with motor or cognitive scores or disease severity;^146^ and in CSF of FTD^191^ and in blood of dementia Lewy body,^104^ no correlation was observed with MMSE.

##### Correlation with longitudinal disease progression

With respect to disease progression, significant results were more inconsistent. In Alzheimer’s disease, higher CSF YKL-40 levels were observed in MCI patients who subsequently progressed to Alzheimer’s disease compared with those who remained stable.^114,117^ One CSF study reported an AUC of 0.62 for predicting conversion from MCI to Alzheimer’s disease.^116^ However, contradictory findings were also reported, including greater cognitive decline in Alzheimer’s disease patients with lower baseline CSF YKL-40^117^ and improved performance on a memory test—but no effect on MMSE or other cognitive assessments—associated with increasing blood YKL-40 levels over 1 year.^306^ Four Alzheimer’s disease studies found no significant associations with cognitive decline,^105,307^ risk of disease progression^308^ or conversion after adjustment for covariates.^309^ Two of these studies were performed in CSF^307,309^ and one in both CSF and blood.^105^ In amyotrophic lateral sclerosis, CSF YKL-40 levels were positively associated with disease progression;^224,226,227^ one CSF study identified it as a significant predictor of mortality,^227^ whereas another CSF study found no correlation with survival.^225^ In frontotemporal dementia, among mutation carriers (C9orf72, GRN or MAPT), higher baseline YKL-40 was associated with faster deterioration in the ability to perceive and respond to others’ emotions and social cues,^193^ but found no significant difference between non-converters and converters.^193^

As illustrated in Figs 2 and 3, YKL-40 shows higher levels in patients in the majority of studies. When significant, cross-sectional associations consistently link higher YKL-40 to worse outcomes, suggesting clinical utility. Longitudinally, higher baseline levels are usually associated with faster progression, though findings in Alzheimer’s disease are somewhat inconsistent.

#### Neutrophil-to-lymphocyte ratio

Across neurodegenerative diseases, the most frequently investigated white blood cell-related marker was the neutrophil-to-lymphocyte ratio (NLR), which serves as an indicator of systemic immune balance, capturing both innate immune activity through neutrophils and adaptive immune function through lymphocytes. It has been assessed in 10 Alzheimer’s disease, 10 Parkinson’s disease, 8 amyotrophic lateral sclerosis and 4 progressive supranuclear palsy studies. Almost all studies were assessed in blood, using routine blood tests. Significant group differences were found in 15 studies (75% of all NLR studies examining group differences), in most Alzheimer’s disease and Parkinson’s disease studies, and in all progressive supranuclear palsy studies (Fig. 2), reporting consistently higher NLR in patients than controls. More details are provided in Tables 2 and 5D and Supplementary Table 2). In Alzheimer’s disease, NLR distinguished patients from controls with an AUC = 0.787.^273^ High NLR increased the odds of Alzheimer’s disease and MCI,^310^ and another study confirmed that MCI and Alzheimer’s disease were both independently associated with higher NLR, also after exclusion of systemic inflammatory disorders.^311^ In progressive supranuclear palsy, NLR demonstrated an AUC of 0.754 for distinguishing disease from controls.^240^

##### Disease stages

In Parkinson’s disease, longer disease duration was associated with higher NLR,^178^ and in amyotrophic lateral sclerosis, higher NLR was associated with a more advanced disease stage.^312^

##### Cross-sectional clinical correlations

Across diseases, higher NLR generally correlated with worse outcomes. NLR was negatively associated with cognition in Alzheimer’s disease^313,314^ and MCI.^310^ In Parkinson’s disease, higher NLR was linked to greater motor impairment and^182,315^ increased disease severity.^179,315^ In amyotrophic lateral sclerosis, higher NLR^316,317^ was associated with reduced functional capacity and positively correlated with respiratory failure^312^ and disease severity.^318^ In progressive supranuclear palsy, NLR was negatively correlated with cognitive performance.^319^ Several studies, however, found no significant associations. In Alzheimer’s disease, some reports detected no correlations between NLR and cognitive or functional outcomes.^310,311^ In Parkinson’s disease, several studies observed no significant association between NLR and cognition,^315^ motor scores^178,320^ or disease severity.^178,182^

##### Correlation with longitudinal disease progression

Longitudinal associations were consistent for NLR. In Alzheimer’s disease, higher baseline NLR was associated with subsequent cognitive decline,^311^ and with conversion from MCI to Alzheimer’s disease.^314^ Another study found no significant differences in NLR between progressing and stable MCI groups.^310^ In amyotrophic lateral sclerosis, NLR correlated positively with disease progression,^312,316,317^ faster progression rates^317^ and higher NLR tertiles were associated with a 3-fold higher mortality risk.^316^ Another study found no association between NLR and survival.^295^

As shown in Figs 2 and 3, NLR, is examined less often than most other biomarkers, but shows many significant results with high consistency. Most studies reported higher NLR in patients, and stage comparisons in Parkinson’s disease and amyotrophic lateral sclerosis showed increasing levels with advancing disease. Both cross-sectional and longitudinal studies more often linked higher NLR to worse outcomes and faster disease progression than not, supporting its clinical and prognostic utility.

#### Disease-specific neuroinflammation biomarkers

sTREM-2, a microglial transmembrane receptor involved in regulating neuroinflammation and neurodegeneration, was measured in ≥3 cohorts only in Alzheimer’s disease, mostly in CSF. Significant differences were reported in 10 studies (56%), generally showing higher levels in Alzheimer’s disease compared with MCI and controls, and in MCI compared with controls (Supplementary Table 2). TREM2 has been investigated in CSF studies applying the ATN classification. One study reported significantly higher levels in A+/−T+ groups compared with A+/−T− groups, and higher levels in the dementia group with A− than in the dementia group with A+.^321^ Another study reported higher levels in A+T+ compared with A+T−, but not compared with A−T−, whereas A+T− showed significantly lower levels compared with A−T−.^322^ A further study reported significantly higher levels in A+T+ compared with A+/−T−, with no significant difference between A+T− and A−T−.^245^ Another study reported significantly higher levels in the A+T+N+ group compared with healthy controls (A−T−N−), A+T−N− and A+T+N− groups.^323^ Regarding diagnostic performance, ROC analyses reported an AUC of 0.78 for distinguishing Alzheimer’s disease from controls in both CSF^107^ and blood,^104,107^ while another CSF study measured a classification accuracy of 58.6–59.5% for differentiating MCI or Alzheimer’s disease patients from cognitively unimpaired individuals.^51^ One CSF study reported a significant positive association with cognitive performance^285^ and two CSF studies showed that higher sTREM2 levels predicted less future cognitive decline, after adjusting for covariates^50^ and independently of CSF t-tau.^264^ However, six studies showed no correlation with cognitive scores,^104,321,324^ conversion^321,325^ or cognitive decline^264,321,323^ and two other studies found that sTREM-2 did not improve predictive models for disease progression.^265,308^ Six of these eight studies were performed in CSF.,^264,265,321,323-325^ sTREM2 was not assessed in the other diseases, included in this review.

Another biomarker in Alzheimer’s disease research yielding notable findings is Glial Fibrillary Acidic Protein (GFAP), an astrocyte structural protein, of which the majority was assessed in blood (Supplementary Table 2). Significant differences were found in nine studies (62.5%), all showing higher levels in Alzheimer’s disease compared with MCI and controls, and in MCI versus controls, except for a single saliva study that reported lower GFAP levels across these comparisons. This saliva study also reported excellent diagnostic performance, with AUCs of 0.998–1.00 for Alzheimer’s disease versus controls, 1.00 for MCI versus controls, and 0.805–0.865 for Alzheimer’s disease versus MCI.^242^ Regarding ATN studies, one CSF study reported a significantly higher GFAP level in the A+T+ group compared with A+T− and A−T− groups, but no significant difference between A+T− and A−T−.^245^ A plasma study reported a significantly higher level in A+ compared with A−.^299^ Regarding clinical correlations, one study found negative associations between CSF and blood GFAP and memory but not executive function,^108^ another study also reported negative correlations with cognitive scores.^272^ The saliva study showed a positive correlation with MMSE, but only when analysed across the entire cohort.^242^ With respect to disease progression, one study showed that both higher GFAP levels and greater longitudinal increase were associated with earlier onset of MCI symptoms.^308^ Three studies found no correlation with cognitive scores^95,104,304^ of which one was conducted in CSF.^95^ One study found no correlation with cognitive decline^272^ and another study reported that CSF GFAP did not enhance predictive models for disease progression.^265^ GFAP was not measured in ≥3 cohorts in the other diseases included in this review.

In Parkinson’s disease, complement C3 and HMGB1 were additional biomarkers that more often yielded significant than non-significant results (Supplementary Table 3). Complement C3 also showed predominantly significant results in Alzheimer’s disease. In Alzheimer’s disease, six studies showed a significantly higher level of complement C3 in blood^46,275,326,327^ or CSF,^94,328^ and one study showed a significantly lower level^281^ in Alzheimer’s disease compared with controls (Supplementary Table 2). However, in Parkinson’s disease, two out of three studies with group comparisons showed a significantly lower level of this biomarker in blood.^153,329^ All three HMGB1 studies showed a significantly higher level in Parkinson’s disease compared with controls.^137,159,330^

In amyotrophic lateral sclerosis, additional biomarkers with more significant than non-significant results included IL-2, CHIT1, MIP-1α, G-CSF, IL-17 and IL-15; most of these markers showed a higher level in amyotrophic lateral sclerosis compared with controls (Table 4 and Supplementary Table 5). In the other studied neurodegenerative diseases, these markers did not show more significant than non-significant results.

## Discussion

This scoping review mapped the current landscape of neuroinflammation biomarkers in major neurodegenerative diseases, with the aim of assessing their consistency, overlap and potential clinical value. Across Alzheimer’s disease, Parkinson’s disease, amyotrophic lateral sclerosis, frontotemporal dementia, Huntington’s disease, dementia Lewy body, multiple system atrophy and progressive supranuclear palsy, it is clear that immune dysregulation is a recurring feature. Overall, NLR, IL-6, (hs-)CRP, TNF-α, IL-1β and IL-10 have been most frequently assessed in blood, YKL-40 has been more frequently assessed in CSF and MCP-1 showed more significant findings in CSF.

Across neuroinflammation biomarkers, both the strength of evidence and coverage across diseases varied (Figs 2 and 3). Taken together, these findings suggest that NLR is promising but under-represented across diseases. YKL-40 offers moderate diagnostic value and reflects severity and progression. Although extensively studied, IL-6 generally shows limited significance, but when associations reach significance, they are remarkably consistently higher. MCP-1, CRP and TNF-α appear more suited for tracking disease severity and/or progression. IL-10 and IL-1β show inconsistent results. Among the disease-specific neuroinflammation markers, GFAP showed particularly promising findings in Alzheimer’s disease. It may also be informative in other neurodegenerative conditions and warrants further cross-disease evaluation, as GFAP has been assessed in such contexts.^331^ These findings should be interpreted with caution, as they are based on patterns of significant versus non-significant results and their consistency across studies, rather than on formal quality scoring. In addition, the number of studies per biomarker differs substantially between diseases, with the literature dominated by studies in Alzheimer’s disease and Parkinson’s disease, outweighing the other neurodegenerative diseases. The number of studies per biomarker for each disease is reported in the disease-specific tables, illustrating the density of evidence across conditions.

Many neuroinflammatory biomarkers have both central and peripheral origins, which influences their interpretation in biofluids. White blood cells originate in the bone marrow are normally present in peripheral blood at defined reference ranges in healthy individuals, with elevations above these limits and shifts in specific subtypes reflecting systemic immune activation.^332^ CRP is another predominantly peripheral marker, produced in the liver in response to inflammatory cytokines such as IL-6.^333^ Cytokines such as IL-6, TNF-α and IL-1β, as well as YKL-40 and MCP-1, are produced both by glial and peripheral cells.^334-340^ Similarly, IL-10, an anti-inflammatory cytokine, can be secreted by microglia and peripheral regulatory T and B cells.^341^ CSF levels generally reflect a central secretion, whereas plasma levels can reflect a combination of systemic and CNS-associated inflammation. Both sources can provide clinically relevant information, as systemic inflammation often accompanies neurodegenerative diseases and correlates with disease progression.^342,343^ Systemic inflammation may also be triggered by glial overactivation in the CNS, resulting in the release of various peripheral inflammatory mediators.^337,344,345^ In addition, changes in blood-brain barrier permeability enable biomarker exchange between compartments.^345^ Regardless of origin, changes in biofluid levels can serve as indicators of disease presence, severity and progression, as reflected by our results.

Although evidence suggests that neuroinflammation is more pronounced in disease compared with controls and is already elevated in the transition phase, its timing within disease progression remains unclear. Overall, levels tend to increase with advancing stages, though some studies report the opposite, and correlations with disease duration are inconsistent and different per disease, preventing firm conclusions. In studies applying the ATN framework, significant differences in neuroinflammatory markers were more often associated with tau pathology than with Aβ pathology.^106,113,120,243,245,246,322^ This suggests that neuroinflammation may be more closely linked to downstream disease processes, including tau pathology and neurodegeneration, rather than to early amyloid-β pathology. However, firm conclusions are limited by the small number of studies using the ATN framework. Disease-specific pathophysiology, including alpha-synuclein versus amyloid/tau pathology, may further contribute to distinct inflammatory profiles. Similarly, in frontotemporal dementia, phenotypic and genetic forms were combined for pragmatic reasons, but this approach may obscure biologically distinct inflammatory patterns in specific mutation subgroups.

In addition, several methodological and clinical considerations related to the included studies should be taken into account when interpreting the findings and designing future studies. Some studies include participants with SCD, who report symptoms but do not meet diagnostic criteria for MCI or Alzheimer’s disease. Nevertheless, they may still differ from healthy controls, for instance showing elevated blood TNF-α^79^ and CSF sTREM-2.^99^ Other studies include CN patients recruited for clinical indications such as lumbar puncture, rather than healthy controls. These groups may also diverge from healthy controls, with higher blood osteopontin and IL-1β reported.^71^ Such distinctions are critical when defining inclusion criteria, although in practice control groups are rarely entirely healthy, as their clinical presentation prompted clinical assessment.

Although blood-based biomarkers are often promoted to improve accessibility and reduce diagnostic disparities, only a minority of included studies explicitly reported participant race, ethnicity or socioeconomic characteristics.^47,101,346^ This limited reporting restricts assessment of generalizability across diverse populations and highlights the need for more inclusive cohorts in future biomarker research. To minimize the influence of transient systemic inflammation, some studies excluded participants with elevated CRP^21,31,247^ and/or white blood cell counts.^31,247^ Nevertheless, this approach may also reduce sensitivity to disease-related inflammatory changes, as these markers can reflect underlying pathology and progression. Circulating inflammatory markers may also be influenced by external factors such as medications,^347^ which can confound associations and were not accounted for in many studies.

Variations in laboratory and analytical methods can substantially influence biomarker measurements and study outcomes. Detection methods strongly affect reliability, particularly for low-abundance markers such as IL-1β. One study found Single Molecule Array (SIMOA) the most sensitive and reliable among MSD V-PLEX, S-PLEX, and SIMOA, and clearly distinguishes Alzheimer’s disease patients from controls.^348^ Consistent with this, IL-1β was frequently reported as below the limit of detection in included studies using MSD V-PLEX assays.^30,51,142^ Similarly, another study reported that bead-based platforms, including SIMOA, outperform other assays for low-level cytokines, while MSD V-PLEX is more suitable for abundant markers.^349^ In line with this, only 1 of the 12 studies using V-PLEX reported levels below the limit of detection for the more abundant markers IL-6, TNF-α, MCP-1 and CRP.^51^ These findings underscore that available assays differ in suitability across biomarkers. Timing of sample collection also matters: many inflammatory factors show diurnal fluctuations, although TNF-α proved relatively stable.^143^ Given the wide range of available assays, this review does not detail methods used in individual studies. However, it is important to recognize that the choice of method can influence results. For example, if we look at IL-6, the most investigated neuroinflammation biomarker, we see significant results most often in studies using ELISA,^29,37,55-58,62-64,67,73,74,130,134,136,140,144,148,162,202,234-236,270^ more than when Luminex multiplex assays,^54,68,204-208^ or MSD electrochemiluminescence assays^263^ were used. Three studies using the more recent SIMOA method^26,44,47^ did not report significant findings in Alzheimer’s disease. Assay variability remains a major limitation for clinical translation, with biomarker performance strongly influenced by platform choice. Initiatives such as the Alzheimer's Association Global Biomarker Standardization Consortium and the Global Neurodegeneration Proteomics Consortium are working to harmonize methodologies and improve cross-study comparability, which will be essential for future clinical implementation of neuroinflammatory biomarkers.^350^ Biomarker levels may also vary depending on the biofluid used (e.g. plasma, serum or whole blood). These peripheral biofluids were combined in this review to allow comparison across studies, which may affect interpretation of the results. For IL-10, which showed approximately equal numbers of studies reporting higher or lower levels in patients versus controls, we observed both higher^28,61,123,131,138^ and lower^35,38,60,68,140,203,206^ levels in serum, suggesting that the inconsistency in findings is not explained by the type of biofluid. It should be noted that we did not use a systematic approach, as this is a scoping review. Finally, statistical approaches varied across studies, as some studies corrected for covariates and multiple comparisons, while others did not or did not report their statistical methods. Importantly, future analyses should account for age, as ageing itself can lead to elevated neuroinflammatory biomarkers.^351,352^

One limitation of this review is its focus on neuroinflammation biofluid biomarkers, excluding other biomarker types due to the vast scope of the literature. However, biomarkers related to oxidative stress, hormonal regulation, vascular function or genetic markers, may directly or indirectly influence neuroinflammatory processes. A comprehensive discussion of neuroinflammation is inherently limited, as numerous interacting factors shape its complex pathophysiology. Cell and animal studies were excluded to maintain comparability across human studies, which may have omitted important insights into neuroinflammatory mechanisms and biomarker pathways.

Another limitation is that dementia Lewy body was not included as a specific search term, which may have resulted in missing relevant studies, despite its inclusion in the pre-defined eligibility criteria. Neuroimaging markers, although valuable for assessing neuroinflammatory processes, including microglial activation,^61,68,123,230,237,353-376^ and glial-related metabolic changes,^377^ were also excluded, as outlined in the Methods. The relatively small number of MRI and PET studies found due to our search strategy emphasizes the need to conduct a separate review on these imaging studies in the future, using a different search strategy. Biomarkers reported in <3 independent cohorts were excluded from analysis, consistent with our methodology. This limitation may have excluded findings that could still provide valuable insights for future studies.

Due to the scoping review design, we did not formally assess or weigh the methodological quality of the included studies, nor did take sample size into account. Consequently, large studies with null findings and smaller studies with positive findings are treated equally in our analysis, which is a limitation of this approach. Another potential limitation is that studies with different sample sizes were treated as separate in this review, as cohort-based research is common. However, some may have included shared participants, potentially leading to apparent replication. Both male and females were included in all included studies. However, detailed information on numbers of included participant sex was not systematically extracted from the 388 included studies, and is therefore not reported in the current manuscript. Finally, the included studies may be subject to publication bias, as studies reporting significant or positive findings are more likely to be published, potentially overestimating the apparent utility of some biomarkers. However, numerous studies with non-significant results were also reported in this review.

We presented and evaluated individual markers; however, combining markers often improved performance.^31,40,131^ For example, multivariate models integrating CSF and serum markers outperformed demographic and genetic reference models in Alzheimer’s disease,^268^ and adding imaging further increased Alzheimer’s disease classification accuracy beyond cytokines alone.^378^ Population-based data also showed that aggregated inflammatory scores predicted cognition and dementia risk, independent of APOEε4 or comorbidities.^346,379^ In Parkinson’s disease, a composite inflammatory score was a significant predictor of 36-month cognitive decline, leading the authors to conclude that higher inflammation was associated with disease progression.^138^ Nevertheless, neurodegeneration^29,48,265,380^ and disease-specific biomarkers^107,110,265^ often remained superior. Neurofilament Light (NfL) consistently outperformed inflammatory markers for both disease classification and correlations with severity and progression across multiple studies.^29,265,380^ In some cases, combining neuroinflammation with disease-specific markers improved predictive performance.^105,116,143^ Many studies report correlations between inflammatory markers and other biomarkers, particularly tau and amyloid markers. However, these associations may be influenced by covariance among CSF proteins.^381^ In this review, we focused primarily on associations between inflammatory markers and clinical outcomes rather than correlations between biomarkers.

Neuroinflammation is a key mechanism across neurodegenerative diseases, reflected by consistent differences in inflammatory markers between patients and controls. We presented an overview of the most extensively studied neuroinflammation biomarkers in neurodegenerative diseases. In clinical practice, neuroinflammatory biomarkers, such as NLR, YKL-40 and MCP-1, are not likely to replace established neurodegeneration or disease-specific biomarkers, but may serve as complementary markers to reflect inflammatory disease activity, disease severity, progression and heterogeneity across patients. Beyond diagnostic and prognostic applications, neuroinflammatory biomarkers may also be relevant in therapeutic research, providing objective measures of target engagement and treatment response. Their incorporation into clinical trials could facilitate the evaluation of anti-inflammatory and disease-modifying therapies,^4,4,382-386^ as well as anti-inflammatory effects of dietary and lifestyle interventions.^387,388^ Future studies should include longitudinal follow-up of clinically well assessed individuals before, during, and after symptom conversion, and in course of the disease, to define when neuroinflammatory processes emerge and how they evolve. Harmonization of assay methodologies, as highlighted above, will be critical to ensure that future findings are comparable and clinically translatable.

## Supplementary Material

fcag289_Supplementary_Data

## Data Availability

The search strategy, detailed inclusion and exclusion criteria, and a reference list of the 31 articles which were excluded based on biomarker threshold, are provided in the Supplementary material. Furthermore, the visual presentation of the data in Fig. 2 is based on a R code, which is provided in the Supplementary material. The figure was subsequently refined and edited in Inkscape for the final visual presentation.
