# Supplementary material for: Tracing neuroinflammation in neurodegeneration: insights from a scoping review on biofluid biomarkers
Source: Brain Commun. 2026 Aug 6;8(4):fcag289. doi: 10.1093/braincomms/fcag289 (PMC13472171; doi:10.1093/braincomms/fcag289)
Supplement: Supplementary file 1 [file fcag289_supplementary_data.pdf]

# SUPPLEMENTARY MATERIALS

**List of Abbreviations**

| Abbreviation | Definition |
|---|---|
| A (ATN) | Amyloid |
| AD | Alzheimer's Disease |
| ALS | Amyotrophic Lateral Sclerosis |
| APOE ε4 | Apolipoprotein E epsilon 4 |
| ATN | Amyloid – Tau – Neurodegeneration |
| AUC | Area Under the Curve |
| Baso | Basophils |
| BMI | Body Mass Index |
| C3 | Complement Component 3 |
| C4 | Complement Component 4 |
| C9orf72 | Chromosome 9 Open Reading Frame 72 |
| CAA | Cerebral Amyloid Angiopathy |
| CCL-23 | C-C Motif Chemokine Ligand 23 |
| CHIT1 | Chitotriosidase-1 |
| CHMP2B | Charged Multivesicular Body Protein 2B |
| CN | Cognitively Normal |
| CNS | Central Nervous System |
| CRP | C-Reactive Protein |
| CSF | Cerebrospinal Fluid |
| DLB | Dementia with Lewy Bodies |
| DM | Diabetes Mellitus |
| DSM-IV-TR | Diagnostic and Statistical Manual of Mental Disorders, fourth edition, text revision |
| EOAD | Early-Onset Alzheimer's Disease |
| Eos | Eosinophils |
| ESR | Erythrocyte Sedimentation Rate |
| FTD | Frontotemporal Dementia |
| FVC | Forced Vital Capacity |
| GBA | Glucosylceramidase Beta (Glucocerebrosidase gene) |
| G-CSF | Granulocyte Colony-Stimulating Factor |
| GFAP | Glial Fibrillary Acidic Protein |
| GM-CSF | Granulocyte-Macrophage Colony-Stimulating Factor |
| GRN | Granulin |
| H&Y stages | Hoehn and Yahr stages |
| HC | Healthy Controls |
| HD | Huntington's Disease |
| HIV | Human Immunodeficiency Virus |
| HMGB1 | High Mobility Group Box 1 |
| hs-CRP | High-sensitivity C-Reactive Protein |
| IC | Intensive Care |
| ICAM-1 | Intercellular Adhesion Molecule-1 |
| IFN-γ | Interferon-gamma |
| IgG | Immunoglobulin G |
| IL-10 | Interleukin-10 |
| IL-12 | Interleukin-12 |
| IL-12/IL-23p40 | Interleukin-12/Interleukin-23 subunit p40 |

| | |
|---|---|
| IL-12p40 | Interleukin-12 subunit p40 |
| IL-12p70 | Interleukin-12 subunit p70 |
| IL-13 | Interleukin-13 |
| IL-15 | Interleukin-15 |
| IL-16 | Interleukin-16 |
| IL-17 | Interleukin-17 |
| IL-17A | Interleukin-17A |
| IL-18 | Interleukin-18 |
| IL-1RA | Interleukin-1 Receptor Antagonist |
| IL-1α | Interleukin-1 alpha |
| IL-1β | Interleukin-1 beta |
| IL-2 | Interleukin-2 |
| IL-3 | Interleukin-3 |
| IL-33 | Interleukin-33 |
| IL-4 | Interleukin-4 |
| IL-5 | Interleukin-5 |
| IL-6 | Interleukin-6 |
| IL-7 | Interleukin-7 |
| IL-8 | Interleukin-8 |
| IL-9 | Interleukin-9 |
| IP-10 | Interferon Gamma-Induced Protein 10 (C-X-C Motif Chemokine Ligand 10, CXCL10) |
| LEUK | Leukocytes |
| LOAD | Late-Onset Alzheimer's Disease |
| LRRK | Leucine-Rich Repeat Kinase (commonly referring to *LRRK2*) |
| LYM | Lymphocytes |
| MAPT | Microtubule-Associated Protein Tau |
| MCI | Mild Cognitive Impairment |
| MCP-1 | Monocyte Chemoattractant Protein-1 |
| MCP-4 | Monocyte Chemotactic Protein-4 |
| MDC | Macrophage-Derived Chemokine |
| MIF | Macrophage Migration Inhibitory Factor |
| MIP-1α | Macrophage Inflammatory Protein-1 alpha |
| MIP-1β | Macrophage Inflammatory Protein-1 beta |
| MMP-1 | Matrix Metalloproteinase-1 |
| MMP-2 | Matrix Metalloproteinase-2 |
| MMSE | Mini-Mental State Examination |
| MoCA | Montreal Cognitive Assessment |
| Mono | Monocytes |
| MRI | Magnetic Resonance Imaging |
| MS | Multiple Sclerosis |
| MSA | Multiple System Atrophy |
| MSA-C | Multiple System Atrophy – Cerebellar type |
| MSA-P | Multiple System Atrophy – Parkinsonian type |
| MSD V-PLEX | Meso Scale Discovery V-PLEX |
| N (ATN) | Neurodegeneration |
| NDDs | Neurodegenerative Diseases |
| ND | Non Demented |
| NEU | Neutrophils |
| NfL | Neurofilament Light Chain |

| | |
|---|---|
| NIA-AA | National Institute on Aging and Alzheimer’s Association |
| NINCDS-ARDRA | National Institute of Neurological and Communicative Disorders and Stroke and the Alzheimer's Disease and Related Disorders Association |
| NK cell | Natural killer cel |
| NLR | Neutrophil-to-Lymphocyte Ratio |
| NMO | Neuromyelitis Optica |
| OSAS | Obstructive Sleep Apnea Syndrome |
| PD | Parkinson’s Disease |
| PDD | Parkinson’s Disease Dementia |
| PD-RBD | Parkinson’s Disease with Rapid Eye Movement Sleep Behavior Disorder |
| PET | Positron Emission Tomography |
| PGRN | Progranulin |
| PINK1 | PTEN-Induced Kinase 1 |
| PRISMA | Preferred Reporting Items for Systematic Reviews and Meta-Analyses |
| PRKN | Parkinson Protein (Parkin) |
| PSP | Progressive Supranuclear Palsy |
| RBD | REM Sleep Behavior Disorder |
| ROC | Receiver Operating Characteristic |
| SAA | Serum Amyloid A |
| sAXL | Soluble AXL Receptor Tyrosine Kinase |
| SCD | Subjective Cognitive Decline |
| SIMOA | Single Molecule Array |
| SMA | Spinal Muscular Atrophy |
| sPD | Sporadic Parkinson’s Disease |
| S-PLEX | Meso Scale Discovery S-PLEX |
| sTNF-R2 | Soluble Tumor Necrosis Factor Receptor 2 |
| sTREM-2 | Soluble Triggering Receptor Expressed on Myeloid Cells 2 |
| T (ATN) | Tau |
| TARC | Thymus and Activation-Regulated Chemokine |
| TBI | Traumatic Brain Injury |
| TGF-α | Transforming Growth Factor Alpha |
| TGF-β | Transforming Growth Factor Beta |
| TIMP-1 | Tissue Inhibitor of Metalloproteinases-1 |
| TNF-α | Tumour Necrosis Factor-Alpha |
| TNF-β | Tumor Necrosis Factor beta |
| WBC | White Blood Cell |
| YKL-40 | Chitinase-3-like Protein 1 |
| α1-antichymotrypsin | Alpha-1-Antichymotrypsin |
| α2-macroglobulin | Alpha-2-Macroglobulin |

**Supplementary Material, Table 1**: **Inclusion and Exclusion Criteria**

| **Inclusion criteria:** | **Exclusion criteria** |
|---|---|
| * **Neurodegenerative diseases (NDs):**<br>- Alzheimer's Disease (AD)<br>- Parkinson's Disease (PD)<br>- Huntington's Disease (HD)<br>- Motor Neuron Disease (MND)<br>- Amyotrophic lateral sclerosis (ALS)<br>- Multiple system atrophy (MSA)<br>- Progressive supranuclear palsy (PSP)<br>- Frontotemporal dementia (FTD)<br>- Lewy Body Dementia (LBD) | * **Other diseases**, such as:<br>- MS<br>- (Neuro)inflammatory diseases/infectious/autoimmune diseases<br>- Down's syndrome<br>- Hydrocephalus<br>- Diabetes Mellitus<br>- Cerebrovascular diseases: stroke, CAA, small vessel disease<br>- HIV<br>- Psychiatric diseases<br>- Muscular diseases like SMA, dystrophy, etc<br>- Neuromyelitis optica<br>- Neurotoxicity<br>- Myasthenia Gravis<br>- Prion disease<br>- Narcolepsy<br>- Wilson's disease<br>- Tuberous sclerosis/neurofibromatosis<br>- Polyneuropathy/neuralgia<br>- Tourette's syndrome<br>- Fragile X syndrome<br>- Traumatic brain injury<br>- Ataxia syndroms<br>- REM sleep behaviour disorder<br>- Leukoencephalopathy<br>- Epilepsy<br>- Migraine<br>- Other neurological diseases, rare diseases (like Lafora, Gaucher, Charcot Marot), iatrogenic<br>- Spinal cord diseases<br>- Other organ diseases (ocular, cardiac, etc)<br>- Cancer/fibromas<br>- Drug abuse<br>- After surgery/anesthesia/IC-ventilation<br><br>* Study population with no disease as in inclusion criteria or no risk for it, for example: healthy adults/healthy elderly group, caregivers |
| * **Human** (in vivo) | * **Animal diseases**<br>* **Animal models**<br>* **Cell studies**<br>* **Postmortem** |
| * **Adults** | * Children |
| * **Neuroinflammation biomarkers**:<br>- Wet: blood, CSF, other (saliva, urine, tears)<br>- Imaging: MRI, MRS, PET, etc. specifically looking at neuroinflammation | ***Other type of biomarkers**, like:<br>- DNA<br>- oxidative stress<br>- vascular |

**Supplementary Material, Table 1**: **Inclusion and Exclusion Criteria**

| | |
|---|---|
| | - blood brain barrier<br>- cholesterol and lipids<br>- gut microbiome<br>- synaptic<br>- neurotransmitters<br>- hormones (e.g. insulin, melatonin)<br>- hemostasis<br>- organ specific (ocular, dental, intestinal, skeletal muscle function, liver, etc.)<br>- infections, bacteria<br>- mitochondrial<br>- metals<br>- link with other diseases/symptoms: sleep, OSAS, DM, etc.<br>- circadian rhythm<br>- body temperature<br>- neuropathological<br>- monocyte-specific levels |
| * **Biomarkers of disease progression** | * **Risk factors**, like air pollution, stress, cold, infection, etc |
| * **Observational studies**/trials identifying biomarkers, hypothesis-driven | * **Treatment studies**<br>*Before we look at how treatment effects biomarkers, we need to know which biomarkers are a good presentation of neuroinflammation in NDs. Therefore we are going to exclude studies about treatment influencing biomarkers. Otherwise it would also be an evaluation of treatment options. These treatment studies are: clinical trials, medication, diet, therapy, exercise.*<br><br>* Large proteomic exploratory research with no hypothesis.<br>*These will be excluded as they are hard to compare with hypothesis-driven research.*<br><br>* **Other types of articles**: Editorials, Letter to the Editor, poster abstracts, short communications<br><br>* Articles explaining the mechanism of neuroinflammation, the mechanism of a lab tool/test/scan/marker, etc. |
| * Articles in **English** | * No English full text |

**Supplementary Table 2: Complete AD Results in different studies**

| **Biomarker**<br>(number of studies) | **Number of studies by biofluid type** | | | | | |
|---|---|---|---|---|---|---|
| **CCL-23 (4):**<br>blood (4), CSF (1) | Ns.<br>Blood | Ns.<br>CSF | ↑<br>Blood | ↓<br>Blood | ↑<br>CSF | ↓<br>CSF |
| AD vs. control | **2** [1a] | | | | | |
| MCI vs. control | **2** [1a] | | | | **1** [1a] | |
| **Complement 1q (6):**<br>blood (2), CSF (5) | Ns.<br>Blood | Ns.<br>CSF | ↑<br>Blood | ↓<br>Blood | ↑<br>CSF | ↓<br>CSF |
| AD vs. control | **2** [2,3] | **1** [4] | **1** [5c] | | | |
| AD vs. MCI | **2** [2,3] | **1** [4] | | | | |
| MCI vs. control | **2** [2,3] | **2** [4,6b] | **1** [5c] | | | |
| **Complement 3 (12):**<br>blood (7), CSF (6) | Ns.<br>Blood | Ns.<br>CSF | ↑<br>Blood | ↓<br>Blood | ↑<br>CSF | ↓<br>CSF |
| AD vs. control | **3** [2,3,7] | **2** [8,9] | **3** [7,10,11] | **1** [12] | **2** [5,13] | |
| AD vs. MCI | **2** [3,7] | **3** [5,8,9] | | **1** [2] | | |
| MCI vs. control | **2** [3,7] | **4** [5,8,9,13] | **2** [2,7] | | | |
| **Complement 4 (6):**<br>blood (3), CSF (3) | Ns.<br>Blood | Ns.<br>CSF | ↑<br>Blood | ↓<br>Blood | ↑<br>CSF | ↓<br>CSF |
| AD vs. control | **2** [3,12,5c] | | **1** [2] | | **1** [5b] | |
| AD vs. MCI | **2** [2,3] | **1** [5] | | | | |
| MCI vs. control | **2** [2,3] | **2** [5,6] | | | | |
| **CRP/hs-CRP (41/14):**<br>blood (34/14), CSF (9/0) | Ns.<br>Blood | Ns.<br>CSF | ↑<br>Blood | ↓<br>Blood | ↑<br>CSF | ↓<br>CSF |
| AD vs. control | **11** [14-24] | **3** [19,25,4c] | **CRP: 5** [26-30]<br>**hs-CRP: 3** [31-33] | **CRP: 3** [2,12,34b] | | |
| AD vs. MCI | **6** [19-21,23,24,35] | **2** [19,4c] | **CRP: 1** [36]<br>**hs-CRP: 1** [33] | **CRP: 3** [2,37b,34b] | | |
| MCI vs. control | **12** [17,19-21,23,24,33,34,38,39,40b,3c] | **4** [5,19,38,4c] | **CRP: 1** [37b] | **CRP: 1** [38] | | |
| **Eotaxin-1 (20):**<br>blood (15), CSF (9) | Ns.<br>Blood | Ns.<br>CSF | ↑<br>Blood | ↓<br>Blood | ↑<br>CSF | ↓<br>CSF |
| Below detection limit | | **4** [4,37,41,42] | | | | |
| AD vs. control | **4** [21,43,44,45b] | **1** [46] | **2** [2,46] | | **1** [47] | |
| AD vs. MCI | **2** [21,44] | **1** [46] | **2** [2,43] | | | |
| MCI vs. control | **4** [2,21,43,44] | **1** [46] | **1** [40d] | | | |
| **Eotaxin-3 (9):**<br>blood (5), CSF (5) | Ns.<br>Blood | Ns.<br>CSF | ↑<br>Blood | ↓<br>Blood | ↑<br>CSF | ↓<br>CSF |
| Below detection limit | **1** [48] | **4** [4,37,41,42] | | | | |
| AD vs. control | **2** [2,21] | | **1** [49] | | | |
| AD vs. MCI | **2** [2,21] | | | | | |
| MCI vs. control | **2** [2,21] | | | | | |
| **ESR (4):**<br>blood (4) | Ns.<br>Blood | | ↑<br>Blood | | ↓<br>Blood | |
| AD vs. control | **1** [16] | | **2** [17,28] | | | |
| Factor H (7):<br>blood (4), CSF (3) | Ns.<br>Blood | Ns.<br>CSF | ↑<br>Blood | ↓<br>Blood | ↑<br>CSF | ↓<br>CSF |
| AD vs. control | **4** [2,3,7,50] | **1** [8] | | | | |
| AD vs. MCI | **2** [2,3,7] | **1** [8] | | | | |
| MCI vs. control | **1** [3,7] | **2** [6,8] | **2** [2,7] | | | |
| **Fractalkine (4):**<br>blood (3), CSF (2) | Ns.<br>Blood | Ns.<br>CSF | ↑<br>Blood | ↓<br>Blood | ↑<br>CSF | ↓<br>CSF |
| AD vs. control | **2** [51,45b] | **1** [52] | **1** [53] | | **1** [53] | |
| AD vs. MCI | | **1** [52] | | **1** [53] | | **1** [53] |
| MCI vs. control | | **1** [52] | **1** [53] | | **1** [53] | |
| **G-CSF (9):**<br>blood (8), CSF (3) | Ns.<br>Blood | Ns.<br>CSF | ↑<br>Blood | ↓<br>Blood | ↑<br>CSF | ↓<br>CSF |
| AD vs. control | **4** [43,44,46,54] | **1** [46] | | **1** [45a] | **1** [47] | |
| AD vs. MCI | **4** [43,44,46,54] | **1** [46] | | | | |
| MCI vs. control | **4** [43,44,46,54] | **1** [46] | | | | |
| **GFAP (16):**<br>blood (10), CSF (7) | Ns.<br>Blood | Ns.<br>CSF | ↑<br>Blood | ↓<br>Blood | ↑<br>CSF | ↓<br>CSF |

| | | | | | | |
|---|---|---|---|---|---|---|
| AD vs. control | | 1 [41] | 6 [21,55-59] | | 2 [55,60] | |
| AD vs. MCI | | | 2 [56,58] | | 2 [61e,60e] | |
| MCI vs. control | 1 [58] | 1 [38] | 3 [21,38,55] | | 3 [55,61e,60f] | |
| **GM-CSF (10):** blood (8), CSF (4) | Ns. Blood | Ns. CSF | ↑ Blood | ↓ Blood | ↑ CSF | ↓ CSF |
| Below detection limit | | 1 [4] | | | | |
| AD vs. control | 3 [2,43,44,62] | 1 [47] | | 1 [46] | | 1 [46] |
| AD vs. MCI | 2 [2,43,44] | | | | | |
| MCI vs. control | 2 [2,43,44] | | | 2 [46,63b] | | 1 [46] |
| **ICAM-1/sICAM-1 (9/7):** blood (5/4), CSF (5/4) | Ns. Blood | Ns. CSF | ↑ Blood | ↓ Blood | ↑ CSF | ↓ CSF |
| AD vs. control | 1 [21] | 7 [4,5,42,64-66,37b] | **ICAM-1:** 1 [67] **sICAM-1:** 1 [68] | | | |
| AD vs. MCI | 2 [21,68c] | 2 [4,64] | | **ICAM-1:** 1 [37b] | | |
| MCI vs. control | 2 [21,68] | 3 [4,64,37b] | | | | |
| **IFN-y (29):** blood (24), CSF (8) | Ns. Blood | Ns. CSF | ↑ Blood | ↓ Blood | ↑ CSF | ↓ CSF |
| Below detection limit | 2 [35,69] | 3 [4,37,70] | | | | |
| AD vs. control | 10 [23,43-46,62,71-74b] | 3 [42,47,72] | 3 [75-77] | | 1 [46] | |
| AD vs. MCI | 6 [23,43,44,46,73,74] | | 1 [77] | | | |
| MCI vs. control | 7 [23,43,44,46,73,74,78] | | 1 [77] | | 1 [46] | |
| **IL-1α (21):** blood (17), CSF (7) | Ns. Blood | Ns. CSF | ↑ Blood | ↓ Blood | ↑ CSF | ↓ CSF |
| Below detection limit | 1 [49] | 5 [4,37,38,42,70] | | | | |
| AD vs. control | 5 [2,62,74,75,79] | | 3 [44,71,80] | 2 [45,81b] | | |
| AD vs. MCI | 4 [2,44,74,80] | | | 1 [81] | | |
| MCI vs. control | 4 [2,74,80,81] | | 1 [44] | | | |
| **IL-1β (50):** blood (43), CSF (11) | Ns. Blood | Ns. CSF | ↑ Blood | ↓ Blood | ↑ CSF | ↓ CSF |
| Below detection limit | 3 [35,49,82] | 5 [4,37,38,42,70] | | | | |
| AD vs. control | 15 [2,23,43,44,57,62,72,74,75,81,83-86,45b] | 3 [46,47,72] | 12 [46,54,69,71,76,80,87-91,81g] | | 2 [90,92] | |
| AD vs. MCI | 8 [2,43,44,54,74,85,86,90] | 2 [46,90] | 4 [36,80,89,81g] | 2 [23,84] | | |
| MCI vs. control | 7 [2,38,43,44,74,85,86] | 1 [46] | 11 [38,46,54,84,88-91,93,94,95c] | 1 [63b] | 1 [90] | |
| **IL-1Ra (14):** blood (11), CSF (3) | Ns. Blood | Ns. CSF | ↑ Blood | ↓ Blood | ↑ CSF | ↓ CSF |
| Below detection limit | | 1 [38] | | | | |
| AD vs. control | 5 [43,44,46,69,80] | 1 [46] | 2 [80,85] | | 2 [42,47] | |
| AD vs. MCI | 3 [43,44,46] | 1 [46] | 1 [35] | | | |
| MCI vs. control | 5 [38,43,44,46,80] | 1 [46] | 1 [38] | | | |
| **IL-2 (24):** blood (18), CSF (8) | Ns. Blood | Ns. CSF | ↑ Blood | ↓ Blood | ↑ CSF | ↓ CSF |
| Below detection limit | 1 [63] | 4 [4,37,42,70] | | | | |
| AD vs. control | 9 [2,23,43-45,62,69,72,75b] | 3 [46,47,72] | | 2 [46,71] | | |
| AD vs. MCI | 3 [2,43,44] | 1 [46] | | 1 [23] | | |
| MCI vs. control | 3 [2,43,44] | 1 [46] | 1 [95c] | 1 [46] | | |
| **IL-3 (6):** blood (6), CSF (2) | Ns. Blood | Ns. CSF | ↑ Blood | ↓ Blood | ↑ CSF | ↓ CSF |
| AD vs. control | 2 [79,96] | 1 [96] | 1 [45b] | | | |
| **IL-4 (24):** blood (19), CSF (8) | Ns. Blood | Ns. CSF | ↑ Blood | ↓ Blood | ↑ CSF | ↓ CSF |
| Below detection limit | 1 [69] | 4 [4,37,42,70] | | | | |
| AD vs. control | 6 [2,23,43,62,72,74] | 1 [72] | 3 [46,71,87] | | 2 [46,47] | |
| AD vs. MCI | 3 [2,43,74] | | | 1 [23] | | |
| MCI vs. control | 3 [2,43,74] | | 2 [46,95c] | | 1 [46] | |
| **IL-5 (17):** blood (13), CSF (7) | Ns. Blood | Ns. CSF | ↑ Blood | ↓ Blood | ↑ CSF | ↓ CSF |
| Below detection limit | 1 [63] | 2 [4,42] | | | | |
| AD vs. control | 6 [2,43,44,49,62,74] | 2 [37,47] | 2 [45,46b] | | 1 [46] | |

| | | | | | | |
|---|---|---|---|---|---|---|
| AD vs. MCI | **5** [2,43,44,49,74] | **1** [37] | | | | |
| MCI vs. control | **5** [2,43,44,49,74] | **1** [37] | **1** [46] | | **1** [46] | |
| **IL-6 (83):**<br>blood (67), CSF (21) | Ns.<br>Blood | Ns.<br>CSF | ↑<br>Blood | ↓<br>Blood | ↑<br>CSF | ↓<br>CSF |
| Below detection limit | **1** [82] | | | | | |
| AD vs. control | **26** [2,3,23,24,42-44,49,57,58,62,69,72,73,75,81,83,84,86,90,97-101,102b] | **8** [4,37,61,64,66,70,72,90] | **12** [15,28,29,31,51,71,74,76,87,91,103,81g] | **1** [46] | **2** [47,92] | **1** [46] |
| AD vs. MCI | **18** [2,3,23,24,43,44,49,54,58,73,81,84,86,90,98-101] | **7** [4,37,61,64,66,70,90] | **5** [35,36,54,74,97] | | | |
| MCI vs. control | **16** [2,3,23,24,43,44,49,58,73,81,84,90,98-101] | **9** [4,37,38,61,64,66,70,90,104] | **5** [54,78,86,91,93] | **3** [38,46,63b] | | **1** [46] |
| **IL-7 (19):**<br>blood (14), CSF (9) | Ns.<br>Blood | Ns.<br>CSF | ↑<br>Blood | ↓<br>Blood | ↑<br>CSF | ↓<br>CSF |
| Below detection limit | | **2** [37,42] | | | | |
| AD vs. control | **9** [2,43,44,46,49,74,105,45b] | **2** [64,66] | | | **2** [46,47] | |
| AD vs. MCI | **6** [2,43,44,46,49,74] | **2** [64,66] | | | | |
| MCI vs. control | **6** [2,43,44,46,49,74] | **3** [64,66,104] | | **1** [63] | **1** [46] | |
| **IL-8 (43):**<br>blood (33), CSF (15) | Ns.<br>Blood | Ns.<br>CSF | ↑<br>Blood | ↓<br>Blood | ↑<br>CSF | ↓<br>CSF |
| AD vs. control | **10** [2,23,43-46,58,62,73,76b] | **9** [4,37,42,46,64,70,72,106,41b] | **4** [54,75,100,107] | **3** [71,72,108] | **1** [47] | |
| AD vs. MCI | **9** [2,23,43,44,46,54,58,73,108] | **5** [4,37,46,64,70] | | | | |
| MCI vs. control | **8** [2,23,38,43,44,46,54,73] | **5** [4,37,46,64,70] | **2** [38,100] | **2** [58,108] | **2** [38,104] | |
| **IL-9 (6):**<br>blood (3), CSF (4) | Ns.<br>Blood | Ns.<br>CSF | ↑<br>Blood | ↓<br>Blood | ↑<br>CSF | ↓<br>CSF |
| AD vs. control | **2** [43,46] | **1** [66] | **2** [46,47] | | | |
| AD vs. MCI | **2** [43,46] | **1** [66] | | | | |
| MCI vs. control | **2** [43,46] | **1** [66] | **1** [46] | | | |
| **IL-10 (42):**<br>blood (36), CSF (11) | Ns.<br>Blood | Ns.<br>CSF | ↑<br>Blood | ↓<br>Blood | ↑<br>CSF | ↓<br>CSF |
| Below detection limit | **1** [35] | **4** [4,37,38,70] | | | | |
| AD vs. control | **17** [2,23,43-46,49,57,58,62,72,73,84,90,99,102,108b] | **3** [46,66,90] | **2** [69,71] | **6** [23,42,54,62,84,90] | **2** [47,72] | |
| AD vs. MCI | **11** [2,36,43,44,46,49,73,84,90,99,108] | **3** [46,66,90] | **2** [58,74] | **2** [54,62] | | |
| MCI vs. control | **12** [2,43,44,46,49,54,73,78,84,90,99,108] | **3** [46,66,90] | **2** [38,95c] | **4** [38,58,84,90] | | |
| **IL-12(p70) (24):**<br>blood (20), CSF (6) | Ns.<br>Blood | Ns.<br>CSF | ↑<br>Blood | ↓<br>Blood | ↑<br>CSF | ↓<br>CSF |
| Below detection limit | **2** [23,109] | **2** [4,109] | | | | |
| AD vs. control | **10** [2,43,44,46,62,72,79,99,45b,110h] | **3** [46,47,72] | **IL-12: 2** [76,110i]<br>**IL12p70: 1** [71] | | | **IL-12p70: 1** [42] |
| AD vs. MCI | **5** [2,43,44,46,99] | **1** [46] | | | | |
| MCI vs. control | **6** [2,43,44,46,94,99] | **1** [46] | | **IL-12p70**: **1** [63b] | | |
| **IL-12/IL-23p40 (7):**<br>blood (4), CSF (4) | Ns.<br>Blood | Ns.<br>CSF | ↑<br>Blood | ↓<br>Blood | ↑<br>CSF | ↓<br>CSF |
| Below detection limit | | **1** [4] | | | | |
| AD vs. control | **3** [2,49,111] | **2** [37,64] | | | | **1** [111b,c] |
| AD vs. MCI | **3** [2,49,111] | **1** [111] | | | | |
| MCI vs. control | **3** [2,49,111] | **1** [64] | | | | **1** [111b,c] |
| **IL-12p40 (5):**<br>blood (3), CSF (3) | Ns.<br>Blood | Ns.<br>CSF | ↑<br>Blood | ↓<br>Blood | ↑<br>CSF | ↓<br>CSF |
| Below detection limit | **1** [35] | | | | | |
| AD vs. control | | **1** [66] | **1** [45b] | | | |
| AD vs. MCI | | **1** [66] | | | | |
| MCI vs. control | | **1** [66] | | | | |
| **IL-13 (19):**<br>blood (15), CSF (5) | Ns.<br>Blood | Ns.<br>CSF | ↑<br>Blood | ↓<br>Blood | ↑<br>CSF | ↓<br>CSF |
| Below detection limit | **2** [23,49] | **3** [4,37,42] | | | | |
| AD vs. control | **4** [2,43,44,62] | **1** [47] | **3** [45,46,72b] | **1** [76] | **2** [46,72] | |
| AD vs. MCI | **3** [2,43,44] | | | | | |
| MCI vs. control | **3** [2,43,44] | | **1** [46] | **1** [63b] | **1** [46] | |

| | | | | | | |
|---|---|---|---|---|---|---|
| **IL-15 (17):** blood (11), CSF (8) | Ns. Blood | Ns. CSF | ↑ Blood | ↓ Blood | ↑ CSF | ↓ CSF |
| Below detection limit | **1** [63] | **1** [4] | | | | |
| AD vs. control | **4** [2,43,44,49] | **3** [46,64,37b] | **1** [45b] | **1** [46] | **2** [42,47] | |
| AD vs. MCI | **4** [2,43,44,49] | **3** [46,64,37b] | | | | |
| MCI vs. control | **5** [2,43,44,49,104] | **3** [46,64,37b] | | **1** [46] | | |
| **IL-16 (9):** blood (4), CSF (5) | Ns. Blood | Ns. CSF | ↑ Blood | ↓ Blood | ↑ CSF | ↓ CSF |
| Below detection limit | | **2** [4,42] | | | | |
| AD vs. control | **3** [2,79,110j] | **2** [64,37b] | **1** [110i] | | | |
| AD vs. MCI | **1** [2] | **2** [64,37b] | | | | |
| MCI vs. control | **1** [2] | **3** [64,104,37b] | | | | |
| **IL-17 (11):** blood (11) | Ns. Blood | | ↑ Blood | | ↓ Blood | |
| Below detection limit | **2** [48,63] | | | | | |
| AD vs. control | **5** [2,43,44,49,74] | | **2** [54,69] | | | |
| AD vs. MCI | **5** [2,43,44,49,74] | | **1** [54] | | | |
| MCI vs. control | **6** [2,43,44,49,54,74] | | | | | |
| **IL-17A (9):** blood (5), CSF (6) | Ns. Blood | Ns. CSF | ↑ Blood | ↓ Blood | ↑ CSF | ↓ CSF |
| Below detection limit | **1** [35] | **3** [4,37,42] | | | | |
| AD vs. control | **1** [57] | **1** [47] | **1** [46] | | | **1** [46] |
| MCI vs. control | | | **1** [46] | | | **1** [46] |
| **IL-18 (17):** blood (16), CSF (3) | Ns. Blood | Ns. CSF | ↑ Blood | ↓ Blood | ↑ CSF | ↓ CSF |
| AD vs. control | **5** [3,51,79,80,110h] | | **7** [54,67,89,91,112, 113,110i] | | | |
| AD vs. MCI | **4** [3,35,54,80] | | | **2** [80,89] | | |
| MCI vs. control | **3** [3,38,80] | **1** [38] | **4** [54,80,89,91] | | | |
| **IL-33 (3):** blood (3), CSF (1) | Ns. Blood | Ns. CSF | ↑ Blood | ↓ Blood | ↑ CSF | ↓ CSF |
| Below detection limit | **1** [35] | | | | | |
| AD vs. control | | | **1** [80] | **1** [90] | | **1** [90] |
| AD vs. MCI | **1** [90] | **1** [90] | **1** [80] | | | |
| MCI vs. control | | | | **1** [90] | | **1** [90] |
| **IP-10 (28):** blood (18), CSF (13) | Ns. Blood | Ns. CSF | ↑ Blood | ↓ Blood | ↑ CSF | ↓ CSF |
| AD vs. control | **7** [2,3,21,43,44,84,57b] | **7** [4,5,41,46,47,66,106] | **3** [45,71,76b] | **2** [46,57] | | **1** [42] |
| AD vs. MCI | **6** [2,3,21,43,44,84] | **4** [4,5,46,66] | | | | **1** [37] |
| MCI vs. control | **7** [2,3,21,38,43,44,84] | **5** [4,5,46,66,104] | **1** [38] | **1** [46] | **2** [37,38] | |
| **MCP-1 (49):** blood (34), CSF (23) | Ns. Blood | Ns. CSF | ↑ Blood | ↓ Blood | ↑ CSF | ↓ CSF |
| AD vs. control | **10** [2,3,21,43,44,49,53,54,96,108] | **12** [4,5,37,42,47,53,64,65,70,96,114,41b] | **4** [71,75,76,115] | **5** [46,62,67,113, 45b] | **2** [52,106] | **1** [46] |
| AD vs. MCI | **9** [3,21,35,43,44,49,53,54,108] | **7** [4,5,37,52,53,64,70] | **1** [115b] | **2** [2,44] | | |
| MCI vs. control | **7** [3,21,40,43,49,54,108b] | **6** [4,5,38,53,64,70] | **4** [2,38,44,53] | **3** [38,46,63] | **2** [52,104] | **1** [46] |
| **MCP-4 (9):** blood (5), CSF (6) | Ns. Blood | Ns. CSF | ↑ Blood | ↓ Blood | ↑ CSF | ↓ CSF |
| Below detection limit | | **4** [4,37,41,42] | | | | |
| AD vs. control | **2** [2,21] | | | | | |
| AD vs. MCI | **2** [2,21] | | | | | |
| MCI vs. control | **2** [2,21] | | | | | |
| **MDC (9):** blood (4), CSF (6) | Ns. Blood | Ns. CSF | ↑ Blood | ↓ Blood | ↑ CSF | ↓ CSF |
| Below detection limit | | **4** [4,37,41,42] | | | | |
| AD vs. control | **2** [2,21] | **1** [64] | | | | |
| AD vs. MCI | **2** [2,21] | **1** [64] | | | | |
| MCI vs. control | **2** [2,21] | **1** [64] | | | | |
| **MIF (10):** blood (4), CSF (7) | Ns. Blood | Ns. CSF | ↑ Blood | ↓ Blood | ↑ CSF | ↓ CSF |
| AD vs. control | **5** [2,3,79,116,5c] | **1** [4] | | | **4** [116-118,5b] | |
| AD vs. MCI | **2** [2,3] | **2** [4,118] | | | **1** [117] | |

| | | | | | | |
|---|---|---|---|---|---|---|
| MCI vs. control | **3** [2,3,5c] | **3** [4,117,6b] | | | **2** [118,5b] | |
| **MIP-1α (16):**<br>blood (12), CSF (8) | Ns.<br>Blood | Ns.<br>CSF | ↑<br>Blood | ↓<br>Blood | ↑<br>CSF | ↓<br>CSF |
| Below detection limit | **2** [43,49] | **3** [4,37,42] | | | | |
| AD vs. control | **3** [2,21,96] | **3** [46,47,96] | **2** [46,76] | **1** [62] | | |
| AD vs. MCI | **2** [2,21] | **1** [46] | **1** [46] | | | |
| MCI vs. control | **2** [2,21] | **1** [46] | | | | |
| **MIP-1β (20):**<br>blood (13), CSF (11) | Ns.<br>Blood | Ns.<br>CSF | ↑<br>Blood | ↓<br>Blood | ↑<br>CSF | ↓<br>CSF |
| Below detection limit | **1** [46] | **1** [46] | | | | |
| AD vs. control | **4** [2,21,44,96] | **7** [4,5,37,42,47,64,96] | | **2** [45,62b] | | |
| AD vs. MCI | **3** [21,35,44] | **5** [4,5,37,47,64] | **1** [2] | | | |
| MCI vs. control | **2** [21,44] | **5** [4,5,37,47,64] | | **1** [2] | | |
| **MMP-1 (3):**<br>blood (2), CSF (2) | Ns.<br>Blood | Ns.<br>CSF | ↑<br>Blood | ↓<br>Blood | ↑<br>CSF | ↓<br>CSF |
| AD vs. control | **1** [73] | **1** [119] | | **1** [72] | **1** [72] | |
| AD vs. MCI | **1** [73] | | | | | |
| MCI vs. control | **1** [73] | | | | | |
| **MMP-2 (5):**<br>blood (3), CSF (3) | Ns.<br>Blood | Ns.<br>CSF | ↑<br>Blood | ↓<br>Blood | ↑<br>CSF | ↓<br>CSF |
| AD vs. control | **1** [49] | **2** [64,72] | **1** [72] | | | |
| AD vs. MCI | **1** [49] | **1** [64] | | | | |
| MCI vs. control | **1** [49] | **1** [64] | | | | |
| **MMP-9 (7):**<br>blood (6), CSF (2) | Ns.<br>Blood | Ns.<br>CSF | ↑<br>Blood | ↓<br>Blood | ↑<br>CSF | ↓<br>CSF |
| AD vs. control | **2** [72,73] | **1** [72] | **1** [120] | **2** [54,62] | | |
| AD vs. MCI | **2** [54,73] | | **1** [120] | | | |
| MCI vs. control | **1** [73] | | **1** [120] | **1** [54] | | |
| **MMP-10 (3):**<br>blood (1), CSF (3) | Ns.<br>Blood | Ns.<br>CSF | ↑<br>Blood | ↓<br>Blood | ↑<br>CSF | ↓<br>CSF |
| AD vs. control | **1** [72] | | | | 3 [64,72,119] | |
| **Osteopontin (4):**<br>blood (3), CSF (3) | Ns.<br>Blood | Ns.<br>CSF | ↑<br>Blood | ↓<br>Blood | ↑<br>CSF | ↓<br>CSF |
| AD vs. control | **2** [121,122k] | | **2** [121,122l] | | **1** [122] | |
| MCI vs. control | **2** [122,38m] | | | **1** [38n] | **1** [38n] | |
| **RANTES (14):**<br>blood (13), CSF (4) | Ns.<br>Blood | Ns.<br>CSF | ↑<br>Blood | ↓<br>Blood | ↑<br>CSF | ↓<br>CSF |
| Below detection limit | **2** [43,48] | | | | | |
| AD vs. control | **1** [96] | **3** [46,47,96] | **3** [15,46,67] | **1** [62] | | |
| AD vs. MCI | | **1** [46] | | | | |
| MCI vs. control | | **1** [46] | **1** [46] | | | |
| **SAA (8):**<br>blood (5), CSF (3) | Ns.<br>Blood | Ns.<br>CSF | ↑<br>Blood | ↓<br>Blood | ↑<br>CSF | ↓<br>CSF |
| AD vs. control | **1** [21] | **2** [4,37] | | | **1** [42] | |
| AD vs. MCI | **1** [21] | **2** [4,37] | | | | |
| MCI vs. control | **1** [21] | **2** [4,37] | **1** [94] | | | |
| **sAXL (4):**<br>blood (1), CSF (1) | Ns.<br>Blood | Ns.<br>CSF | ↑<br>Blood | ↓<br>Blood | ↑<br>CSF | ↓<br>CSF |
| AD vs. control | | **1** [5] | **1** [3] | | | |
| AD vs. MCI | **1** [3] | **1** [5] | | | | |
| MCI vs. control | **1** [3] | **2** [5,6] | | | | |
| **sTNF-R1 (7):**<br>blood (5), CSF (3) | Ns.<br>Blood | Ns.<br>CSF | ↑<br>Blood | ↓<br>Blood | ↑<br>CSF | ↓<br>CSF |
| AD vs. control | **1** [96] | **2** [5,66] | **2** [68,123] | | **1** [96] | |
| AD vs. MCI | | **2** [5,66] | **1** [68] | | | |
| MCI vs. control | **1** [68] | **2** [5,66] | | | | |
| **sTNF-R2 (6):**<br>blood (3), CSF (4) | Ns.<br>Blood | Ns.<br>CSF | ↑<br>Blood | ↓<br>Blood | ↑<br>CSF | ↓<br>CSF |
| AD vs. control | **1** [68] | **2** [5,66] | | | | |
| AD vs. MCI | **1** [68] | **3** [5,66,124] | | | | |
| MCI vs. control | **1** [68] | **2** [5,66] | | | | |
| **sTREM-2 (22):**<br>blood (6), CSF (18) | Ns.<br>Blood | Ns.<br>CSF | ↑<br>Blood | ↓<br>Blood | ↑<br>CSF | ↓<br>CSF |

| | | | | | | |
|---|---|---|---|---|---|---|
| AD vs. control | **3** [3,20,59] | **5** [66,118,125-127] | **1** [128] | | **7** [14,20,52,129, 61e,4b,5b,c] | |
| AD vs. MCI | **2** [3,20] | **5** [4,66,124,126,127] | | | **1** [52] | **1** [118] |
| MCI vs. control | **2** [3,20] | **5** [6,38,66,126,127] | | **1** [38] | **5** [20,52,118,129, 4b] | |
| **TARC (9):** blood (5), CSF (4) | Ns. Blood | Ns. CSF | ↑ Blood | ↓ Blood | ↑ CSF | ↓ CSF |
| Below detection limit | | **2** [4,41] | | | | |
| AD vs. control | **2** [21,49] | **1** [42] | | | | |
| AD vs. MCI | **2** [21,49] | | | | | **1** [37b] |
| MCI vs. control | **2** [21,49] | **1** [37b] | | | | |
| **TGF-β1 (8):** blood (6), CSF (3) | Ns. Blood | Ns. CSF | ↑ Blood | ↓ Blood | ↑ CSF | ↓ CSF |
| Below detection limit | **1** [96] | | | | | |
| AD vs. control | **1** [110f] | **2** [5,66] | **2** [112,110i] | **2** [113,130] | | |
| AD vs. MCI | | **2** [5,66] | | | | |
| MCI vs. control | | **2** [5,66] | | | | |
| **TIMP-1 (5):** blood (5), CSF (3) | Ns. Blood | Ns. CSF | ↑ Blood | ↓ Blood | ↑ CSF | ↓ CSF |
| AD vs. control | **1** [96] | | | **1** [54] | **1** [96] | |
| MCI vs. control | **1** [38n] | **1** [38n] | **1** [38m] | **1** [54] | | |
| **TNF-α (63):** blood (53), CSF (14) | Ns. Blood | Ns. CSF | ↑ Blood | ↓ Blood | ↑ CSF | ↓ CSF |
| Below detection limit | **1** [82] | **5** [4,37,38,42,70] | | | | |
| AD vs. control | **20** [2,22-24,43,44,49,57,58,62,69, 73-76,83,84,86,99,102] | **4** [46,66,72,131] | **11** [15,46,54,71,77,87, 91,97,103,132,133] | **3** [45,72,108b] | **2** [47,92] | |
| AD vs. MCI | **13** [2,24,35,43,44,49,58,73,74, 84,86,97,99] | **2** [46,66] | **4** [23,36,77,133] | | | |
| MCI vs. control | **14** [2,24,43,44,49,58,73,74,78, 84,86,97,99,133] | **2** [46,66] | **7** [38,46,54,77,91,93,94] | **1** [95c] | | |
| **TNF-β (8):** blood (5), CSF (5) | Ns. Blood | Ns. CSF | ↑ Blood | ↓ Blood | ↑ CSF | ↓ CSF |
| Below detection limit | | **3** [4,37,42] | | | | |
| AD vs. control | **3** [2,44,79] | | | | | |
| AD vs. MCI | **2** [2,44] | | | | | |
| MCI vs. control | **2** [2,44] | | | | | |
| **<u>WBC</u>: NLR (10):** blood (10); **LEUK (9):** blood (8), CSF (1); **NEU (4):** blood (4); **LYM (4):** blood (4) | Ns. Blood | Ns. CSF | ↑ Blood | ↓ Blood | ↑ CSF | ↓ CSF |
| AD vs. control | **NLR: 1** [134]; **LEUK: 3** [17,20,135]; **NEU: 4** [134-136,137o]; **LYM: 4** [134-137] | **LEUK: 1** [138] | **NLR: 5** [16,135,136,139,137o] **NEU: 1** [137o] | | | |
| AD vs. MCI | **NLR: 1** [136]; **LEUK: 2** [135,140]; **NEU: 3** [135-137]; **LYM: 3** [135-137] | | **LEUK: 1** [20] | | **LEUK: 1** [138] | |
| MCI vs. control | **NLR: 1** [137b,o]; **LEUK: 1** [135]; **NEU: 3** [135,136,137b,o]; **LYM: 3** [135-137] | **LEUK: 1** [138] | **NLR: 3** [135,136,139]; **LEUK: 1** [20] | | | |
| **YKL-40 (41):** blood (11), CSF (36) | Ns. Blood | Ns. CSF | ↑ Blood | ↓ Blood | ↑ CSF | ↓ CSF |
| AD vs. control | **4** [3,19,53,59] | **2** [37,53] | **1** [141] | | **19** [42,52,55,114,1 25,141-149, 64p,41b,19b, 150b,151c] | |
| AD vs. MCI | **3** [3,19,53] | **8** [19,37,70,143-146,151c] | | | **3** [52,144,148] | **1** [152] |
| MCI vs. control | **5** [3,19,38,53,5c] | **6** [19,37,52,55,145,6b] | **2** [141,40p] | | **13** [38,53,141,143, 146-149, 152,153, 64q,5b,151c] | |
| **α1-antichymotrypsin (6):** blood (4), CSF (3) | Ns. Blood | Ns. CSF | ↑ Blood | ↓ Blood | ↑ CSF | ↓ CSF |

| | | | | | | |
|---|---|---|---|---|---|---|
| AD vs. control | | | | **1** [10] | **2** [13,65] | |
| MCI vs. control | | | | **1** [10] | **1** [13] | |
| **α2-macroglobuline (4):** blood (4), CSF (1) | Ns. Blood | Ns. CSF | ↑ Blood | ↓ Blood | ↑ CSF | ↓ CSF |
| AD vs. control | **2** [7,81] | | **1** [154] | | | |
| AD vs. MCI | **3** [7,81,154] | | | | | |
| MCI vs. control | **1** [81] | | **2** [7,154] | | | |

**Complete list of biomarkers investigated in AD, ordered alphabetically.** Biomarkers and total number of studies in bold.

[a] = One article with 4 different cohorts; [b] = Corrected for covariates; [c] = Corrected for multiple testing; [d] = In African-American subgroup, not in Caucasian and Hispanic subgroup; [e] = only compared with control group without AD biomarkers; [f] = only compared with MCI group with AD biomarkers; [g] = Only in EOAD, not in LOAD; [h] = Severe AD; [i] = Mild and moderate AD; [j] = Severe AD; [k] = AD disease duration > 2 years; [l] = AD disease duration =< 2 years; [m] = healthy controls; [n] = cognitively normal participants; [o] = at certain timepoints. [p] = Only in Hispanic subgroup; [q] = AD & MCI higher than HC, but not higher than CN.

All abbreviations appearing in this table are defined in the abbreviation list

**Supplementary table 3: Complete PD results in different studies**

| **Biomarker** (number of studies) | **Number of studies by biofluid type** | | | | | |
|---|---|---|---|---|---|---|
| **Complement 3 (5):** Blood (4), CSF (1) | Ns. Blood | Ns. CSF | ↑ Blood | ↓ Blood | ↑ CSF | ↓ CSF |
| PD vs. control | | **1** [155] | | **2** [156,157] | | |
| **Complement 4 (3):** Blood (3) | Ns. Blood | | ↑ Blood | ↓ Blood | | |
| PD vs. control | **1** [42] | | | | | |
| **CRP/hs-CRP (27/10):** Blood (27/6), CSF (9/0) | Ns. Blood | Ns. CSF | ↑ Blood | ↓ Blood | ↑ CSF | ↓ CSF |
| PD vs. control | **15** [157-170,171a] | **5** [159,160,163,172,173] | **CRP: 5** [174-176, 164b,177c]; **hs-CRP: 5** [178-181,182d] | **CRP: 1** [173] | | |
| **Eotaxin-1 (5):** Blood (3), CSF (2) | Ns. Blood | Ns. CSF | ↑ Blood | ↓ Blood | ↑ CSF | ↓ CSF |
| PD vs. control | **1** [183] | **1** [184] | | | | |
| **Fractalkine (4):** Blood (3), CSF (2) | Ns. Blood | Ns. CSF | ↑ Blood | ↓ Blood | ↑ CSF | ↓ CSF |
| PD vs. control | **1** [160] | **2** [160,184] | **2** [185,186] | | | |
| **GM-CSF (6):** Blood (4), CSF (4) | Ns. Blood | Ns. CSF | ↑ Blood | ↓ Blood | ↑ CSF | ↓ CSF |
| Below detection limit | **1** [187] | **3** [184,187,188] | | | | |
| PD vs. control | **1** [160] | **1** [160] | | | | |
| **ICAM-1 (3):** Blood (2), CSF (2) | Ns. Blood | Ns. CSF | ↑ Blood | ↓ Blood | ↑ CSF | ↓ CSF |
| PD vs. control | **1** [160] | **1** [160] | | | | |
| **IFN-γ (16):** Blood (13), CSF (8) | Ns. Blood | Ns. CSF | ↑ Blood | ↓ Blood | ↑ CSF | ↓ CSF |
| Below detection limit | **2** [187,189] | **4** [184,187-189] | | | | |
| PD vs. control | **5** [160,166,171,190,191a] | **3** [159,160,134a] | **1** [192] | **2** [159,193] | | **1** [134e] |
| **IgG (4):** Blood (4), CSF (1) | Ns. Blood | Ns. CSF | ↑ Blood | ↓ Blood | ↑ CSF | ↓ CSF |
| PD vs. control | **4** [156,162,194,195] | **1** [194] | | | | |
| **HMGB1 (3):** Blood (3), CSF (1) | Ns. Blood | Ns. CSF | ↑ Blood | ↓ Blood | ↑ CSF | ↓ CSF |
| PD vs. control | | | **3** [178,194,196] | | **1** [194] | |
| **IL-1α (4):** Blood (3), CSF (3) | Ns. Blood | Ns. CSF | ↑ Blood | ↓ Blood | ↑ CSF | ↓ CSF |
| Below detection limit | **1** [187] | **2** [187,188] | | | | |
| PD vs. control | **1** [160] | **1** [160] | | | | |
| **IL-1β (21):** Blood (16), CSF (5) | Ns. Blood | Ns. CSF | ↑ Blood | ↓ Blood | ↑ CSF | ↓ CSF |
| Below detection limit | **2** [159,171] | **3** [159,184,188] | | | | |
| PD vs. control | **3** [160,189,197a] | **3** [160,189,198a] | **8** [168,199-205] | **1** [197e] | **2** [203, 198e] | |
| Asymptomatic mutation carriers vs. controls | | | **1** [187f] | | | |
| **IL-1RA (4):** Blood (4), CSF (2) | Ns. Blood | Ns. CSF | ↑ Blood | ↓ Blood | ↑ CSF | ↓ CSF |
| PD vs. control | | **1** [202] | | **1** [202] | | |
| **IL-2 (14):** Blood (12), CSF (5) | Ns. Blood | Ns. CSF | ↑ Blood | ↓ Blood | ↑ CSF | ↓ CSF |
| Below detection limit | **3** [159,189,206] | **3** [159,184,189] | | | | |
| PD vs. control | **4** [160,168,171,207a] | **1** [160] | **1** [169] | | | |
| **IL-4 (12):** Blood (10), CSF (5) | Ns. Blood | Ns. CSF | ↑ Blood | ↓ Blood | ↑ CSF | ↓ CSF |
| Below detection limit | **1** [159] | **2** [159,184] | | | | |
| PD vs. control | **4** [160,171,189,208,171a] | **2** [160,189] | **1** [204] | | | |
| **IL-5 (5):** Blood (4), CSF (2) | Ns. Blood | Ns. CSF | ↑ Blood | ↓ Blood | ↑ CSF | ↓ CSF |
| Below detection limit | **1** [206] | **1** [188] | | | | |
| PD vs. control | **1** [160] | **1** [160] | | | | |
| **IL-6 (30):** Blood (26), CSF (9) | Ns. Blood | Ns. CSF | ↑ Blood | ↓ Blood | ↑ CSF | ↓ CSF |
| PD vs. control | **13** [159,160,164,166,168,169, 186,189-191,209-211] | **7** [159,160,172,184,189, 191,198a] | **8** [165,170,186,193, 202,212,164b,171a] | | **1** [198e] | |

| | | | | | | |
|---|---|---|---|---|---|---|
| **IL-7 (6):** Blood (5), CSF (1) | Ns. Blood | Ns. CSF | ↑ Blood | ↓ Blood | ↑ CSF | ↓ CSF |
| PD vs. control | 1 [160] | 1 [160] | | | | |
| **IL-8 (19):** Blood (16), CSF (9) | Ns. Blood | Ns. CSF | ↑ Blood | ↓ Blood | ↑ CSF | ↓ CSF |
| PD vs. control | 7 [159,160,183,189,190,213,171a] | 5 [184,189,214,215,160g] | 2 [186,206] | 1 [159] | 1 [172] | |
| **IL-9 (3):** Blood (3) | Ns. Blood | | ↑ Blood | ↓ Blood | | |
| PD vs. control | 1 [207] | | | 1 [206] | | |
| **IL-10 (22):** Blood (19), CSF (7) | Ns. Blood | Ns. CSF | ↑ Blood | ↓ Blood | ↑ CSF | ↓ CSF |
| Below detection level | 2 [159,206] | 3 [159,184,188] | | | | |
| PD vs. control | 8 [160,168,186,189,191,202,208,171a] | 4 [160,189,202,216] | 3 [166,190,192] | 1 [212] | | |
| **IL-12 (3):** Blood (3), CSF (1) | Ns. Blood | Ns. CSF | ↑ Blood | ↓ Blood | ↑ CSF | ↓ CSF |
| Below detection limit | 1 [206] | | | | | |
| PD vs. control | 1 [160] | 1 [160] | | | | |
| **IL-12p70 (8):** Blood (7), CSF (3) | Ns. Blood | Ns. CSF | ↑ Blood | ↓ Blood | ↑ CSF | ↓ CSF |
| Below detection limit | 2 [159,171a] | 2 [159,188] | | | | |
| PD vs. control | 2 [160,190] | 1 [160] | | | | |
| **IL-13 (6):** Blood (7), CSF (2) | Ns. Blood | Ns. CSF | ↑ Blood | ↓ Blood | ↑ CSF | ↓ CSF |
| Below detection limit | 3 [159,206,171a] | 1 [159] | | | | |
| PD vs. control | 1 [160] | 1 [160] | | | | |
| **IL-15 (7):** Blood (6), CSF (2) | Ns. Blood | Ns. CSF | ↑ Blood | ↓ Blood | ↑ CSF | ↓ CSF |
| Below detection limit | 1 [206] | 1 [187] | | | | |
| PD vs. control | 3 [207,217,160h] | 1 [160h] | | | | |
| **IL-16 (3):** Blood (2), CSF (2) | Ns. Blood | Ns. CSF | ↑ Blood | ↓ Blood | ↑ CSF | ↓ CSF |
| PD vs. control | 1 [160] | 1 [160] | | | | 1 [184] |
| **IL-17A (3):** Blood (2), CSF (1) | Ns. Blood | Ns. CSF | ↑ Blood | ↓ Blood | ↑ CSF | ↓ CSF |
| PD vs. control | 2 [160,209] | 1 [160] | 1 [190] | | | |
| **IL-18 (5):** Blood (4), CSF (1) | Ns. Blood | Ns. CSF | ↑ Blood | ↓ Blood | ↑ CSF | ↓ CSF |
| PD vs. control | 1 [197a] | | 1 [218] | 1 [197e] | | |
| **IP-10 (7):** Blood (5), CSF (3) | Ns. Blood | Ns. CSF | ↑ Blood | ↓ Blood | ↑ CSF | ↓ CSF |
| PD vs. control | 2 [160,183] | 3 [160,184,215] | 1 [207] | | | |
| **MCP-1 (12):** Blood (7), CSF (6) | Ns. Blood | Ns. CSF | ↑ Blood | ↓ Blood | ↑ CSF | ↓ CSF |
| PD vs. control | 1 [160] | 5 [160,172,184,214,215] | 1 [207] | | | |
| **MDC (7):** Blood (4), CSF (4) | Ns. Blood | Ns. CSF | ↑ Blood | ↓ Blood | ↑ CSF | ↓ CSF |
| PD vs. control | 1 [160] | 4 [160,184,191,216] | 1 [219] | | | |
| **MIP-1α (8):** Blood (5), CSF (4) | Ns. Blood | Ns. CSF | ↑ Blood | ↓ Blood | ↑ CSF | ↓ CSF |
| Below detection limit | | 1 [184] | | | | |
| PD vs. control | 3 [160,183,186] | 1 [160] | | 1 [206] | 1 [191] | |
| **MIP-1β (7):** Blood (5), CSF (3) | Ns. Blood | Ns. CSF | ↑ Blood | ↓ Blood | ↑ CSF | ↓ CSF |
| PD vs. control | 2 [160,191] | 2 [160,191] | 1 [206] | | | |
| **SAA (4):** Blood (3), CSF (3) | Ns. Blood | Ns. CSF | ↑ Blood | ↓ Blood | ↑ CSF | ↓ CSF |
| PD vs. control | 3 [160,172,173] | | | | 1 [172] | |
| **TARC (3):** Blood (1), CSF (3) | Ns. Blood | Ns. CSF | ↑ Blood | ↓ Blood | ↑ CSF | ↓ CSF |
| Below detection limit | | 1 [184] | | | | |
| PD vs. control | 1 [160] | 1 [160] | | | 1 [191] | |
| **TGF-β (3):** Blood (3) | Ns. Blood | | ↑ Blood | ↓ Blood | | |
| PD vs. control | 3 [186,208,209] | | | | | |
| **TNF-α (32):** Blood (28), CSF (8) | Ns. Blood | Ns. CSF | ↑ Blood | ↓ Blood | ↑ CSF | ↓ CSF |

| | | | | | | |
|---|---|---|---|---|---|---|
| PD vs. control | **11** [160,168,169,186, 189-191,202,209,211,192i] | **5** [160,184,189,203,198a] | **10** [166,174,193,196,203, 204,207,212,192j,171a] | **2** [159,192k] | **1** [159] | **1** [198e] |
| Asymptomatic mutation carriers vs. controls | | | | | **1** [184] | |
| **<u>WBC:</u> LYM (20):** Blood (20); **NEU (15):** Blood (15); **LEUK (16):** Blood (16), CSF (1); **Mono (12):** Blood (12); **NLR (10):** Blood (10); **Baso (4):** Blood (4); **Eos (5):** Blood (4) | Ns.<br>Blood | Ns.<br>CSF | ↑<br>Blood | ↓<br>Blood | ↑<br>CSF | ↓<br>CSF |
| PD vs. control | **LYM: 10** [134,163,219-225,226f]; **NEU: 9** [175,219-223,227, 228,226f,l,m]; **Mono: 6** [134,219,222,225,227,226f,m]; **LEUK: 5** [181,219,229,226n,140e]; **NLR: 2** [181,220]; **Baso: 3** [221,222,226n]; **Eos: 2** [222,226n] | **LEUK: 1** [163] | **LYM: 3** [193,212,223]; **NEU: 5** [134,157,181,225,226n]; **Mono: 2** [181,177c]; **LEUK: 2** [157,223]; **NLR: 5** [222,223,227, 177c,226f,l,m,n] | **LYM: 10** [134,157,175, 181,222,225, 227,228, 177c,226l,m,n]; **Mono: 1** [226l,n] | | |
| **YKL-40 (5):** Blood (1), CSF (4) | Ns.<br>Blood | Ns.<br>CSF | ↑<br>Blood | ↓<br>Blood | ↑<br>CSF | ↓<br>CSF |
| PD vs. control | | **2** [5,230] | **1** [231] | | | **1** [172a] |

**Complete list biomarkers investigated in PD, ordered alphabetically.** Biomarkers and total number of studies in bold.
[a] = PD without cognitive impairment; [b] = symptomatic biallelic Parkin/PINK1 mutation carriers; [c] = PD-RBD, [d] = PD Stage 2 vs. HC; [e] = PD with cognitive impairment; [f] = LRKK mutation carriers vs HC; [g] = after correction for mulitple comparisons; h = after correction for covariates; [i] = 1-3 years PD; [j] = < 1-year PD; [k] = >3 years PD; [l] = sPD vs HC; [m] = GBA mutation carriers vs. HC; [n] = combined PD vs. HC in both cohorts.
All abbreviations appearing in this table are defined in the abbreviation list

**Supplementary table 4: Complete FTD results in different studies**

| **Biomarker** (number of studies) | **Number of studies by biofluid type** | | | | |
|---|---|---|---|---|---|
| **Eotaxin-1 (3):** Blood (1), CSF (3) | Ns. Blood | Ns. CSF | ↑ Blood | ↑ CSF | ↓ CSF |
| Below detection limit | | **1** [232] | | | |
| FTD vs. control | **1** [233a] | **2** [47,233a] | | | |
| Symptomatic vs. asymptomatic FTD | **1** [233b] | **1** [233b] | | | |
| **GM-CSF (3):** Blood (1), CSF (3) | Ns. Blood | Ns. CSF | ↑ Blood | ↑ CSF | ↓ CSF |
| Below detection limit | **1** [233a] | **2** [232,233a] | | | |
| FTD vs. control | | **1** [47] | | | |
| **IFN-γ (5):** Blood (3), CSF (3) | Ns. Blood | Ns. CSF | ↑ Blood | ↑ CSF | |
| Below detection limit | **2** [234,233a] | **2** [232,233a] | | | |
| FTD vs. control | **1** [235] | **1** [47] | | | |
| **IL-1β (5):** Blood (3), CSF (3) | Ns. Blood | Ns. CSF | ↑ Blood | ↑ CSF | ↓ CSF |
| Below detection limit | **1** [233a] | **2** [232,233a] | | | |
| FTD vs. control | **1** [235] | **1** [47] | | | |
| **IL-2 (4):** Blood (2), CSF (3) | Ns. Blood | Ns. CSF | ↑ Blood | ↑ CSF | ↓ CSF |
| Below detection limit | | **2** [232,233a] | | | |
| FTD vs. control | **1** [233a] | **1** [47] | **1** [235] | | |
| **IL-4 (4):** Blood (2), CSF (3) | Ns. Blood | Ns. CSF | ↑ Blood | ↑ CSF | ↓ CSF |
| Below detection limit | **1** [233a] | **2** [232,233a] | | | |
| FTD vs. control | **1** [235] | **1** [47] | | | |
| **IL-5 (3):** Blood (1), CSF (3) | Ns. Blood | Ns. CSF | ↑ Blood | ↑ CSF | ↓ CSF |
| Below detection limit | | **1** [232] | | | |
| FTD vs. control | **1** [233a] | **2** [47,233a] | | | |
| Symptomatic vs. asymptomatic FTD | **1** [233b] | **1** [233b] | | | |
| **IL-6 (6):** Blood (4), CSF (3) | Ns. Blood | Ns. CSF | ↑ Blood | ↑ CSF | ↓ CSF |
| FTD vs. control | **3** [235,236c,233a] | **3** [47,232,233a] | **1** [236d] | | |
| Asymptomatic FTD vs. control | **1** [237a] | **1** [236e] | | | |
| Symptomatic vs. asymptomatic FTD | **1** [233d] | **1** [233e] | **1** [237b] | | |
| Prodromal vs. asymptomatic FTD | **1** [237b] | | | | |
| **IL-8 (4):** Blood (2), CSF (3) | Ns. Blood | Ns. CSF | ↑ Blood | ↑ CSF | ↓ CSF |
| FTD vs. control | **1** [233a] | **3** [47,232,233a] | | | |
| Symptomatic vs. asymptomatic FTD | **1** [233e] | **1** [233e] | | | |
| **IL-10 (4):** Blood (2), CSF (2) | Ns. Blood | Ns. CSF | ↑ Blood | ↑ CSF | ↓ CSF |
| Below detection limit | | **1** [232] | | | |
| FTD vs. control | **1** [235] | **1** [47] | | | |
| **IL-12p70 (4):** Blood (2), CSF (3) | Ns. Blood | Ns. CSF | ↑ Blood | ↑ CSF | ↓ CSF |
| Below detection limit | | **2** [232,233a] | | | |
| FTD vs. control | **1** [233a] | **1** [47] | **1** [235] | | |
| **IL-13 (3):** Blood (1), CSF (3) | Ns. Blood | Ns. CSF | ↑ Blood | ↑ CSF | ↓ CSF |
| Below detection limit | | **2** [232,233a] | | | |
| FTD vs. control | **1** [233a] | **1** [47] | | | |
| **IL-15 (3):** Blood (1), CSF (3) | Ns. Blood | Ns. CSF | ↑ Blood | ↑ CSF | ↓ CSF |
| Below detection limit | | **1** [232] | | | |
| FTD vs. control | **1** [233a] | **2** [47,233a] | | | |
| Symptomatic vs. asymptomatic FTD | | | **1** [233b] | **1** [233b] | |
| **IL-17A (5):** Blood (3), CSF (3) | Ns. Blood | Ns. CSF | ↑ Blood | ↑ CSF | ↓ CSF |
| Below detection limit | **1** [234] | **2** [232,233a] | | | |

| | | | | | |
|---|---|---|---|---|---|
| FTD vs. control | 1 [233a] | 1 [47] | 1 [235] | | |
| **IP-10 (3):**<br>Blood (1), CSF (3) | Ns.<br>Blood | Ns.<br>CSF | ↑<br>Blood | ↑<br>CSF | ↓<br>CSF |
| Below detection limit | | | | | 1 [232] |
| FTD vs. control | 1 [233a] | 2 [47,233a] | | | |
| Symptomatic vs. asymptomatic FTD | 1 [233b] | 1 [233b] | | | |
| **MCP-1 (4):**<br>Blood (2), CSF (3) | Ns.<br>Blood | Ns.<br>CSF | ↑<br>Blood | ↑<br>CSF | ↓<br>CSF |
| FTD vs. control | 1 [233a] | 3 [47,232,233a] | | | |
| Symptomatic vs. asymptomatic FTD | 1 [233e] | 1 [233e] | | | |
| **MIP-1α (3):**<br>Blood (1), CSF (3) | Ns.<br>Blood | Ns.<br>CSF | ↑<br>Blood | ↑<br>CSF | ↓<br>CSF |
| Below detection limit | 1 [233a] | 2 [232,233a] | | | |
| FTD vs. control | | 1 [47] | | | |
| **MIP-1β (3):**<br>Blood (1), CSF (3) | Ns.<br>Blood | Ns.<br>CSF | ↑<br>Blood | ↑<br>CSF | ↓<br>CSF |
| FTD vs. control | 1 [233a] | 3 [47,232,233a] | | | |
| Symptomatic vs. asymptomatic FTD | | 1 [233b] | 1 [233b,f] | | |
| **TNF-α (6):**<br>Blood (4), CSF (3) | Ns.<br>Blood | Ns.<br>CSF | ↑<br>Blood | ↑<br>CSF | ↓<br>CSF |
| Below detection limit | | 1 [232] | | | |
| FTD vs. control | 3 [235,236g,233a] | 3 [47,236,233a] | | | |
| Asymptomatic FTD vs. control | 2 [237h,236d] | | | | |
| Symptomatic vs. asymptomatic FTD | 1 [237i] | | 1 [233b] | | |
| Prodromal vs. asymptomatic FTD | | | 1 [237i] | | |
| **YKL-40 (8):**<br>Blood (1), CSF (7) | Ns.<br>Blood | Ns.<br>CSF | ↑<br>Blood | ↑<br>CSF | ↓<br>CSF |
| FTD vs. control | | 1 [238] | | 6 [143,149,230,239-241] | |
| Asymptomatic FTD vs. control | 1 [237h] | | | | |
| Symptomatic vs. asymptomatic FTD | 1 [237i] | | | | |
| Prodromal vs. asymptomatic FTD | 1 [237i] | | | | |

**Complete list of biomarkers investigated in FTD, ordered alphabetically.** Biomarkers and total number of studies in bold.

[a] = Combined symptomatic and presymptomatic CHMP2B mutation carriers vs. non-carriers; [b] = CHMP2B mutation carriers; [c] = without GRN mutation; [d] = with GRN variant; [e] = CHMP2B mutation carriers; [f] = not corrected for multiple comparisons; [g] = with and without GRN mutation; [h] = Participants with pathogenic C9orf72, GRN, or MAPT variants, compared with non-carrier family members; [i] = Combined gene groups with pathogenic C9orf72, GRN, and MAPT variants;

All abbreviations appearing in this table are defined in the abbreviation list

**Supplementary table 5: Complete ALS results in different studies**

| **Biomarker** (number of studies) | **Number of studies by biofluid type** | | | | | |
|---|---|---|---|---|---|---|
| **CHIT1 (6):** Blood (1), CSF (5) | Ns. Blood | Ns. CSF | ↑ Blood | ↓ Blood | ↑ CSF | ↓ CSF |
| ALS vs. control | **1** [242] | **1** [243] | | | **4** [242,244-246] | |
| **Complement 3 (3):** Blood (2), CSF (1) | Ns. Blood | Ns. CSF | ↑ Blood | ↓ Blood | ↑ CSF | ↓ CSF |
| ALS vs. control | **2** [156,247] | | | | **1** [248] | |
| **CRP (7):** Blood (5), CSF (2) | Ns. Blood | Ns. CSF | ↑ Blood | ↓ Blood | ↑ CSF | ↓ CSF |
| ALS vs. control | **1** [249] | **2** [232,248] | **2** [250,251] | | | |
| **G-CSF (3):** Blood (1), CSF (2) | Ns. Blood | Ns. CSF | ↑ Blood | ↓ Blood | ↑ CSF | ↓ CSF |
| ALS vs. control | | | | **1** [252] | **2** [253,254] | |
| **IFN-y (10):** Blood (8), CSF (2) | Ns. Blood | Ns. CSF | ↑ Blood | ↓ Blood | ↑ CSF | ↓ CSF |
| Below detection limit | | **1** [232] | | | | |
| ALS vs. control | **4** [249,252,255,256] | | **3** [257-259] | **1** [260] | | **1** [253] |
| **IL-1β (6):** Blood (4), CSF (2) | Ns. Blood | Ns. CSF | ↑ Blood | ↓ Blood | ↑ CSF | ↓ CSF |
| Below detection limit | | **1** [232] | | | | |
| ALS vs. control | **1** [249] | **1** [254] | **2** [258,260] | **1** [252] | | |
| **IL-2 (7):** Blood (4), CSF (3) | Ns. Blood | Ns. CSF | ↑ Blood | ↓ Blood | ↑ CSF | ↓ CSF |
| Below detection limit | | **1** [232] | | | | |
| ALS vs. control | **2** [256,260] | | **1** [259] | **1** [252] | **2** [253,254] | |
| **IL-4 (5):** Blood (3), CSF (2) | Ns. Blood | Ns. CSF | ↑ Blood | ↓ Blood | ↑ CSF | ↓ CSF |
| Below detection limit | | **1** [232] | | | | |
| ALS vs. control | **2** [252,256] | **1** [254] | **1** [260] | | | |
| **IL-6 (13):** Blood (9), CSF (3) | Ns. Blood | Ns. CSF | ↑ Blood | ↓ Blood | ↑ CSF | ↓ CSF |
| ALS vs. control | **3** [249,261,262] | | **5** [256,258,259, 263,264] | **1** [252] | **3** [232,253,254] | |
| **IL-8 (8):** Blood (5), CSF, (8) | Ns. Blood | Ns. CSF | ↑ Blood | ↓ Blood | ↑ CSF | ↓ CSF |
| ALS vs. control | **2** [261,264] | **3** [232,253,254] | **3** [252,256,263] | | | |
| **IL-10 (9):** Blood (6), CSF (3) | Ns. Blood | Ns. CSF | ↑ Blood | ↓ Blood | ↑ CSF | ↓ CSF |
| Below detection limit | | **1** [232] | | | | |
| ALS vs. control | **3** [249,256,263] | **1** [254] | **1** [259] | **2** [252,258] | | **1** [253] |
| **IL-15 (3):** Blood (1), CSF (2) | Ns. Blood | Ns. CSF | ↑ Blood | ↓ Blood | ↑ CSF | ↓ CSF |
| Below detection limit | | **1** [232] | | | | |
| ALS vs. control | | | **1** [252] | | **1** [253] | |
| **IL-17 (3):** Blood (1), CSF (2) | Ns. Blood | Ns. CSF | ↑ Blood | ↓ Blood | ↑ CSF | ↓ CSF |
| ALS vs. control | **1** [256] | | | | **2** [253,254] | |
| **IL-17A (4):** Blood (3), CSF (1) | Ns. Blood | Ns. CSF | ↑ Blood | ↓ Blood | ↑ CSF | ↓ CSF |
| Below detection limit | | **1** [232] | | | | |
| ALS vs. control | **3** [249,252,258] | | | | | |
| **MCP-1 (9):** Blood (5), CSF (5) | Ns. Blood | Ns. CSF | ↑ Blood | ↓ Blood | ↑ CSF | ↓ CSF |
| ALS vs. control | **3** [256,261,265] | **1** [232] | **1** [266] | **1** [252] | **4** [244,245,253,266] | |
| **MIP-1α (5):** Blood (2), CSF (4) | Ns. Blood | Ns. CSF | ↑ Blood | ↓ Blood | ↑ CSF | ↓ CSF |
| Below detection limit | | **1** [232] | | | | |
| ALS vs. control | | **1** [254] | **2** [253,267] | **1** [252] | **1** [267] | |
| **TNF-α (11):** Blood (9), CSF (2) | Ns. Blood | Ns. CSF | ↑ Blood | ↓ Blood | ↑ CSF | ↓ CSF |
| Below detection limit | | **1** [232] | | | | |
| ALS vs. control | **5** [249,256,258,260,263] | **1** [254] | **3** [257,259,264] | **1** [252] | | |
| **WBC:** **NLR (8):** Blood (8); | Ns. Blood | Ns. CSF | ↑ Blood | ↓ Blood | ↑ CSF | ↓ CSF |

| | | | | | | |
|---|---|---|---|---|---|---|
| **LEUK (6):** Blood (5), CSF (1); **Mono (6):** Blood (6); **LYM (5):** Blood (4), CSF (1); **NEU (4):** Blood (4); **NK cell (3):** Blood (3), CSF (1) | | | | | | |
| ALS vs. control | **NLR: 2** [249,254]; **LEUK: 2** [195,268]; **Mono: 3** [195,251,269]; **LYM: 3** [195,251,258]; **NEU: 1** [195]; **NK-cell: 1** [270] | **LEUK: 1** [269]; **NK cell: 1** [258] | **NLR: 2** [251,268] **LEUK: 1** [249]; **Mono: 1** [271a]; **NEU: 1** [251]; **NK cell: 1** [258] | | **LYM: 1** [258] | |
| **YKL-40 (6):** Blood (2), CSF (6) | Ns. Blood | Ns. CSF | ↑ Blood | ↓ Blood | ↑ CSF | ↓ CSF |
| ALS vs. control | **2** [242,272] | **1** [272] | | | **5** [242,243,245,246,273] | |

**Complete list of biomarkers investigated in ALS, ordered alphabetically.** Biomarkers and total number of studies in bold.

[a] = compared to normal range.

All abbreviations appearing in this table are defined in the abbreviation list

**Supplementary table 6: Complete DLB results in different studies**

| **Biomarker** (number of studies) | **Number of studies by biofluid type** | | | |
|---|---|---|---|---|
| **CRP (3):** Blood (2), CSF (1) | Ns. Blood | Ns. CSF | ↑ Blood | ↓ Blood |
| DLB vs. control | **2** [23,95] | **1** [232] | | |
| **Eotaxin-1 (3):** Blood (1), CSF (2) | Ns. Blood | Ns. CSF | ↑ Blood | ↓ Blood |
| Below detection limit | | **2** [215,232] | | |
| DLB vs. control | **1** [274] | | | |
| **Eotaxin-3 (3):** Blood (1), CSF (2) | Ns. Blood | Ns. CSF | ↑ Blood | ↓ Blood |
| Below detection limit | | **2** [215,232] | | |
| DLB vs. control | **1** [274] | | | |
| **IFN-γ (4):** Blood (3), CSF (1) | Ns. Blood | Ns. CSF | ↑ Blood | ↓ Blood |
| Below detection limit | | **1** [232] | | |
| DLB vs. control | **3** [23,95,275] | | | |
| **IL-1β (5):** Blood (4), CSF (1) | Ns. Blood | Ns. CSF | ↑ Blood | ↓ Blood |
| Below detection limit | **1** [274] | **1** [232] | | |
| DLB vs. control | **1** [23] | | **2** [95,275] | |
| **IL-2 (5):** Blood (4), CSF (1) | Ns. Blood | Ns. CSF | ↑ Blood | ↓ Blood |
| Below detection limit | | **1** [232] | | |
| DLB vs. control | **2** [23,275] | | **2** [95,274] | |
| **IL-4 (5):** Blood (4), CSF (1) | Ns. Blood | Ns. CSF | ↑ Blood | ↓ Blood |
| Below detection limit | **1** [274] | **1** [232] | | |
| DLB vs. control | **2** [23,275] | | **1** [95] | |
| **IL-6 (6):** Blood (5), CSF (1) | Ns. Blood | Ns. CSF | ↑ Blood | ↓ Blood |
| DLB vs. control | **3** [23,95,274] | **1** [232] | **2** [103,275] | |
| **IL-8 (6):** Blood (4), CSF (2) | Ns. Blood | Ns. CSF | ↑ Blood | ↓ Blood |
| DLB vs. control | **3** [23,95,275] | **2** [215,232] | | **1** [274] |
| **IL-10 (5):** Blood (4), CSF (1) | Ns. Blood | Ns. CSF | ↑ Blood | ↓ Blood |
| Below detection limit | | **1** [232] | | |
| DLB vs. control | **3** [23,274,275] | | **1** [95] | |
| **IL-13 (5):** Blood (4), CSF (1) | Ns. Blood | Ns. CSF | ↑ Blood | ↓ Blood |
| Below detection limit | **2** [23,95] | **1** [232] | | |
| DLB vs. control | **2** [274,275] | | | |
| **IL-12p70 (4):** Blood (3), CSF (1) | Ns. Blood | Ns. CSF | ↑ Blood | ↓ Blood |
| Below detection limit | **2** [23,95] | **1** [232] | | |
| DLB vs. control | **1** [274] | | | |
| **IP-10 (3):** Blood (1), CSF (2) | Ns. Blood | Ns. CSF | ↑ Blood | ↓ Blood |
| DLB vs. control | **1** [274] | **2** [215,232] | | |
| **MCP-1 (4)** Blood (1), CSF (3) | Ns. Blood | Ns. CSF | ↑ Blood | ↓ Blood |
| DLB vs. control | **1** [274] | **3** [215,232,276] | | |
| **MCP-4 (3):** Blood (1), CSF (2) | Ns. Blood | Ns. CSF | ↑ Blood | ↓ Blood |
| Below detection limit | | **2** [215,232] | | |
| DLB vs. control | **1** [274] | | | |
| **MDC (3):** Blood (1), CSF (2) | Ns. Blood | Ns. CSF | ↑ Blood | ↓ Blood |
| Below detection limit | | **2** [215,232] | | |
| DLB vs. control | **1** [274] | | | |
| **MIP-1β (3):** Blood (1), CSF (2) | Ns. Blood | Ns. CSF | ↑ Blood | ↓ Blood |
| Below detection limit | | **1** [215] | | |

| | | | | |
|---|---|---|---|---|
| DLB vs. control | **1** [274] | **1** [232] | | |
| **TARC (3):**<br>Blood (1), CSF (2) | Ns.<br>Blood | Ns.<br>CSF | ↑<br>Blood | ↓<br>Blood |
| Below detection limit | | **2** [215,232] | | |
| DLB vs. control | **1** [274] | | | |
| **TNF-α (5):**<br>Blood (4), CSF (1) | Ns.<br>Blood | Ns.<br>CSF | ↑<br>Blood | ↓<br>Blood |
| Below detection limit | | **1** [232] | | |
| DLB vs. control | **3** [23,274,275] | | | **1** [95] |
| **YKL-40 (3):**<br>Blood (2), CSF (1) | Ns.<br>Blood | Ns.<br>CSF | ↑<br>Blood | ↓<br>Blood |
| DLB vs. control | **2** [274,277] | **1** [215] | | |

**Complete list of biomarkers investigated in DLB, ordered alphabetically.** Biomarkers and total number of studies in bold.

All abbreviations appearing in this table are defined in the abbreviation list

## References


1. Faura J, Bustamante A, Penalba A, et al. CCL23: A Chemokine Associated with Progression from Mild Cognitive Impairment to Alzheimer's Disease. Article. *Journal of Alzheimers Disease*. 2020;73(4):1585–1595. doi:10.3233/jad-190753
2. Morgan AR, Touchard S, Leckey C, et al. Inflammatory biomarkers in Alzheimer's disease plasma. *Alzheimers Dement*. 2019;15(6):776–787. doi:10.1016/j.jalz.2019.03.007
3. Brosseron F, Maass A, Kleineidam L, et al. Serum IL-6, sAXL, and YKL-40 as systemic correlates of reduced brain structure and function in Alzheimer's disease: results from the DELCODE study. *Alzheimers Res Ther*. 2023;15(1):13. doi:10.1186/s13195-022-01118-0
4. Brosseron F, Traschutz A, Widmann CN, et al. Characterization and clinical use of inflammatory cerebrospinal fluid protein markers in Alzheimer's disease. Article. *Alzheimers Research & Therapy*. Feb 2018;10:14. 25. doi:10.1186/s13195-018-0353-3
5. Brosseron F, Kolbe CC, Santarelli F, et al. Multicenter Alzheimer's and Parkinson's disease immune biomarker verification study. *Alzheimers Dement*. 2020;16(2):292–304. doi:10.1016/j.jalz.2019.07.018
6. Brosseron F, Maass A, Kleineidam L, et al. Soluble TAM receptors sAXL and sTyro3 predict structural and functional protection in Alzheimer's disease. Article. *Neuron*. Mar 2022;110(6):1009–+. doi:10.1016/j.neuron.2021.12.016
7. Wang T, Wang X, Yao Y, et al. Association of plasma apolipoproteins and levels of inflammation-related factors with different stages of Alzheimer's disease: a cross-sectional study. *BMJ Open*. 2022;12(4):e054347. doi:10.1136/bmjopen-2021-054347
8. Toledo JB, Korff A, Shaw LM, Trojanowski JQ, Zhang J, Alzheimer's Dis N. Low levels of cerebrospinal fluid complement 3 and factor H predict faster cognitive decline in mild cognitive impairment. Article. *Alzheimers Research & Therapy*. 2014;6(3):10. 36. doi:10.1186/alzrt266
9. Bonham LW, Desikan RS, Yokoyama JS, Alzheimer's Dis Neuroimaging I. The relationship between complement factor C3, APOE epsilon 4, amyloid and tau in Alzheimer's disease. Article. *Acta Neuropathologica Communications*. Jun 2016;4:7. 65. doi:10.1186/s40478-016-0339-y
10. Muenchhoff J, Poljak A, Thalamuthu A, et al. Changes in the plasma proteome at asymptomatic and symptomatic stages of autosomal dominant Alzheimer's disease. Article. *Scientific Reports*. Jul 2016;6:11. 29078. doi:10.1038/srep29078
11. Shim KH, Kim D, Kang MJ, et al. Subsequent correlated changes in complement component 3 and amyloid beta oligomers in the blood of patients with Alzheimer's disease. *Alzheimers Dement*. Apr 2024;20(4):2731–2741. doi:10.1002/alz.13734
12. Hao J, Qiao Y, Li T, et al. Investigating Changes in the Serum Inflammatory Factors in Alzheimer's Disease and Their Correlation with Cognitive Function. *J Alzheimers Dis*. 2021;84(2):835–842. doi:10.3233/JAD-210552
13. Ayton S, Janelidze S, Roberts B, et al. Acute phase markers in CSF reveal inflammatory changes in Alzheimer's disease that intersect with pathology, APOE ε 4, sex and age. *Prog Neurobiol*. 2021;198:101904. doi:10.1016/j.pneurobio.2020.101904
14. Heslegrave A, Heywood W, Paterson R, et al. Increased cerebrospinal fluid soluble TREM2 concentration in Alzheimer's disease. Article. *Molecular Neurodegeneration*. Jan 2016;11:7. 3. doi:10.1186/s13024-016-0071-x
15. Vacinova G, Vejražkova D, Rusina R, et al. Regulated upon activation, normal T cell expressed and secreted (RANTES) levels in the peripheral blood of patients with Alzheimer's disease. *Neural Regen Res*. 2021;16(4):796–800. doi:10.4103/1673-5374.295340
16. Kuyumcu ME, Yesil Y, Ozturk ZA, et al. The Evaluation of Neutrophil-Lymphocyte Ratio in Alzheimer's Disease. Article. *Dementia and Geriatric Cognitive Disorders*. 2012;34(2):69–74. doi:10.1159/000341583
17. Öztürk ZA, Ünal A, Yiğiter R, et al. Is increased red cell distribution width (RDW) indicating the inflammation in Alzheimer's disease (AD)? *Arch Gerontol Geriatr*. 2013;56(1):50–4. doi:10.1016/j.archger.2012.10.002

18. Davis G, Baboolal N, Nayak S, McRae A. Sialic acid, homocysteine and CRP: Potential markers for dementia. Article. *Neuroscience Letters*. Nov 2009;465(3):282–284. doi:10.1016/j.neulet.2009.09.035
19. Blanco-Palmero VA, Rubio-Fernandez M, Antequera D, et al. Increased YKL-40 but Not C-Reactive Protein Levels in Patients with Alzheimer's Disease. Article. *Biomedicines*. Sep 2021;9(9):14. 1094. doi:10.3390/biomedicines9091094
20. Bekris LM, Khrestian M, Dyne E, et al. Soluble TREM2 and biomarkers of central and peripheral inflammation in neurodegenerative disease. Article. *Journal of Neuroimmunology*. Jun 2018;319:19–27. doi:10.1016/j.jneuroim.2018.03.003
21. Thaker AA, McConnell BV, Rogers DM, et al. Astrogliosis, neuritic microstructure, and sex effects: GFAP is an indicator of neuritic orientation in women. *Brain Behav Immun*. 2023;113:124–135. doi:10.1016/j.bbi.2023.06.026
22. Kizilarslanoğlu MC, Kara Ö, Yeşil Y, et al. Alzheimer disease, inflammation, and novel inflammatory marker: resistin. *Turk J Med Sci*. 2015;45(5):1040–6.
23. King E, O'Brien JT, Donaghy P, et al. Peripheral inflammation in prodromal Alzheimer's and Lewy body dementias. *J Neurol Neurosurg Psychiatry*. Apr 2018;89(4):339–345. doi:10.1136/jnnp-2017-317134
24. Magalhães CA, Ferreira CN, Loures CMG, et al. Leptin, hsCRP, TNF-α and IL-6 levels from normal aging to dementia: Relationship with cognitive and functional status. *J Clin Neurosci*. 2018;56:150–155. doi:10.1016/j.jocn.2018.08.027
25. Mulder SD, Hack CE, van der Flier WM, Scheltens P, Blankenstein MA, Veerhuis R. Evaluation of Intrathecal Serum Amyloid P (SAP) and C-Reactive Protein (CRP) Synthesis in Alzheimer's Disease with the Use of Index Values. Article. *Journal of Alzheimers Disease*. 2010;22(4):1073–1079. doi:10.3233/jad-2010-100888
26. Alexandrov PN, Kruck TP, Lukiw WJ. Nanomolar aluminum induces expression of the inflammatory systemic biomarker C-reactive protein (CRP) in human brain microvessel endothelial cells (hBMECs). *J Inorg Biochem*. 2015;152:210–3. doi:10.1016/j.jinorgbio.2015.07.013
27. Mancinella A, Mancinella M, Carpinteri G, et al. Is there a relationship between high C-reactive protein (CRP) levels and dementia? *Arch Gerontol Geriatr*. 2009;49 Suppl 1:185–94. doi:10.1016/j.archger.2009.09.028
28. Helmy AA, Naseer MM, Shafie SE, Nada MA. Role of interleukin 6 and alpha-globulins in differentiating Alzheimer and vascular dementias. *Neurodegener Dis*. 2012;9(2):81–6. doi:10.1159/000329568
29. Gupta A, Watkins A, Thomas P, et al. Coagulation and inflammatory markers in Alzheimer's and vascular dementia. *Int J Clin Pract*. 2005;59(1):52–7. doi:10.1111/j.1742-1241.2004.00143.x
30. Pocevičiūtė D, Nuñez-Diaz C, Roth B, et al. Increased plasma and brain immunoglobulin A in Alzheimer's disease is lost in apolipoprotein E ε4 carriers. *Alzheimers Res Ther*. 2022;14(1):117. doi:10.1186/s13195-022-01062-z
31. Devčić S, Glamuzina L, Ruljancic N, Mihanovic M. There are no differences in IL-6, CRP and homocystein concentrations between women whose mothers had AD and women whose mothers did not have AD. *Psychiatry Res*. 2014;220(3):970–4. doi:10.1016/j.psychres.2014.08.059
32. Song IU, Chung SW, Kim YD, Maeng LS. Relationship between the hs-CRP as non-specific biomarker and Alzheimer's disease according to aging process. *Int J Med Sci*. 2015;12(8):613–7. doi:10.7150/ijms.12742
33. Cervellati C, Trentini A, Bosi C, et al. Low-grade systemic inflammation is associated with functional disability in elderly people affected by dementia. *Geroscience*. 2018;40(1):61–69. doi:10.1007/s11357-018-0010-6
34. Yarchoan M, Louneva N, Xie SX, et al. Association of plasma C-reactive protein levels with the diagnosis of Alzheimer's disease. *J Neurol Sci*. Oct 15 2013;333(1-2):9–12. doi:10.1016/j.jns.2013.05.028

35. Hazen J, Vistnes M, Barca ML, et al. The Association Between Circulating Inflammatory Markers and the Progression of Alzheimer Disease in Norwegian Memory Clinic Patients With Mild Cognitive Impairment or Dementia. *Alzheimer Dis Assoc Disord*. 2020;34(1):47–53. doi:10.1097/WAD.0000000000000342
36. Jin J, Lu L, Hua K, Fang L, Li X, Li W. Correlations between SHBG, Sex Hormones, Inflammation, and Neurocognitive Decline in Alzheimer's Disease: A Retrospective Study. *Curr Alzheimer Res*. 2024;21(7):491–502. doi:10.2174/0115672050341904241111082935
37. Daniilidou M, Holleman J, Hagman G, et al. Neuroinflammation, cerebrovascular dysfunction and diurnal cortisol biomarkers in a memory clinic cohort: Findings from the Co-STAR study. *Transl Psychiatry*. Sep 9 2024;14(1):364. doi:10.1038/s41398-024-03072-x
38. Schmidt-Morgenroth I, Michaud P, Gasparini F, Avrameas A. Central and Peripheral Inflammation in Mild Cognitive Impairment in the Context of Alzheimer's Disease. *Int J Mol Sci*. 2023;24(13)doi:10.3390/ijms241310523
39. Guduguntla BA, Vasbinder A, Anderson E, et al. Biomarkers of chronic inflammation and cognitive decline: A prospective observational study. *Alzheimers Dement (Amst)*. Jan–Mar 2024;16(1):e12568. doi:10.1002/dad2.12568
40. Grewal R, Haghighi M, Huang S, et al. Identifying biomarkers of dementia prevalent among amnestic mild cognitively impaired ethnic female patients. Article. *Alzheimers Research & Therapy*. Oct 2016;8:11. 43. doi:10.1186/s13195-016-0211-0
41. Wennstrom M, Surova Y, Hall S, et al. The Inflammatory Marker YKL-40 Is Elevated in Cerebrospinal Fluid from Patients with Alzheimer's but Not Parkinson's Disease or Dementia with Lewy Bodies. Article. *Plos One*. Aug 2015;10(8):13. e0135458. doi:10.1371/journal.pone.0135458
42. Wang Y, Emre C, Gyllenhammar-Schill H, et al. Cerebrospinal Fluid Inflammatory Markers in Alzheimer's Disease: Influence of Comorbidities. Article. *Current Alzheimer Research*. 2021;18(2):157–170. doi:10.2174/1567205018666210330162207
43. Leung R, Proitsi P, Simmons A, et al. Inflammatory proteins in plasma are associated with severity of Alzheimer's disease. *PLoS One*. 2013;8(6):e64971. doi:10.1371/journal.pone.0064971
44. Boccardi V, Paolacci L, Remondini D, et al. Cognitive Decline and Alzheimer's Disease in Old Age: A Sex-Specific Cytokinome Signature. *J Alzheimers Dis*. 2019;72(3):911–918. doi:10.3233/JAD-190480
45. Yu S, Liu YP, Liu HL, et al. Serum Protein-Based Profiles as Novel Biomarkers for the Diagnosis of Alzheimer's Disease. Article. *Molecular Neurobiology*. May 2018;55(5):3999–4008. doi:10.1007/s12035-017-0609-0
46. de la Monte SM, Tong M, Hapel AJ. Concordant and Discordant Cerebrospinal Fluid and Plasma Cytokine and Chemokine Responses in Mild Cognitive Impairment and Early-Stage Alzheimer's Disease. *BIOMEDICINES*. 2023;11(9)doi:10.3390/biomedicines11092394
47. Taipa R, das Neves SP, Sousa AL, et al. Proinflammatory and anti-inflammatory cytokines in the CSF of patients with Alzheimer's disease and their correlation with cognitive decline. *Neurobiol Aging*. 2019;76:125–132. doi:10.1016/j.neurobiolaging.2018.12.019
48. Furney SJ, Kronenberg D, Simmons A, et al. Combinatorial Markers of Mild Cognitive Impairment Conversion to Alzheimer's Disease - Cytokines and MRI Measures Together Predict Disease Progression. Article. *Journal of Alzheimers Disease*. 2011;26:395–405. doi:10.3233/jad-2011-0044
49. Pedrini S, Gupta VB, Hone E, et al. A blood-based biomarker panel indicates IL-10 and IL-12/23p40 are jointly associated as predictors of beta-amyloid load in an AD cohort. *Scientific Reports*. 2017;7:12. doi:10.1038/s41598-017-14020-9
50. Hakobyan S, Harding K, Aiyaz M, et al. Complement Biomarkers as Predictors of Disease Progression in Alzheimer's Disease. Article. *Journal of Alzheimers Disease*. 2016;54(2):707–716. doi:10.3233/jad-160420
51. Wu YY, Hsu JL, Wang HC, Wu SJ, Hong CJ, Cheng IHJ. Alterations of the Neuroinflammatory Markers IL-6 and TRAIL in Alzheimer's Disease. Article. *Dementia and Geriatric Cognitive Disorders Extra*. 2015;5(3):424–434. doi:10.1159/000439214

52. Nordengen K, Kirsebom BE, Henjum K, et al. Glial activation and inflammation along the Alzheimer's disease continuum. Article. *Journal of Neuroinflammation*. Feb 2019;16:13. 46. doi:10.1186/s12974-019-1399-2
53. Kulczynska-Przybik A, Slowik A, Mroczko P, et al. Cerebrospinal Fluid and Blood CX3CL1 as a Potential Biomarker in Early Diagnosis and Prognosis of Dementia. Article. *Current Alzheimer Research*. 2020;17(8):709–721. doi:10.2174/1567205017666201109095657
54. Dong Y, Lagarde J, Xicota L, et al. Neutrophil hyperactivation correlates with Alzheimer's disease progression. *Ann Neurol*. 2018;83(2):387–405. doi:10.1002/ana.25159
55. Ferrari-Souza JP, Ferreira PCL, Bellaver B, et al. Astrocyte biomarker signatures of amyloid-beta and tau pathologies in Alzheimer's disease. Article; Early Access. *Molecular Psychiatry*.9. doi:10.1038/s41380-022-01716-2
56. Chiotis K, Johansson C, Rodriguez-Vieitez E, et al. Tracking reactive astrogliosis in autosomal dominant and sporadic Alzheimer's disease with multi-modal PET and plasma GFAP. *Mol Neurodegener*. 2023;18(1):60. doi:10.1186/s13024-023-00647-y
57. Elahi FM, Casaletto KB, La Joiel R, et al. Plasma biomarkers of astrocytic and neuronal dysfunction in early- and late-onset Alzheimer's. Article. *Alzheimers & Dementia*. Apr 2020;16(4):681–695. doi:10.1016/j.jalz.2019.09.004
58. Foley KE, Winder Z, Sudduth TL, et al. Alzheimer's disease and inflammatory biomarkers positively correlate in plasma in the UK-ADRC cohort. *Alzheimers Dement*. Feb 2024;20(2):1374–1386. doi:10.1002/alz.13485
59. Vrillon A, Bousiges O, Gotze K, et al. Plasma biomarkers of amyloid, tau, axonal, and neuroinflammation pathologies in dementia with Lewy bodies. *Alzheimers Res Ther*. Jul 3 2024;16(1):146. doi:10.1186/s13195-024-01502-y
60. Johnson SC, Suárez-Calvet M, Suridjan I, et al. Identifying clinically useful biomarkers in neurodegenerative disease through a collaborative approach: the NeuroToolKit. *Alzheimers Res Ther*. 2023;15(1):25. doi:10.1186/s13195-023-01168-y
61. Van Hulle C, Jonaitis EM, Betthauser TJ, et al. An examination of a novel multipanel of CSF biomarkers in the Alzheimer's disease clinical and pathological continuum. Article. *Alzheimers & Dementia*. Mar 2021;17(3):431–445. doi:10.1002/alz.12204
62. Koca S, Kiris I, Sahin S, et al. Decreased levels of cytokines implicate altered immune response in plasma of moderate-stage Alzheimer?s disease patients. Article. *Neuroscience Letters*. Aug 2022;786:8. 136799. doi:10.1016/j.neulet.2022.136799
63. Tran-Chi VL, Maes M, Nantachai G, et al. Cytokine dysregulation in amnestic mild cognitive impairment. *Sci Rep*. Sep 28 2024;14(1):22486. doi:10.1038/s41598-024-73099-z
64. Trombetta BA, Wu CY, Kuo E, et al. Cerebrospinal fluid biomarker profiling of diverse pathophysiological domains in Alzheimer's disease. *Alzheimers Dement (N Y)*. Jan–Mar 2024;10(1):e12440. doi:10.1002/trc2.12440
65. Wennstrom M, Nielsen HM, Orhan F, Londos E, Minthon L, Erhardt S. Kynurenic Acid Levels in Cerebrospinal Fluid from Patients with Alzheimer's Disease or Dementia with Lewy Bodies. Article. *International Journal of Tryptophan Research*. 2014;7:1–7. doi:10.4137/ijtrtr.S13958
66. Hu WT, Ozturk T, Kollhoff A, Wharton W, Howell JC, Alzheimer's Dis Neuroimaging I. Higher CSF sTNFR1-related proteins associate with better prognosis in very early Alzheimer's disease. Article. *Nature Communications*. Jun 2021;12(1):12. 4001. doi:10.1038/s41467-021-24220-7
67. Reale M, Kamal MA, Velluto L, Gambi D, Di Nicola M, Greig NH. Relationship between Inflammatory Mediators, A beta Levels and ApoE Genotype in Alzheimer Disease. Article. *Current Alzheimer Research*. May 2012;9(4):447–457. doi:10.2174/156720512800492549
68. Faria MC, Gonçalves GS, Rocha NP, et al. Increased plasma levels of BDNF and inflammatory markers in Alzheimer's disease. *J Psychiatr Res*. 2014;53:166–72. doi:10.1016/j.jpsychires.2014.01.019
69. D'Anna L, Abu-Rumeileh S, Fabris M, et al. Serum Interleukin-10 Levels Correlate with Cerebrospinal Fluid Amyloid Beta Deposition in Alzheimer Disease Patients. Article. *Neurodegenerative Diseases*. 2017;17(4-5):227–234. doi:10.1159/000474940

70. Mattsson N, Tabatabaei S, Johansson P, et al. Cerebrospinal Fluid Microglial Markers in Alzheimer's Disease: Elevated Chitotriosidase Activity but Lack of Diagnostic Utility. Article. *Neuromolecular Medicine*. Jun 2011;13(2):151–159. doi:10.1007/s12017-011-8147-9
71. Shateri S, Khatami SH, Haghbin Toutounchi A, et al. Plasma cytokines profile in patients with Alzheimer's and Parkinson's Disease: a comparative study in terms of inflammation. *Int J Neurosci*. 2023:1–10. doi:10.1080/00207454.2023.2292951
72. Erhardt EB, Adair JC, Knoefel JE, et al. Inflammatory Biomarkers Aid in Diagnosis of Dementia. Article. *Frontiers in Aging Neuroscience*. Aug 2021;13:11. 717344. doi:10.3389/fnagi.2021.717344
73. Iulita MF, Ganesh A, Pentz R, et al. Identification and Preliminary Validation of a Plasma Profile Associated with Cognitive Decline in Dementia and At-Risk Individuals: A Retrospective Cohort Analysis. Article. *Journal of Alzheimers Disease*. 2019;67(1):327–341. doi:10.3233/jad-180970
74. Cisbani G, Koppel A, Knezevic D, Suridjan I, Mizrahi R, Bazinet RP. Peripheral cytokine and fatty acid associations with neuroinflammation in AD and aMCI patients: An exploratory study. Article. *Brain Behavior and Immunity*. Jul 2020;87:679–688. doi:10.1016/j.bbi.2020.02.014
75. Corsi MM, Licastro F, Porcellini E, et al. Reduced plasma levels of P-selectin and L-selectin in a pilot study from Alzheimer disease: relationship with neuro-degeneration. Article. *Biogerontology*. Oct 2011;12(5):451–454. doi:10.1007/s10522-011-9335-6
76. Hasni DSM, Lim SM, Chin AV, et al. Peripheral cytokines, C-X-C motif ligand10 and interleukin-13, are associated with Malaysian Alzheimer's disease. Article. *Geriatrics & Gerontology International*. May 2017;17(5):839–846. doi:10.1111/ggi.12783
77. Belkhelfa M, Rafa H, Medjeber O, et al. IFN-gamma and TNF-alpha Are Involved During Alzheimer Disease Progression and Correlate with Nitric Oxide Production: A Study in Algerian Patients. Article. *Journal of Interferon and Cytokine Research*. Nov 2014;34(11):839–847. doi:10.1089/jir.2013.0085
78. Zhao SJ, Guo CN, Wang MQ, Chen WJ, Zhao YB. Serum levels of inflammation factors and cognitive performance in amnestic mild cognitive impairment: A Chinese clinical study. Article. *Cytokine*. Feb 2012;57(2):221–225. doi:10.1016/j.cyto.2011.11.006
79. Gongora-Rivera F, Gonzalez-Aquines A, Ortiz-Jimenez X, de la Garza CM, Salinas-Carmona M. Chemokine profile in Alzheimer's disease: Results from a Mexican population. Article. *Journal of Clinical Neuroscience*. Mar 2020;73:159–161. doi:10.1016/j.jocn.2019.12.051
80. Italiani P, Puxeddu I, Napoletano S, et al. Circulating levels of IL-1 family cytokines and receptors in Alzheimer's disease: new markers of disease progression? *J Neuroinflammation*. 2018;15(1):342. doi:10.1186/s12974-018-1376-1
81. Dursun E, Gezen-Ak D, Hanağası H, et al. The interleukin 1 alpha, interleukin 1 beta, interleukin 6 and alpha-2-macroglobulin serum levels in patients with early or late onset Alzheimer's disease, mild cognitive impairment or Parkinson's disease. *J Neuroimmunol*. 2015;283:50–7. doi:10.1016/j.jneuroim.2015.04.014
82. Julian A, Dugast E, Ragot S, et al. There is no correlation between peripheral inflammation and cognitive status at diagnosis in Alzheimer's disease. Article. *Aging Clinical and Experimental Research*. Oct 2015;27(5):589–594. doi:10.1007/s40520-015-0332-5
83. Sala G, Galimberti G, Canevari C, et al. Peripheral cytokine release in Alzheimer patients: correlation with disease severity. Article. *Neurobiology of Aging*. Nov 2003;24(7):909–914. doi:10.1016/s0197-4580(03)00010-1
84. Le Page A, Garneau H, Dupuis G, et al. Differential Phenotypes of Myeloid-Derived suppressor and T regulatory cells and cytokine levels in amnestic Mild cognitive impairment subjects compared to Mild alzheimer Diseased Patients. Article. *Frontiers in Immunology*. Jul 2017;8:10. 783. doi:10.3389/fimmu.2017.00783
85. Spitzer P, Weinbeer J, Herrmann M, et al. Analysis of Surface Levels of IL-1 Receptors and Macrophage Scavenger Receptor I in Peripheral Immune Cells of Patients With Alzheimer Disease. Article. *Journal of Geriatric Psychiatry and Neurology*. Jul 2019;32(4):211–220. doi:10.1177/0891988719841728

86. Jing X, Wang L, Song M, et al. Serum neurofilament light chain and inflammatory cytokines as biomarkers for early detection of mild cognitive impairment. *Sci Rep*. Apr 20 2024;14(1):9072. doi:10.1038/s41598-024-59530-5
87. Yang C, Wu X, Feng J, et al. Clinical values of serum C5a in Alzheimer's disease patients with different dementia stages. *Neurosci Lett*. Jul 27 2024;836:137833. doi:10.1016/j.neulet.2024.137833
88. Rui W, Xiao H, Fan Y, et al. Systemic inflammasome activation and pyroptosis associate with the progression of amnestic mild cognitive impairment and Alzheimer's disease. *J Neuroinflammation*. 2021;18(1):280. doi:10.1186/s12974-021-02329-2
89. Scarabino D, Peconi M, Broggio E, et al. Relationship between proinflammatory cytokines (Il-1beta, Il-18) and leukocyte telomere length in mild cognitive impairment and Alzheimer's disease. Article. *Experimental Gerontology*. Jul 2020;136:7. 110945. doi:10.1016/j.exger.2020.110945
90. Saresella M, Marventano I, Piancone F, et al. IL-33 and its decoy sST2 in patients with Alzheimer's disease and mild cognitive impairment. *J Neuroinflammation*. 2020;17(1):174. doi:10.1186/s12974-020-01806-4
91. Yu Y, Lv J, Ma D, et al. Microglial ApoD-induced NLRC4 inflammasome activation promotes Alzheimer's disease progression. *Animal Model Exp Med*. Mar 23 2024;doi:10.1002/ame2.12361
92. Du Y, Guo T, Hao Y, et al. PKCdelta serves as a potential biomarker and therapeutic target for microglia-mediated neuroinflammation in Alzheimer's disease. *Alzheimers Dement*. Aug 2024;20(8):5511–5527. doi:10.1002/alz.14047
93. Kumar A, Verma A, Chaurasia RN. Vitamin D and inflammatory cytokines association in mild cognitive impaired subjects. *Neurosci Lett*. 2023;795:137044. doi:10.1016/j.neulet.2022.137044
94. Tsang RSM, Sachdev PS, Reppermund S, et al. Sydney Memory and Ageing Study: An epidemiological cohort study of brain ageing and dementia. Article. *International Review of Psychiatry*. Dec 2013;25(6):711–725. doi:10.3109/09540261.2013.860890
95. King E, O'Brien JT, Donaghy P, et al. Peripheral inflammation in mild cognitive impairment with possible and probable Lewy body disease and Alzheimer's disease. *Int Psychogeriatr*. Apr 2019;31(4):551–560. doi:10.1017/S1041610218001126
96. Delaby C, Gabelle A, Blum D, et al. Central nervous system and peripheral inflammatory processes in Alzheimer's disease: biomarker profiling approach. Article. *Frontiers in Neurology*. Aug 2015;6:11. 181. doi:10.3389/fneur.2015.00181
97. Bermejo P, Martín-Aragón S, Benedí J, et al. Differences of peripheral inflammatory markers between mild cognitive impairment and Alzheimer's disease. *Immunol Lett*. 2008;117(2):198–202. doi:10.1016/j.imlet.2008.02.002
98. Dukic L, Simundic AM, Martinic-Popovic I, et al. The role of human kallikrein 6, clusterin and adiponectin as potential blood biomarkers of dementia. Article. *Clinical Biochemistry*. Feb 2016;49(3):213–218. doi:10.1016/j.clinbiochem.2015.10.014
99. Magalhaes TNC, Weiler M, Teixeira CVL, et al. Systemic Inflammation and Multimodal Biomarkers in Amnestic Mild Cognitive Impairment and Alzheimer's Disease. Article. *Molecular Neurobiology*. Jul 2018;55(7):5689–5697. doi:10.1007/s12035-017-0795-9
100. Chua XY, Chai YL, Chew WS, et al. Immunomodulatory sphingosine-1-phosphates as plasma biomarkers of Alzheimer's disease and vascular cognitive impairment. Article. *Alzheimers Research & Therapy*. Sep 2020;12(1):12. 122. doi:10.1186/s13195-020-00694-3
101. Garcia-Garcia I, Kamal F, Donica O, Dadar M, Alzheimer's Disease Neuroimaging I. Plasma levels of adipokines and insulin are associated with markers of brain atrophy and cognitive decline in the spectrum of Alzheimer's Disease. *Prog Neuropsychopharmacol Biol Psychiatry*. Aug 30 2024;134:111077. doi:10.1016/j.pnpbp.2024.111077
102. Deniz K, Ho CCG, Malphrus KG, et al. Plasma Biomarkers of Alzheimer's Disease in African Americans. Article. *Journal of Alzheimers Disease*. 2021;79(1):323–334. doi:10.3233/jad-200828

103. Costantini E, Carrarini C, Calisi D, et al. Search in the Periphery for Potential Inflammatory Biomarkers of Dementia with Lewy Bodies and Alzheimer's Disease. *J Alzheimers Dis*. 2024;99(3):1147–1158. doi:10.3233/jad-231471
104. Gertje EC, Janelidze S, van Westen D, et al. Associations Between CSF Markers of Inflammation, White Matter Lesions, and Cognitive Decline in Individuals Without Dementia. *Neurology*. 2023;100(17):e1812–e1824. doi:10.1212/WNL.0000000000207113
105. Kilic U, Elibol B, Uysal O, et al. Specific alterations in the circulating levels of the SIRT1, TLR4, and IL7 proteins in patients with dementia. *Exp Gerontol*. 2018;111:203–209. doi:10.1016/j.exger.2018.07.018
106. Correa JD, Starling D, Teixeira AL, Caramelli P, Silva TA. Chemokines in CSF of Alzheimer's disease patients. Article. *Arquivos De Neuro-Psiquiatria*. Jun 2011;69(3):455–459. doi:10.1590/s0004-282x2011000400009
107. Alsadany MA, Shehata HH, Mohamad MI, Mahfouz RG. Histone deacetylases enzyme, copper, and IL-8 levels in patients with Alzheimer's disease. *Am J Alzheimers Dis Other Demen*. 2013;28(1):54–61. doi:10.1177/1533317512467680
108. Kim SM, Song J, Kim S, et al. Identification of peripheral inflammatory markers between normal control and Alzheimer's disease. *BMC Neurol*. 2011;11:51. doi:10.1186/1471-2377-11-51
109. Popp J, Oikonomidi A, Tautvydaite D, et al. Markers of neuroinflammation associated with Alzheimer ' s disease pathology in older adults. Article. *Brain Behavior and Immunity*. May 2017;62:203–211. doi:10.1016/j.bbi.2017.01.020
110. Motta M, Imbesi R, Di Rosa M, Stivala F, Malaguarnera L. Altered plasma cytokine levels in Alzheimer's disease: Correlation with the disease progression. Article. *Immunology Letters*. Nov 2007;114(1):46–51. doi:10.1016/j.imlet.2007.09.002
111. Johansson P, Almqvist EG, Wallin A, et al. Reduced cerebrospinal fluid concentration of interleukin-12/23 subunit p40 in patients with cognitive impairment. *PLoS One*. 2017;12(5):e0176760. doi:10.1371/journal.pone.0176760
112. Malaguarnera L, Motta M, Di Rosa M, Anzaldi M, Malaguarnera M. Interleukin-18 and transforming growth factor-beta 1 plasma levels in Alzheimer's disease and vascular dementia. *Neuropathology*. 2006;26(4):307–12. doi:10.1111/j.1440-1789.2006.00701.x
113. Schipke CG, Gunter O, Weinert C, et al. Definition and quantification of six immune- and neuroregulatory serum proteins in healthy and demented elderly. Article. *Neurodegenerative Disease Management*. Aug 2019;9(4):193–203. doi:10.2217/nmt-2019-0003
114. Rosen C, Andersson CH, Andreasson U, et al. Increased Levels of Chitotriosidase and YKL-40 in Cerebrospinal Fluid from Patients with Alzheimer's Disease. Article. *Dementia and Geriatric Cognitive Disorders Extra*. 2014;4(2):297–304. doi:10.1159/000362164
115. Lee WJ, Liao YC, Wang YF, Lin IF, Wang SJ, Fuh JL. Plasma MCP-1 and Cognitive Decline in Patients with Alzheimer's Disease and Mild Cognitive Impairment: A Two-year Follow-up Study. *Sci Rep*. 2018;8(1):1280. doi:10.1038/s41598-018-19807-y
116. Oikonomidi A, Tautvydaite D, Gholamrezaee MM, Henry H, Bacher M, Popp J. Macrophage Migration Inhibitory Factor is Associated with Biomarkers of Alzheimer's Disease Pathology and Predicts Cognitive Decline in Mild Cognitive Impairment and Mild Dementia. Article. *Journal of Alzheimers Disease*. 2017;60(1):273–281. doi:10.3233/jad-170335
117. Zhang S, Zhao J, Zhang Y, Cai F, Wang L, Song W. Upregulation of MIF as a defense mechanism and a biomarker of Alzheimer's disease. *Alzheimers Res Ther*. 2019;11(1):54. doi:10.1186/s13195-019-0508-x
118. Hok AHYS, Del Campo M, Boiten WA, et al. Neuroinflammatory CSF biomarkers MIF, sTREM1, and sTREM2 show dynamic expression profiles in Alzheimer's disease. *J Neuroinflammation*. 2023;20(1):107. doi:10.1186/s12974-023-02796-9
119. Rosenberg GA, Prestopnik J, Knoefel J, et al. A Multimodal Approach to Stratification of Patients with Dementia: Selection of Mixed Dementia Patients Prior to Autopsy. Article. *Brain Sciences*. Aug 2019;9(8):10. 187. doi:10.3390/brainsci9080187

120. Hou Z, Sun A, Li Y, et al. What Are the Reliable Plasma Biomarkers for Mild Cognitive Impairment? A Clinical 4D Proteomics Study and Validation. *Mediators Inflamm*. 2024;2024:7709277. doi:10.1155/2024/7709277
121. Comi C, Carecchio M, Chiocchetti A, et al. Osteopontin is Increased in the Cerebrospinal Fluid of Patients with Alzheimer's Disease and Its Levels Correlate with Cognitive Decline. Article. *Journal of Alzheimers Disease*. 2010;19(4):1143–1148. doi:10.3233/jad-2010-1309
122. Sun Y, Yin XS, Guo H, Han RK, He RD, Chi LJ. Elevated Osteopontin Levels in Mild Cognitive Impairment and Alzheimer's Disease. Article. *Mediators of Inflammation*. 2013;2013:9. 615745. doi:10.1155/2013/615745
123. Laske C, Schmohl M, Leyhe T, et al. Immune Profiling in Blood Identifies sTNF-R1 Performing Comparably Well as Biomarker Panels for Classification of Alzheimer's Disease Patients. Article. *Journal of Alzheimers Disease*. 2013;34(2):367–375. doi:10.3233/jad-121558
124. Pillai JA, Khrestian M, Bena J, Leverenz JB, Bekris LM, Alzheimer's Dis Neuroimaging I. Temporal Ordering of Inflammatory Analytes sTNFR2 and sTREM2 in Relation to Alzheimer's Disease Biomarkers and Clinical Outcomes. Article. *Frontiers in Aging Neuroscience*. Jun 2021;13:14. 676744. doi:10.3389/fnagi.2021.676744
125. Boza-Serrano A, Vrillon A, Minta K, et al. Galectin-3 is elevated in CSF and is associated with A beta deposits and tau aggregates in brain tissue in Alzheimer's disease. Article. *Acta Neuropathologica*. Nov 2022;144(5):843–859. doi:10.1007/s00401-022-02469-6
126. Choi H, Choi Y, Lee EJ, et al. Hippocampal glucose uptake as a surrogate of metabolic change of microglia in Alzheimer's disease. *J Neuroinflammation*. 2021;18(1):190. doi:10.1186/s12974-021-02244-6
127. Li TR, Lyu DY, Liu FQ, Alzheimers Dis Neuroimaging I. Cerebrospinal Fluid sTREM2 in Alzheimer's Disease Is Associated with Both Amyloid and Tau Pathologies but not with Cognitive Status. Article. *Journal of Alzheimers Disease*. 2022;90(3):1123–1138. doi:10.3233/jad-220598
128. La Rosa F, Agostini S, Piancone F, et al. TREM2 Expression and Amyloid-Beta Phagocytosis in Alzheimer's Disease. *Int J Mol Sci*. 2023;24(10)doi:10.3390/ijms24108626
129. Gispert JD, Suárez-Calvet M, Monté GC, et al. Cerebrospinal fluid sTREM2 levels are associated with gray matter volume increases and reduced diffusivity in early Alzheimer's disease. *Alzheimers Dement*. 2016;12(12):1259–1272. doi:10.1016/j.jalz.2016.06.005
130. Mocali A, Cedrola S, Della Malva N, et al. Increased plasma levels of soluble CD40, together with the decrease of TGF beta 1, as possible differential markers of Alzheimer disease. *Exp Gerontol*. 2004;39(10):1555–61. doi:10.1016/j.exger.2004.07.007
131. Contreras JA, Aslanyan V, Sweeney MD, et al. Functional connectivity among brain regions affected in Alzheimer's disease is associated with CSF TNF-alpha in APOE4 carriers. Article. *Neurobiology of Aging*. Feb 2020;86:112–122. doi:10.1016/j.neurobiolaging.2019.10.013
132. Ferretti G, Serafini S, Angiolillo A, Monterosso P, Di Costanzo A, Matrone C. Advances in peripheral blood biomarkers of patients with Alzheimer's disease: Moving closer to personalized therapies. *Biomed Pharmacother*. 2023;165:115094. doi:10.1016/j.biopha.2023.115094
133. Serafini S, Ferretti G, Monterosso P, Angiolillo A, Di Costanzo A, Matrone C. TNF-alpha Levels Are Increased in Patients with Subjective Cognitive Impairment and Are Negatively Correlated with beta Amyloid-42. *Antioxidants (Basel)*. Feb 8 2024;13(2)doi:10.3390/antiox13020216
134. Kara SP, Altunan B, Unal A. Investigation of the peripheral inflammation (neutrophil-lymphocyte ratio) in two neurodegenerative diseases of the central nervous system. *Neurol Sci*. Mar 2022;43(3):1799–1807. doi:10.1007/s10072-021-05507-5
135. Li JQ, Zhang YR, Wang HF, et al. Exploring the links among peripheral immunity, biomarkers, cognition, and neuroimaging in Alzheimer's disease. *Alzheimers Dement (Amst)*. 2023;15(4):e12517. doi:10.1002/dad2.12517

136. Cervellati C, Pedrini D, Pirro P, et al. Neutrophil-Lymphocytes Ratio as Potential Early Marker for Alzheimer's Disease. *Mediators Inflamm*. 2024;2024:6640130. doi:10.1155/2024/6640130
137. Rembach A, Watt AD, Wilson WJ, et al. An increased neutrophil-lymphocyte ratio in Alzheimer's disease is a function of age and is weakly correlated with neocortical amyloid accumulation. *J Neuroimmunol*. 2014;273(1-2):65 – 71. doi:10.1016/j.jneuroim.2014.05.005
138. Schjønning Nielsen M, Simonsen AH, Siersma V, Hasselbalch SG, Høgh P. Are CSF Biomarkers Useful as Prognostic Indicators in Diagnostically Unresolved Cognitively Impaired Patients in a Normal Clinical Setting. *Dement Geriatr Cogn Dis Extra*. 2016;6(3):465–476. doi:10.1159/000449410
139. Mehta NH, Zhou L, Li Y, et al. Peripheral immune cell imbalance is associated with cortical beta-amyloid deposition and longitudinal cognitive decline. *Sci Rep*. 2023;13(1):8847. doi:10.1038/s41598-023-34012-2
140. Unda SR, Antoniazzi AM, Altschul DJ, Marongiu R. Peripheral Leukocytosis Predicts Cognitive Decline but Not Behavioral Disturbances: A Nationwide Study of Alzheimer's and Parkinson's Disease Patients. Article. *Dementia and Geriatric Cognitive Disorders*. Aug 2021;50(2):143–152. doi:10.1159/000516340
141. Craig-Schapiro R, Perrin RJ, Roe CM, et al. YKL-40: A Novel Prognostic Fluid Biomarker for Preclinical Alzheimer's Disease. Article. *Biological Psychiatry*. Nov 2010;68(10):903–912. doi:10.1016/j.biopsych.2010.08.025
142. Baldacci F, Toschi N, Lista S, et al. Two-level diagnostic classification using cerebrospinal fluid YKL-40 in Alzheimer's disease. Article. *Alzheimers & Dementia*. Sep 2017;13(9):993–1003. doi:10.1016/j.jalz.2017.01.021
143. Antonell A, Tort-Merino A, Ríos J, et al. Synaptic, axonal damage and inflammatory cerebrospinal fluid biomarkers in neurodegenerative dementias. *Alzheimers Dement*. 2020;16(2):262–272. doi:10.1016/j.jalz.2019.09.001
144. Janelidze S, Hertze J, Zetterberg H, et al. Cerebrospinal fluid neurogranin and YKL-40 as biomarkers of Alzheimer's disease. Article. *Annals of Clinical and Translational Neurology*. Jan 2016;3(1):12–20. doi:10.1002/acn3.266
145. Wang L, Gao T, Cai T, Li K, Zheng P, Liu J. Cerebrospinal fluid levels of YKL-40 in prodromal Alzheimer's disease. *Neurosci Lett*. 2020;715:134658. doi:10.1016/j.neulet.2019.134658
146. De Kort AM, Kuiperij HB, Alcolea D, et al. Cerebrospinal fluid levels of the neurotrophic factor neuroleukin are increased in early Alzheimer's disease, but not in cerebral amyloid angiopathy. Article. *Alzheimers Research & Therapy*. Sep 2021;13(1):10. 160. doi:10.1186/s13195-021-00899-0
147. Lleo A, Alcolea D, Martinez-Lage P, et al. Longitudinal cerebrospinal fluid biomarker trajectories along the Alzheimer's disease continuum in the BIOMARKAPD study. Article. *Alzheimers & Dementia*. Jun 2019;15(6):742–753. doi:10.1016/j.jalz.2019.01.015
148. Swanson A, Willette AA. Neuronal Pentraxin 2 predicts medial temporal atrophy and memory decline across the Alzheimer's disease spectrum. *Brain Behav Immun*. 2016;58:201–208. doi:10.1016/j.bbi.2016.07.148
149. Alcolea D, Carmona-Iragui M, Suárez-Calvet M, et al. Relationship between β-Secretase, inflammation and core cerebrospinal fluid biomarkers for Alzheimer's disease. *J Alzheimers Dis*. 2014;42(1):157–67. doi:10.3233/JAD-140240
150. Olsson B, Hertze J, Lautner R, et al. Microglial Markers are Elevated in the Prodromal Phase of Alzheimer's Disease and Vascular Dementia. Article. *Journal of Alzheimers Disease*. 2013;33(1):45–53. doi:10.3233/jad-2012-120787
151. Kester MI, Teunissen CE, Sutphen C, et al. Cerebrospinal fluid VILIP-1 and YKL-40, candidate biomarkers to diagnose, predict and monitor Alzheimer's disease in a memory clinic cohort. *Alzheimers Res Ther*. 2015;7(1):59. doi:10.1186/s13195-015-0142-1
152. Gispert JD, Monté GC, Falcon C, et al. CSF YKL-40 and pTau181 are related to different cerebral morphometric patterns in early AD. *Neurobiol Aging*. 2016;38:47–55. doi:10.1016/j.neurobiolaging.2015.10.022

153. Alcolea D, Martinez-Lage P, Sanchez-Juan P, et al. Amyloid precursor protein metabolism and inflammation markers in preclinical Alzheimer disease. Article. *Neurology*. Aug 2015;85(7):626–633. doi:10.1212/wnl.0000000000001859
154. Seyfried NT, Dammer EB, Swarup V, et al. A Multi-network Approach Identifies Protein-Specific Co-expression in Asymptomatic and Symptomatic Alzheimer's Disease. Article. *Cell Systems*. Jan 2017;4(1):60–+. doi:10.1016/j.cels.2016.11.006
155. Ayton S, Hall S, Janelidze S, et al. The Neuroinflammatory Acute Phase Response in Parkinsonian-Related Disorders. *Mov Disord*. May 2022;37(5):993–1003. doi:10.1002/mds.28958
156. Wang M, Liu Z, Du J, et al. Evaluation of Peripheral Immune Activation in Amyotrophic Lateral Sclerosis. *Front Neurol*. 2021;12:628710. doi:10.3389/fneur.2021.628710
157. Yuan X, Wan L, Chen Z, et al. Peripheral Inflammatory and Immune Landscape in Multiple System Atrophy: A Cross-Sectional Study. *Mov Disord*. Feb 2024;39(2):391–399. doi:10.1002/mds.29674
158. Chen D, Wei X, Zou J, et al. Contra-Directional Expression of Serum Homocysteine and Uric Acid as Important Biomarkers of Multiple System Atrophy Severity: A Cross-Sectional Study. *Front Cell Neurosci*. 2015;9:247. doi:10.3389/fncel.2015.00247
159. Eidson LN, Kannarkat GT, Barnum CJ, et al. Candidate inflammatory biomarkers display unique relationships with alpha-synuclein and correlate with measures of disease severity in subjects with Parkinson's disease. *J Neuroinflammation*. Aug 18 2017;14(1):164. doi:10.1186/s12974-017-0935-1
160. Nissen SK, Ferreira SA, Nielsen MC, et al. Soluble CD163 Changes Indicate Monocyte Association With Cognitive Deficits in Parkinson's Disease. *Mov Disord*. Apr 2021;36(4):963–976. doi:10.1002/mds.28424
161. Ohmichi T, Kasai T, Shinomoto M, et al. Serum leucine-rich α2 glycoprotein as a potential biomarker for systemic inflammation in Parkinson's disease. *PLoS One*. 2023;18(2):e0282153. doi:10.1371/journal.pone.0282153
162. Scott KM, Chong YT, Park S, et al. B lymphocyte responses in Parkinson's disease and their possible significance in disease progression. *Brain Commun*. 2023;5(2):fcad060. doi:10.1093/braincomms/fcad060
163. Bekris LM, Khrestian M, Dyne E, et al. Soluble TREM2 and biomarkers of central and peripheral inflammation in neurodegenerative disease. *J Neuroimmunol*. Jun 15 2018;319:19–27. doi:10.1016/j.jneuroim.2018.03.003
164. Borsche M, König IR, Delcambre S, et al. Mitochondrial damage-associated inflammation highlights biomarkers in PRKN/PINK1 parkinsonism. *Brain*. Oct 1 2020;143(10):3041–3051. doi:10.1093/brain/awaa246
165. Chen H, O'Reilly EJ, Schwarzschild MA, Ascherio A. Peripheral inflammatory biomarkers and risk of Parkinson's disease. *Am J Epidemiol*. Jan 1 2008;167(1):90–5. doi:10.1093/aje/kwm260
166. Martin-Ruiz C, Williams-Gray CH, Yarnall AJ, et al. Senescence and Inflammatory Markers for Predicting Clinical Progression in Parkinson's Disease: The ICICLE-PD Study. *J Parkinsons Dis*. 2020;10(1):193–206. doi:10.3233/jpd-191724
167. Mollenhauer B, Zimmermann J, Sixel-Döring F, et al. Baseline predictors for progression 4 years after Parkinson's disease diagnosis in the De Novo Parkinson Cohort (DeNoPa). *Mov Disord*. Jan 2019;34(1):67–77. doi:10.1002/mds.27492
168. Kim R, Kim HJ, Kim A, et al. Peripheral blood inflammatory markers in early Parkinson's disease. *J Clin Neurosci*. Dec 2018;58:30–33. doi:10.1016/j.jocn.2018.10.079
169. Santos-García D, de Deus Fonticoba T, Suárez Castro E, et al. High ultrasensitive serum C-reactive protein may be related to freezing of gait in Parkinson's disease patients. *J Neural Transm (Vienna)*. Dec 2019;126(12):1599–1608. doi:10.1007/s00702-019-02096-8
170. Imarisio A, Pilotto A, Garrafa E, et al. Plasma Cystatin C Correlates with Plasma NfL Levels and Predicts Disease Progression in Parkinson's Disease. *Neurodegener Dis*. 2021;21(5-6):109–116. doi:10.1159/000523982

171. King E, O'Brien J, Donaghy P, et al. Inflammation in mild cognitive impairment due to Parkinson's disease, Lewy body disease, and Alzheimer's disease. *Int J Geriatr Psychiatry*. 2019;34(8):1244–1250. doi:10.1002/gps.5124
172. Hall S, Janelidze S, Surova Y, Widner H, Zetterberg H, Hansson O. Cerebrospinal fluid concentrations of inflammatory markers in Parkinson's disease and atypical parkinsonian disorders. *Sci Rep*. Sep 5 2018;8(1):13276. doi:10.1038/s41598-018-31517-z
173. Heilman PL, Wang EW, Lewis MM, et al. Tryptophan Metabolites Are Associated With Symptoms and Nigral Pathology in Parkinson's Disease. *Mov Disord*. Nov 2020;35(11):2028–2037. doi:10.1002/mds.28202
174. Picca A, Guerra F, Calvani R, et al. Mitochondrial Signatures in Circulating Extracellular Vesicles of Older Adults with Parkinson's Disease: Results from the EXosomes in PArkiNson's Disease (EXPAND) Study. *J Clin Med*. Feb 12 2020;9(2)doi:10.3390/jcm9020504
175. Solmaz V, Pekdaş Genç E, Aksoy D, Çevik B, Kurt SG, Benli İ. Serum neutrophil-lymphocyte ratios, C-reactive protein and sedimentation levels in Parkinson's disease. *Cukurova Medical Journal*. 2018;43(2):305–311. doi:10.17826/cumj.341649
176. Yazar T, Yazar H. Evaluation of C-reactive Protein/Albumin Ratio According to Stage in Patients with Idiopathic Parkinson Disease. *Turkish Journal Of Neurology*. 09/23 2019;25:123–128. doi:10.4274/tnd.galenos.2019.13334
177. Wang LX, Liu C, Shao YQ, Jin H, Mao CJ, Chen J. Peripheral blood inflammatory cytokines are associated with rapid eye movement sleep behavior disorder in Parkinson's disease. *Neurosci Lett*. Jun 21 2022;782:136692. doi:10.1016/j.neulet.2022.136692
178. Baran A, Bulut M, Kaya MC, et al. High-sensitivity C-reactive protein and high mobility group box-1 levels in Parkinson's disease. *Neurol Sci*. Jan 2019;40(1):167–173. doi:10.1007/s10072-018-3611-z
179. Song IU, Chung SW, Kim JS, Lee KS. Association between high-sensitivity C-reactive protein and risk of early idiopathic Parkinson's disease. *Neurol Sci*. Feb 2011;32(1):31–4. doi:10.1007/s10072-010-0335-0
180. Song IU, Kim YD, Cho HJ, Chung SW. Is neuroinflammation involved in the development of dementia in patients with Parkinson's disease? *Intern Med*. 2013;52(16):1787–92. doi:10.2169/internalmedicine.52.0474
181. Jin H, Gu HY, Mao CJ, Chen J, Liu CF. Association of inflammatory factors and aging in Parkinson's disease. *Neurosci Lett*. Sep 25 2020;736:135259. doi:10.1016/j.neulet.2020.135259
182. Andican G, Konukoglu D, Bozluolcay M, Bayülkem K, Firtiına S, Burcak G. Plasma oxidative and inflammatory markers in patients with idiopathic Parkinson's disease. *Acta Neurol Belg*. Jun 2012;112(2):155–9. doi:10.1007/s13760-012-0015-3
183. Scalzo P, de Miranda AS, Guerra Amaral DC, de Carvalho Vilela M, Cardoso F, Teixeira AL. Serum levels of chemokines in Parkinson's disease. *Neuroimmunomodulation*. 2011;18(4):240–4. doi:10.1159/000323779
184. Majbour NK, Aasly JO, Hustad E, et al. CSF total and oligomeric α-Synuclein along with TNF-α as risk biomarkers for Parkinson's disease: a study in LRRK2 mutation carriers. *Transl Neurodegener*. May 6 2020;9(1):15. doi:10.1186/s40035-020-00192-4
185. Gupta M, Paliwal VK, Babu GN. Serum fractalkine and 3-nitrotyrosine levels correlate with disease severity in Parkinson's disease: a pilot study. *Metab Brain Dis*. Jan 2022;37(1):209–217. doi:10.1007/s11011-021-00801-9
186. Li Y, Yang Y, Zhao A, et al. Parkinson's disease peripheral immune biomarker profile: a multicentre, cross-sectional and longitudinal study. *J Neuroinflammation*. May 24 2022;19(1):116. doi:10.1186/s12974-022-02481-3
187. Dzamko N, Rowe DB, Halliday GM. Increased peripheral inflammation in asymptomatic leucine-rich repeat kinase 2 mutation carriers. *Mov Disord*. Jun 2016;31(6):889–97. doi:10.1002/mds.26529
188. Lerche S, Zimmermann M, Roeben B, et al. Inflammatory CSF profiles and longitudinal development of cognitive decline in sporadic and GBA-associated PD. *NPJ Parkinsons Dis*. Mar 11 2023;9(1):38. doi:10.1038/s41531-023-00476-2

189. Thaler A, Omer N, Giladi N, et al. Mutations in GBA and LRRK2 Are Not Associated with Increased Inflammatory Markers. *J Parkinsons Dis*. 2021;11(3):1285–1296. doi:10.3233/jpd-212624
190. Liu SY, Qiao HW, Song TB, et al. Brain microglia activation and peripheral adaptive immunity in Parkinson's disease: a multimodal PET study. *J Neuroinflammation*. Aug 29 2022;19(1):209. doi:10.1186/s12974-022-02574-z
191. Yacoubian TA, Fang YD, Gerstenecker A, et al. Brain and Systemic Inflammation in De Novo Parkinson's Disease. *Mov Disord*. May 2023;38(5):743–754. doi:10.1002/mds.29363
192. Rathnayake D, Chang T, Udagama P. Selected serum cytokines and nitric oxide as potential multi-marker biosignature panels for Parkinson disease of varying durations: a case-control study. *BMC Neurol*. Apr 6 2019;19(1):56. doi:10.1186/s12883-019-1286-6
193. Gruden MA, Yanamandra K, Kucheryanu VG, et al. Correlation between protective immunity to α-synuclein aggregates, oxidative stress and inflammation. *Neuroimmunomodulation*. 2012;19(6):334–342. doi:10.1159/000341400
194. Santoro M, Maetzler W, Stathakos P, et al. In-vivo evidence that high mobility group box 1 exerts deleterious effects in the 1-methyl-4-phenyl-1,2,3,6-tetrahydropyridine model and Parkinson's disease which can be attenuated by glycyrrhizin. *Neurobiol Dis*. Jul 2016;91:59–68. doi:10.1016/j.nbd.2016.02.018
195. Yazdani S, Mariosa D, Hammar N, et al. Peripheral immune biomarkers and neurodegenerative diseases: A prospective cohort study with 20 years of follow-up. *Ann Neurol*. Dec 2019;86(6):913–926. doi:10.1002/ana.25614
196. Yang Y, Han C, Guo L, Guan Q. High expression of the HMGB1-TLR4 axis and its downstream signaling factors in patients with Parkinson's disease and the relationship of pathological staging. *Brain Behav*. Apr 2018;8(4):e00948. doi:10.1002/brb3.948
197. Kıçık A, Tüzün E, Erdoğdu E, et al. Neuroinflammation Mediators are Reduced in Sera of Parkinson's Disease Patients with Mild Cognitive Impairment. *Noro Psikiyatr Ars*. Mar 2020;57(1):15–17. doi:10.29399/npa.23624
198. Yu SY, Zuo LJ, Wang F, et al. Potential biomarkers relating pathological proteins, neuroinflammatory factors and free radicals in PD patients with cognitive impairment: a cross-sectional study. *BMC Neurol*. May 22 2014;14:113. doi:10.1186/1471-2377-14-113
199. Roy A, Banerjee R, Choudhury S, et al. Novel inflammasome and oxidative modulators in Parkinson's disease: A prospective study. *Neurosci Lett*. Aug 24 2022;786:136768. doi:10.1016/j.neulet.2022.136768
200. Sarkar S, Roy A, Choudhury S, Banerjee R, Dey S, Kumar H. Levodopa-induced Dyskinesia in Parkinson's Disease: Plausible Inflammatory and Oxidative Stress Biomarkers. *Can J Neurol Sci*. Jan 2024;51(1):104–109. doi:10.1017/cjn.2023.8
201. Xu J, He X, Xu Y, et al. Characteristics of systemic inflammation and brain iron deposition in Parkinson's disease patients. *Ann Clin Transl Neurol*. Mar 2022;9(3):276–285. doi:10.1002/acn3.51512
202. Karpenko MN, Vasilishina AA, Gromova EA, Muruzheva ZM, Miliukhina IV, Bernadotte A. Interleukin-1β, interleukin-1 receptor antagonist, interleukin-6, interleukin-10, and tumor necrosis factor-α levels in CSF and serum in relation to the clinical diversity of Parkinson's disease. *Cell Immunol*. May 2018;327:77–82. doi:10.1016/j.cellimm.2018.02.011
203. Hu Y, Yu SY, Zuo LJ, et al. Parkinson disease with REM sleep behavior disorder: features, α-synuclein, and inflammation. *Neurology*. Mar 3 2015;84(9):888–94. doi:10.1212/wnl.0000000000001308
204. Li T, Tan X, Tian L, et al. The role of Nurr1-miR-30e-5p-NLRP3 axis in inflammation-mediated neurodegeneration: insights from mouse models and patients' studies in Parkinson's disease. *J Neuroinflammation*. Nov 22 2023;20(1):274. doi:10.1186/s12974-023-02956-x
205. Fan Z, Pan YT, Zhang ZY, et al. Systemic activation of NLRP3 inflammasome and plasma α-synuclein levels are correlated with motor severity and progression in Parkinson's disease. *J Neuroinflammation*. Jan 8 2020;17(1):11. doi:10.1186/s12974-019-1670-6

206. Calvani R, Picca A, Landi G, et al. A novel multi-marker discovery approach identifies new serum biomarkers for Parkinson's disease in older people: an EXosomes in PArkiNson Disease (EXPAND) ancillary study. *Geroscience*. Oct 2020;42(5):1323–1334. doi:10.1007/s11357-020-00192-2
207. Csencsits-Smith K, Suescun J, Li K, Luo S, Bick DL, Schiess M. Serum Lymphocyte-Associated Cytokine Concentrations Change More Rapidly over Time in Multiple System Atrophy Compared to Parkinson Disease. *Neuroimmunomodulation*. 2016;23(5-6):301–308. doi:10.1159/000460297
208. Zhao X, Jin T, Zheng C, Ma D, Zhang Y. Imbalance of Circulating Tfh/Tfr Cells in Patients With Parkinson's Disease. *Front Neurol*. 2020;11:572205. doi:10.3389/fneur.2020.572205
209. Green HF, Khosousi S, Svenningsson P. Plasma IL-6 and IL-17A Correlate with Severity of Motor and Non-Motor Symptoms in Parkinson's Disease. *J Parkinsons Dis*. 2019;9(4):705–709. doi:10.3233/jpd-191699
210. Ton TG, Jain S, Biggs ML, et al. Markers of inflammation in prevalent and incident Parkinson's disease in the Cardiovascular Health Study. *Parkinsonism Relat Disord*. Mar 2012;18(3):274–8. doi:10.1016/j.parkreldis.2011.11.003
211. Martin-Bastida A, Tilley BS, Bansal S, Gentleman SM, Dexter DT, Ward RJ. Iron and inflammation: in vivo and post-mortem studies in Parkinson's disease. *J Neural Transm (Vienna)*. Jan 2021;128(1):15–25. doi:10.1007/s00702-020-02271-2
212. Roy A, Mondal B, Banerjee R, et al. Do peripheral immune and neurotrophic markers correlate with motor severity of Parkinson's disease? *J Neuroimmunol*. May 15 2021;354:577545. doi:10.1016/j.jneuroim.2021.577545
213. Wan Y, Zhao Y, Pan M, et al. Peripheral biomarkers of Parkinson's disease and its correlation with clinical symptoms: a case-control study. *BMC Neurol*. Oct 28 2024;24(1):417. doi:10.1186/s12883-024-03918-3
214. Santaella A, Kuiperij HB, van Rumund A, et al. Inflammation biomarker discovery in Parkinson's disease and atypical parkinsonisms. *BMC Neurol*. Jan 17 2020;20(1):26. doi:10.1186/s12883-020-1608-8
215. Wennstrom M, Surova Y, Hall S, et al. The Inflammatory Marker YKL-40 Is Elevated in Cerebrospinal Fluid from Patients with Alzheimer's but Not Parkinson's Disease or Dementia with Lewy Bodies. *PLoS One*. 2015;10(8):e0135458. doi:10.1371/journal.pone.0135458
216. Compta Y, Dias SP, Giraldo DM, et al. Cerebrospinal fluid cytokines in multiple system atrophy: A cross-sectional Catalan MSA registry study. *Parkinsonism Relat Disord*. Aug 2019;65:3–12. doi:10.1016/j.parkreldis.2019.05.040
217. Rentzos M, Nikolaou C, Andreadou E, et al. Circulating interleukin-15 and RANTES chemokine in Parkinson's disease. *Acta Neurol Scand*. Dec 2007;116(6):374–9. doi:10.1111/j.1600-0404.2007.00894.x
218. Cabrera Ranaldi E, Nuytemans K, Martinez A, Luca CC, Keane RW, de Rivero Vaccari JP. Proof-of-Principle Study of Inflammasome Signaling Proteins as Diagnostic Biomarkers of the Inflammatory Response in Parkinson's Disease. *Pharmaceuticals (Basel)*. Jun 15 2023;16(6)doi:10.3390/ph16060883
219. Yang L, Guo C, Zhu J, et al. Increased Levels of Pro-Inflammatory and Anti-Inflammatory Cellular Responses in Parkinson's Disease Patients: Search for a Disease Indicator. *Med Sci Monit*. Jun 18 2017;23:2972–2978. doi:10.12659/msm.904240
220. Ataç Uçar C, Gökçe Çokal B, Ünal Artık HA, İnan LE, Yoldaş TK. Comparison of neutrophil-lymphocyte ratio (NLR) in Parkinson's disease subtypes. *Neurol Sci*. Feb 2017;38(2):287–293. doi:10.1007/s10072-016-2758-8
221. Umehara T, Mimori M, Kokubu T, et al. Peripheral immune profile in drug-naive dementia with Lewy bodies. *J Neurol*. Jul 2024;271(7):4146–4157. doi:10.1007/s00415-024-12336-x
222. Grillo P, Sancesario GM, Bovenzi R, et al. Neutrophil-to-lymphocyte ratio and lymphocyte count reflect alterations in central neurodegeneration-associated proteins and clinical severity in Parkinson Disease patients. *Parkinsonism Relat Disord*. Jul 2023;112:105480. doi:10.1016/j.parkreldis.2023.105480

223. Li F, Weng G, Zhou H, et al. The neutrophil-to-lymphocyte ratio, lymphocyte-to-monocyte ratio, and neutrophil-to-high-density-lipoprotein ratio are correlated with the severity of Parkinson's disease. *Front Neurol*. 2024;15:1322228. doi:10.3389/fneur.2024.1322228
224. Giallluisi A, Tirozzi A, Costanzo S, et al. Blood-based biological ageing and red cell distribution width are associated with prevalent Parkinson's disease: findings from a large Italian population cohort. *Front Endocrinol (Lausanne)*. 2024;15:1376545. doi:10.3389/fendo.2024.1376545
225. Zhang Z, Wang Y, Wang J, et al. The role of peripheral inflammation-related biomarkers in distinguishing Parkinson's disease. *Parkinsonism Relat Disord*. Jun 2024;123:106102. doi:10.1016/j.parkreldis.2024.106102
226. Muñoz-Delgado L, Macías-García D, Periñán MT, et al. Peripheral inflammatory immune response differs among sporadic and familial Parkinson's disease. *NPJ Parkinsons Dis*. Jan 31 2023;9(1):12. doi:10.1038/s41531-023-00457-5
227. Munoz-Delgado L, Luque-Ambrosiani A, Zamora BB, et al. Peripheral immune profile and neutrophil-to-lymphocyte ratio in progressive supranuclear palsy: Case-control study and meta-analysis. *Eur J Neurol*. Dec 2024;31(12):e16451. doi:10.1111/ene.16451
228. Qin Y, Wei Y, Meng LQ, Chen WC. The role of red cell distribution width in patients with

Parkinson's disease. *International Journal of Clinical and Experimental Medicine*. 2016;9(3):6143–6147.
229. Bäckström D, Granåsen G, Mo SJ, et al. Prediction and early biomarkers of cognitive decline in Parkinson disease and atypical parkinsonism: a population-based study. *Brain Commun*. 2022;4(2):fcac040. doi:10.1093/braincomms/fcac040
230. Janelidze S, Hertze J, Zetterberg H, et al. Cerebrospinal fluid neurogranin and YKL-40 as biomarkers of Alzheimer's disease. *Ann Clin Transl Neurol*. Jan 2016;3(1):12–20. doi:10.1002/acn3.266
231. Gevezova M, Kazakova M, Trenova A, Sarafian V. YKL-40 and the Cellular Metabolic Profile in Parkinson's Disease. *Int J Mol Sci*. Nov 14 2023;24(22)doi:10.3390/ijms242216297
232. Brosseron F, Traschütz A, Widmann CN, et al. Characterization and clinical use of inflammatory cerebrospinal fluid protein markers in Alzheimer's disease. *Alzheimers Res Ther*. Feb 26 2018;10(1):25. doi:10.1186/s13195-018-0353-3
233. Roos P, von Essen MR, Nielsen TT, et al. Inflammatory markers of CHMP2B-mediated frontotemporal dementia. *J Neuroimmunol*. Nov 15 2018;324:136–142. doi:10.1016/j.jneuroim.2018.08.009
234. Katisko K, Solje E, Korhonen P, et al. Peripheral inflammatory markers and clinical correlations in patients with frontotemporal lobar degeneration with and without the C9orf72 repeat expansion. *J Neurol*. Jan 2020;267(1):76–86. doi:10.1007/s00415-019-09552-1
235. Chu M, Wen L, Jiang D, et al. Peripheral inflammation in behavioural variant frontotemporal dementia: associations with central degeneration and clinical measures. *Journal of Neuroinflammation*. 2023/03/08 2023;20(1):65. doi:10.1186/s12974-023-02746-5
236. Bossù P, Salani F, Alberici A, et al. Loss of function mutations in the progranulin gene are related to pro-inflammatory cytokine dysregulation in frontotemporal lobar degeneration patients. *Journal of Neuroinflammation*. 2011/06/06 2011;8(1):65. doi:10.1186/1742-2094-8-65
237. Asken BM, Ljubenkov PA, Staffaroni AM, et al. Plasma inflammation for predicting phenotypic conversion and clinical progression of autosomal dominant frontotemporal lobar degeneration. *J Neurol Neurosurg Psychiatry*. Jul 2023;94(7):541–549. doi:10.1136/jnnp-2022-330866
238. Baldacci F, Toschi N, Lista S, et al. Two-level diagnostic classification using cerebrospinal fluid YKL-40 in Alzheimer's disease. *Alzheimers Dement*. Sep 2017;13(9):993–1003. doi:10.1016/j.jalz.2017.01.021

239. Alcolea D, Irwin DJ, Illán-Gala I, et al. Elevated YKL-40 and low sAPPβ:YKL-40 ratio in antemortem cerebrospinal fluid of patients with pathologically confirmed FTLD. *J Neurol Neurosurg Psychiatry*. Feb 2019;90(2):180–186. doi:10.1136/jnnp-2018-318993
240. Alcolea D, Vilaplana E, Suárez-Calvet M, et al. CSF sAPPβ, YKL-40, and neurofilament light in frontotemporal lobar degeneration. *Neurology*. Jul 11 2017;89(2):178–188. doi:10.1212/wnl.0000000000004088
241. Woollacott IOC, Nicholas JM, Heller C, et al. Cerebrospinal Fluid YKL-40 and Chitotriosidase Levels in Frontotemporal Dementia Vary by Clinical, Genetic and Pathological Subtype. *Dement Geriatr Cogn Disord*. 2020;49(1):56–76. doi:10.1159/000506282
242. Thompson AG, Gray E, Bampton A, Raciborska D, Talbot K, Turner MR. CSF chitinase proteins in amyotrophic lateral sclerosis. *J Neurol Neurosurg Psychiatry*. Nov 2019;90(11):1215–1220. doi:10.1136/jnnp-2019-320442
243. Gaur N, Huss E, Prell T, et al. Monocyte-Derived Macrophages Contribute to Chitinase Dysregulation in Amyotrophic Lateral Sclerosis: A Pilot Study. *Front Neurol*. 2021;12:629332. doi:10.3389/fneur.2021.629332
244. Kläppe U, Sennfält S, Lovik A, et al. Neurodegenerative biomarkers outperform neuroinflammatory biomarkers in amyotrophic lateral sclerosis. *Amyotroph Lateral Scler Frontotemporal Degener*. Feb 2024;25(1-2):150–161. doi:10.1080/21678421.2023.2263874
245. Gille B, De Schaepdryver M, Dedeene L, et al. Inflammatory markers in cerebrospinal fluid: independent prognostic biomarkers in amyotrophic lateral sclerosis? *J Neurol Neurosurg Psychiatry*. Dec 2019;90(12):1338–1346. doi:10.1136/jnnp-2018-319586
246. Gray E, Thompson AG, Wuu J, et al. CSF chitinases before and after symptom onset in amyotrophic lateral sclerosis. *Ann Clin Transl Neurol*. Aug 2020;7(8):1296–1306. doi:10.1002/acn3.51114
247. Kjaeldgaard AL, Pilely K, Olsen KS, et al. Complement Profiles in Patients with Amyotrophic Lateral Sclerosis: A Prospective Observational Cohort Study. *J Inflamm Res*. 2021;14:1043–1053. doi:10.2147/JIR.S298307
248. Ganesalingam J, An J, Shaw CE, Shaw G, Lacomis D, Bowser R. Combination of neurofilament heavy chain and complement C3 as CSF biomarkers for ALS. *J Neurochem*. May 2011;117(3):528–37. doi:10.1111/j.1471-4159.2011.07224.x
249. Ju W, Ban JJ, Im HR, et al. Association of serum Spp1 levels with disease progression in ALS and SBMA. *Ann Clin Transl Neurol*. Jul 2024;11(7):1809–1818. doi:10.1002/acn3.52087
250. Beers DR, Zhao W, Neal DW, et al. Elevated acute phase proteins reflect peripheral inflammation and disease severity in patients with amyotrophic lateral sclerosis. *Sci Rep*. Sep 17 2020;10(1):15295. doi:10.1038/s41598-020-72247-5
251. Cao W, Cao Z, Tian Y, et al. Neutrophils Are Associated with Higher Risk of Incident Amyotrophic Lateral Sclerosis in a BMI- and Age-Dependent Manner. *Ann Neurol*. Nov 2023;94(5):942–954. doi:10.1002/ana.26760
252. Niccolai E, Pedone M, Martinelli I, et al. Amyotrophic lateral sclerosis stratification: unveiling patterns with virome, inflammation, and metabolism molecules. *J Neurol*. Jul 2024;271(7):4310–4325. doi:10.1007/s00415-024-12348-7
253. Mitchell RM, Freeman WM, Randazzo WT, et al. A CSF biomarker panel for identification of patients with amyotrophic lateral sclerosis. *Neurology*. Jan 6 2009;72(1):14–9. doi:10.1212/01.wnl.0000333251.36681.a5
254. Femiano C, Bruno A, Gilio L, et al. Inflammatory signature in amyotrophic lateral sclerosis predicting disease progression. *Sci Rep*. Aug 27 2024;14(1):19796. doi:10.1038/s41598-024-67165-9
255. Perner C, Perner F, Stubendorff B, et al. Dysregulation of chemokine receptor expression and function in leukocytes from ALS patients. *J Neuroinflammation*. Mar 28 2018;15(1):99. doi:10.1186/s12974-018-1135-3
256. Prado LGR, Rocha NP, de Souza LC, et al. Longitudinal assessment of clinical and inflammatory markers in patients with amyotrophic lateral sclerosis. *J Neurol Sci*. Nov 15 2018;394:69–74. doi:10.1016/j.jns.2018.08.033

257. Babu GN, Kumar A, Chandra R, Puri SK, Kalita J, Misra UK. Elevated inflammatory markers in a group of amyotrophic lateral sclerosis patients from northern India. *Neurochem Res*. Jun 2008;33(6):1145–9. doi:10.1007/s11064-007-9564-x
258. Jin M, Gunther R, Akgun K, Hermann A, Ziemssen T. Peripheral proinflammatory Th1/Th17 immune cell shift is linked to disease severity in amyotrophic lateral sclerosis. *Sci Rep*. Apr 3 2020;10(1):5941. doi:10.1038/s41598-020-62756-8
259. Tortelli R, Zecca C, Piccininni M, et al. Plasma Inflammatory Cytokines Are Elevated in ALS. *Front Neurol*. 2020;11:552295. doi:10.3389/fneur.2020.552295
260. Polverino A, Rucco R, Stillitano I, et al. In Amyotrophic Lateral Sclerosis Blood Cytokines Are Altered, but Do Not Correlate with Changes in Brain Topology. *Brain Connect*. Oct 2020;10(8):411–421. doi:10.1089/brain.2020.0741
261. Cao MC, Cawston EE, Chen G, et al. Serum biomarkers of neuroinflammation and blood-brain barrier leakage in amyotrophic lateral sclerosis. *BMC Neurol*. Jun 11 2022;22(1):216. doi:10.1186/s12883-022-02730-1
262. Pronto-Laborinho A, Pinto S, Gromicho M, Pereira M, Swash M, de Carvalho M. Interleukin-6 and amyotrophic lateral sclerosis. *J Neurol Sci*. Mar 15 2019;398:50–53. doi:10.1016/j.jns.2019.01.026
263. Blasco H, Garcon G, Patin F, et al. Panel of Oxidative Stress and Inflammatory Biomarkers in ALS: A Pilot Study. *Can J Neurol Sci*. Jan 2017;44(1):90–95. doi:10.1017/cjn.2016.284
264. Du Y, Zhao W, Thonhoff JR, Wang J, Wen S, Appel SH. Increased activation ability of monocytes from ALS patients. *Exp Neurol*. Jun 2020;328:113259. doi:10.1016/j.expneurol.2020.113259
265. Mitchell RM, Simmons Z, Beard JL, Stephens HE, Connor JR. Plasma biomarkers associated with ALS and their relationship to iron homeostasis. *Muscle Nerve*. Jul 2010;42(1):95–103. doi:10.1002/mus.21625
266. Baron P, Bussini S, Cardin V, et al. Production of monocyte chemoattractant protein-1 in amyotrophic lateral sclerosis. *Muscle Nerve*. Oct 2005;32(4):541–4. doi:10.1002/mus.20376
267. Yang X, Gao L, Wu X, Zhang Y, Zang D. Increased levels of MIP-1alpha in CSF and serum of ALS. *Acta Neurol Scand*. Aug 2016;134(2):94–100. doi:10.1111/ane.12513
268. Keizman D, Rogowski O, Berliner S, et al. Low-grade systemic inflammation in patients with amyotrophic lateral sclerosis. *Acta Neurol Scand*. Jun 2009;119(6):383–9. doi:10.1111/j.1600-0404.2008.01112.x
269. Rolfes L, Schulte-Mecklenbeck A, Schreiber S, et al. Amyotrophic lateral sclerosis patients show increased peripheral and intrathecal T-cell activation. *Brain Commun*. 2021;3(3):fcab157. doi:10.1093/braincomms/fcab157
270. Murdock BJ, Famie JP, Piecuch CE, et al. NK cells associate with ALS in a sex- and age-dependent manner. *JCI Insight*. Jun 8 2021;6(11)doi:10.1172/jci.insight.147129
271. Gentile F, Maranzano A, Verde F, et al. The value of routine blood work-up in clinical stratification and prognosis of patients with amyotrophic lateral sclerosis. *J Neurol*. Feb 2024;271(2):794–803. doi:10.1007/s00415-023-12015-3
272. Martinelli I, Zucchi E, Simonini C, et al. SerpinA1 levels in amyotrophic lateral sclerosis patients: An exploratory study. *Eur J Neurol*. Jan 2024;31(1):e16054. doi:10.1111/ene.16054
273. Andrés-Benito P, Domínguez R, Colomina MJ, Llorens F, Povedano M, Ferrer I. YKL40 in sporadic amyotrophic lateral sclerosis: cerebrospinal fluid levels as a prognosis marker of disease progression. *Aging (Albany NY)*. Sep 13 2018;10(9):2367–2382. doi:10.18632/aging.101551
274. Surendranathan A, Su L, Mak E, et al. Early microglial activation and peripheral inflammation in dementia with Lewy bodies. *Brain*. Dec 1 2018;141(12):3415–3427. doi:10.1093/brain/awy265
275. Amin J, Boche D, Clough Z, et al. Peripheral immunophenotype in dementia with Lewy bodies and Alzheimer's disease: an observational clinical study. *J Neurol Neurosurg Psychiatry*. Nov 2020;91(11):1219–1226. doi:10.1136/jnnp-2020-323603

276. Wennstrom M, Nielsen HM, Orhan F, Londos E, Minthon L, Erhardt S. Kynurenic Acid levels in cerebrospinal fluid from patients with Alzheimer's disease or dementia with lewy bodies. *Int J Tryptophan Res*. 2014;7:1–7. doi:10.4137/IJTR.S13958
277. Vrillon A, Bousiges O, Götze K, et al. Plasma biomarkers of amyloid, tau, axonal, and neuroinflammation pathologies in dementia with Lewy bodies. *Alzheimers Res Ther*. Jul 3 2024;16(1):146. doi:10.1186/s13195-024-01502-y

Search Strategy

| | | | |
|---|---|---|---|
| #3 | | | Search: **#1 AND #2** |
| #2 | | | Search: **"Neurodegenerative Diseases"[Mesh] OR "neurodegenerative disease*"[tw] OR "Alzheimer disease"[tw] OR "Huntington disease"[tw] OR "Huntington's Disease"[tw] OR "Parkinson disease"[tw] OR "Parkinson's disease"[tw] OR "motor neuron disease"[tw] OR "amyotrophic lateral sclerosis"[tw] OR "multiple system atrophy"[tw] OR "frontotemporal dementia"[tw] OR "progressive supranuclear palsy"[tw] OR "Neurologic Degenerative Disease*"[tw] OR "Degenerative Neurologic Disease*"[tw] OR "Neurodegenerative Disorder*"[tw] OR "Degenerative Neurologic Disorder*"[tw] OR "Alzheimer Disease"[Mesh] OR "Huntington Disease"[Mesh] OR "Parkinson Disease"[Mesh] OR "Amyotrophic Lateral Sclerosis"[Mesh] OR "Multiple System Atrophy"[Mesh] OR "Frontotemporal Dementia"[Mesh] OR "Supranuclear Palsy, Progressive"[Mesh]** |
| #1 | | | Search: **(("Inflammation"[Mesh] OR "Inflammation*"[tw]) AND ("Biomarkers"[Mesh] OR "biomarker*"[tw] OR "disease progression"[Mesh] OR "disease progression"[tw])) OR (("Inflammation"[Mesh] OR "Inflammation*"[tw]) AND ("Biomarkers"[Mesh] OR "biomarker*"[tw] OR "disease progression"[Mesh] OR "disease progression"[tw]) AND ("Blood"[Mesh] OR "Urine"[Mesh] OR "Cerebrospinal Fluid"[Mesh] OR "cerebrospinal fluid" [Subheading] OR "urine" [Subheading] OR "blood"[tw] OR "cerebrospinal fluid"[tw] OR "urine"[tw] OR "blood" [Subheading] OR "Cerebrospinal Fluids"[tw] OR "Cerebro Spinal Fluid"[tw] OR "Cerebro Spinal Fluids"[tw] OR "Neuroimaging"[Mesh] OR "Magnetic Resonance Imaging"[Mesh] OR "Positron-Emission Tomography"[Mesh] OR "Magnetic Resonance Spectroscopy"[Mesh] OR "Neuroimaging*"[tw] OR "Magnetic Resonance Imaging*"[tw] OR "Positron Emission Tomograph*"[tw] OR "Magnetic Resonance Spectroscop*"[tw]))** |

**Excluded papers based on biomarker threshold (measured in ≥ 3 independent cohorts)**

(Andrés-Benito et al. 2020; Bagheri et al. 2018; Bawa et al. 2020; Borrego-Écija et al. 2024; Bu et al. 2022; Carvalho et al. 2015; Comi et al. 2010; Crispoltoni et al. 2017; Eruysal et al. 2019; Festoff et al. 2016; Gao et al. 2023; Goldknopf et al. 2006; Helmfors et al. 2015; Horvath et al. 2016; Lattanzi et al. 2019; Liang et al. 2024; Ljubenkov et al. 2019; Lunetta et al. 2020; Masuda et al. 2017; Naude et al. 2021; Niemela et al. 2018; Petrozziello et al. 2020; Rocha et al. 2014; Rydbirk et al. 2022; Sun et al. 2014; Trolese et al. 2020; Watt et al. 2015; Wormser et al. 2016; Xia et al. 2024; Xiong et al. 2022; Zheng et al. 2022)


Andrés-Benito, Pol, Mònica Povedano, Raúl Domínguez, Carla Marco, Maria J. Colomina, Óscar López-Pérez, Isabel Santana, Inês Baldeiras, Sergio Martínez-Yelámos, Inga Zerr, Franc Llorens, Joaquín Fernández-Irigoyen, Enrique Santamaría, and Isidro Ferrer. 2020. 'Increased C-X-C Motif Chemokine Ligand 12 Levels in Cerebrospinal Fluid as a Candidate Biomarker in Sporadic Amyotrophic Lateral Sclerosis', *International Journal of Molecular Sciences*, 21: 8680.

Bagheri, Vahid, Hossein Khorramdelazad, Gholamhossein Hassanshahi, Amir Moghadam-Ahmadi, and Alireza Vakilian. 2018. 'CXCL12 and CXCR4 in the Peripheral Blood of Patients with Parkinson’s Disease', *Neuroimmunomodulation*, 25: 201–05.

Bawa, K. K., S. H. Krance, N. Herrmann, H. Cogo-Moreira, M. Ouk, D. Yu, C. Y. Wu, S. E. Black, K. L. Lanctot, W. Swardfager, and Initiative Alzheimer's Disease Neuroimaging. 2020. 'A peripheral neutrophil-related inflammatory factor predicts a decline in executive function in mild Alzheimer's disease', *J Neuroinflammation*, 17: 84.

Borrego-Écija, S., A. Pérez-Millan, A. Antonell, L. Fort-Aznar, E. Kaya-Tilki, A. León-Halcón, A. Lladó, L. Molina-Porcel, M. Balasa, J. Juncà-Parella, J. Vitorica, J. L. Venero, T. Deierborg, A. Boza-Serrano, and R. Sánchez-Valle. 2024. 'Galectin-3 is upregulated in frontotemporal dementia patients with subtype specificity', *Alzheimers Dement*, 20: 1515–26.

Bu, X. L., P. Y. Sun, D. Y. Fan, J. Wang, H. L. Sun, Y. Cheng, G. H. Zeng, D. W. Chen, H. Y. Li, X. Yi, Y. Y. Shen, L. A. Miles, P. Maruff, B. J. Gu, C. J. Fowler, C. L. Masters, and Y. J. Wang. 2022. 'Associations of plasma soluble CD22 levels with brain amyloid burden and cognitive decline in Alzheimer's disease', *Sci Adv*, 8: eabm5667.

Carvalho, D. Z., S. V. Schönwald, A. F. Schumacher-Schuh, C. W. Braga, D. O. Souza, J. P. Oses, K. C. Donis, and C. R. Rieder. 2015. 'Overnight S100B in Parkinson’s Disease: A glimpse into sleep-related neuroinflammation', *Neuroscience Letters*, 608: 57–63.

Comi, C., M. Carecchio, A. Chiocchetti, S. Nicola, D. Galimberti, C. Fenoglio, G. Cappellano, F. Monaco, E. Scarpini, and U. Dianzani. 2010. 'Osteopontin is increased in the cerebrospinal fluid of patients with Alzheimer's disease and its levels correlate with cognitive decline', *J Alzheimers Dis*, 19: 1143–8.

Crispoltoni, L., A. M. Stabile, A. Pistilli, M. Venturelli, G. Cerulli, C. Fonte, N. Smania, F. Schena, and M. Rende. 2017. 'Changes in Plasma beta-NGF and Its Receptors Expression on Peripheral Blood Monocytes During Alzheimer's Disease Progression', *JOURNAL OF ALZHEIMERS DISEASE*, 55: 1005–17.

Eruysal, E., L. Ravdin, H. Kamel, C. Iadecola, and M. Ishii. 2019. 'Plasma lipocalin-2 levels in the preclinical stage of Alzheimer's disease', *Alzheimers Dement (Amst)*, 11: 646–53.

Festoff, B. W., R. K. Sajja, P. van Dreden, and L. Cucullo. 2016. 'HMGB1 and thrombin mediate the blood-brain barrier dysfunction acting as biomarkers of neuroinflammation and progression to neurodegeneration in Alzheimer's disease', *JOURNAL OF NEUROINFLAMMATION*, 13: 12.

Gao, M., C. Zhang, L. Gao, S. Sun, L. Song, and S. Liu. 2023. 'Association between C-reactive protein-albumin ratio and overall survival in Parkinson's disease using publicly available data: A retrospective cohort study', *Heliyon*, 9: e12671.

Goldknopf, I. L., E. A. Sheta, J. Bryson, B. Folsom, C. Wilson, J. Duty, A. A. Yen, and S. H. Appel. 2006. 'Complement C3c and related protein biomarkers in amyotrophic lateral sclerosis and Parkinson's disease', *Biochem Biophys Res Commun*, 342: 1034–9.

Helmfors, L., A. Boman, L. Civitelli, S. Nath, L. Sandin, C. Janefjord, H. McCann, H. Zetterberg, K. Blennow, G. Halliday, A. C. Brorsson, and K. Kagedal. 2015. 'Protective properties of lysozyme on beta-amyloid pathology: implications for Alzheimer disease', *Neurobiology of Disease*, 83: 122–33.

Horvath, I., X. Jia, P. Johansson, C. Wang, R. Moskalenko, A. Steinau, L. Forsgren, T. Wagberg, J. Svensson, H. Zetterberg, and L. A. Morozova-Roche. 2016. 'Pro-inflammatory S100A9 Protein as a Robust Biomarker Differentiating Early Stages of Cognitive Impairment in Alzheimer's Disease', *ACS Chem Neurosci*, 7: 34–9.

Lattanzi, R., D. Maftei, C. Petrella, M. Pieri, G. Sancesario, T. Schirinzi, S. Bernardini, C. Barbato, M. Ralli, A. Greco, R. Possenti, G. Sancesario, and C. Severini. 2019. 'Involvement of the Chemokine Prokineticin-2 (PROK2) in Alzheimer's Disease: From Animal Models to the Human Pathology', *Cells*, 8.

Liang, N., K. Nho, J. W. Newman, M. Arnold, K. Huynh, P. J. Meikle, K. Borkowski, R. Kaddurah-Daouk, and Consortium Alzheimer's Disease Metabolomics. 2024. 'Peripheral inflammation is associated with brain atrophy and cognitive decline linked to mild cognitive impairment and Alzheimer's disease', *Sci Rep*, 14: 17423.

Ljubenkov, Peter A., Zachary Miller, Paige Mumford, Jane Zhang, Isabel Elaine Allen, Laura Mitic, Adam Staffaroni, Hilary Heuer, Julio C. Rojas, Yann Cobigo, Anna Karydas, Rodney Pearlman, Bruce Miller, Joel H. Kramer, Michael S. McGrath, Howard J. Rosen, and Adam L. Boxer. 2019. 'Peripheral Innate Immune Activation Correlates With Disease Severity in GRN Haploinsufficiency', *Frontiers in Neurology*, Volume 10 - 2019.

Lunetta, C., A. Lizio, F. Gerardi, C. Tarlarini, M. Filippi, N. Riva, L. Tremolizzo, S. Diamanti, C. C. Dellanoce, L. Mosca, V. A. Sansone, and J. Campolo. 2020. 'Urinary neopterin, a new marker of the neuroinflammatory status in amyotrophic lateral sclerosis', *J Neurol*, 267: 3609–16.

Masuda, T., J. Itoh, T. Koide, Y. Tomidokoro, Y. Takei, K. Ishii, and A. Tamaoka. 2017. 'Transforming growth factor-β1 in the cerebrospinal fluid of patients with distinct neurodegenerative diseases', *J Clin Neurosci*, 35: 47–49.

Naude, P. J. W., Ihgb Ramakers, W. M. van der Flier, L. C. Jiskoot, F. E. Reesink, Jahr Claassen, H. L. Koek, U. L. M. Eisel, and P. P. De Deyn. 2021. 'Serum and cerebrospinal fluid Neutrophil gelatinase-associated lipocalin (NGAL) levels as biomarkers for the conversion from mild cognitive impairment to Alzheimer's disease dementia', *Neurobiol Aging*, 107: 1–10.

Niemela, V., J. Burman, K. Blennow, H. Zetterberg, A. Larsson, and J. Sundblom. 2018. 'Cerebrospinal fluid sCD27 levels indicate active T cell-mediated inflammation in premanifest Huntington's disease', *PLoS One*, 13: 11.

Petrozziello, T., A. N. Mills, S. M. K. Farhan, K. A. Mueller, E. J. Granucci, K. E. Glajch, J. Chan, S. Chew, J. D. Berry, and G. Sadri-Vakili. 2020. 'Lipocalin-2 is increased in amyotrophic lateral sclerosis', *Muscle Nerve*, 62: 272–83.

Rocha, N. P., A. L. Teixeira, P. L. Scalzo, I. G. Barbosa, M. S. de Sousa, I. B. Morato, E. L. Vieira, P. P. Christo, A. Palotás, and H. J. Reis. 2014. 'Plasma levels of soluble tumor necrosis factor receptors are associated with cognitive performance in Parkinson's disease', *Mov Disord*, 29: 527–31.

Rydbirk, R., O. Ostergaard, J. Folke, C. Hempel, B. DellaValle, T. L. Andresen, A. Lokkegaard, A. M. Hejl, M. Bode, M. Blaabjerg, M. Moller, E. H. Danielsen, L. Salvesen, C. C. Starhof, S. Bech, K. Winge, J. Rungby, B. Pakkenberg, T. Brudek, J. V. Olsen, and S. Aznar. 2022. 'Brain proteome profiling implicates the complement and coagulation cascade in multiple system atrophy brain pathology', *Cell Mol Life Sci*, 79: 336.

Sun, Q., H. Hampel, K. Blennow, S. Lista, A. Levey, B. Tang, R. Li, and Y. Shen. 2014. 'Increased plasma TACE activity in subjects with mild cognitive impairment and patients with Alzheimer's disease', *J Alzheimers Dis*, 41: 877–86.
Trolese, Maria Chiara, Alessandro Mariani, Mineko Terao, Massimiliano de Paola, Paola Fabbrizio, Francesca Sironi, Mami Kurosaki, Silvia Bonanno, Stefania Marcuzzo, Pia Bernasconi, Francesca Trojsi, Eleonora Aronica, Caterina Bendotti, and Giovanni Nardo. 2020. 'CXCL13/CXCR5 signalling is pivotal to preserve motor neurons in amyotrophic lateral sclerosis', *EBioMedicine*, 62: 103097.
Watt, A. D., K. A. Perez, C. S. Ang, P. O'Donnell, A. Rembach, K. K. Pertile, R. L. Rumble, B. O. Trounson, C. J. Fowler, N. G. Faux, C. L. Masters, V. L. Villemagne, and K. J. Barnham. 2015. 'Peripheral alpha-defensins 1 and 2 are elevated in Alzheimer's disease', *J Alzheimers Dis*, 44: 1131–43.
Wormser, U., J. Mandrioli, M. Vinceti, N. Fini, A. Sintov, B. Brodsky, E. Proskura, and Y. Finkelstein. 2016. 'Reduced levels of alpha-1-antitrypsin in cerebrospinal fluid of amyotrophic lateral sclerosis patients: a novel approach for a potential treatment', *J Neuroinflammation*, 13: 131.
Xia, J. Q., Y. F. Cheng, S. R. Zhang, Y. Z. Ma, J. J. Fu, T. M. Yang, L. Y. Zhang, J. M. Burgunder, and H. F. Shang. 2024. 'The characteristic and prognostic role of blood inflammatory markers in patients with Huntington's disease from China', *Front Neurol*, 15: 1374365.
Xiong, M., Q. Qian, X. Liang, and Y. D. Wei. 2022. 'Serum levels of lipocalin-2 in patients with Parkinson's disease', *Neurol Sci*, 43: 1755–59.
Zheng, Huimin, Tai Wang, Changhe Shi, Liyuan Fan, Yun Su, Yu Fan, Xinwei Li, Jing Yang, Chengyuan Mao, and Yuming Xu. 2022. 'Increased PRR14 and VCAM-1 level in serum of patients with Parkinson's disease', *Frontiers in Neurology*, Volume 13 - 2022.

```
# ========================
# 1. DATA cleaning
# ========================

df <- significantie |>
  janitor::clean_names() |>
  pivot_longer(
    cols = -c(biomarkers, number_of_studies,
neurodegenerative_diseases),
    names_to = c("measure", "type_info"),
    values_to = "value",
    names_pattern = "^(.*)_([^_]+)$"
  ) |>
  pivot_wider(
    names_from = type_info,
    values_from = value
  ) |>
  mutate(across(where(is.character), as.factor))

df$neurodegenerative_diseases <- factor(
  df$neurodegenerative_diseases,
  levels = c("AD","PD","FTD","ALS","DLB","MSA","HD","PSP")
)

df$biomarkers <- factor(
  df$biomarkers,
  levels = c("IL-6","TNF-a","IL-1b","CRP","hs-
CRP","IL-10","MCP-1","YKL-40","NLR")
)

# -------------------------
# Colours and shapes
# -------------------------
biomarker_colors <- c(
  "IL-6"   = "#CC79A7",
  "TNF-a"  = "#D55E00",
  "IL-1b"  = "#000000",
  "CRP"    = "#E69F00",
  "hs-CRP" = "#000000",
  "IL-10"  = "#4D4D4D",
  "MCP-1"  = "#0072B2",
  "YKL-40" = "#009E73",
  "NLR"    = "#0072B2"
)

biomarker_shapes <- c(
  "IL-6"   = 22,
  "TNF-a"  = 24,
  "IL-1b"  = 0,
  "CRP"    = 21,
  "hs-CRP" = 1,
  "IL-10"  = 2,
  "MCP-1"  = 5,   # open diamond
  "YKL-40" = 23,
```

```
  "NLR"    = 24
)

biomarker_labels <- c(
  "IL-6"   = "IL-6",
  "TNF-a"  = "TNF-α",
  "IL-1b"  = "IL-1β",
  "CRP"    = "CRP",
  "hs-CRP" = "hs-CRP",
  "IL-10"  = "IL-10",
  "MCP-1"  = "MCP-1",
  "YKL-40" = "YKL-40",
  "NLR"    = "NLR"
)

theme_set(theme_bw())

# --------------------------
# The plot
# --------------------------

p <- df |>
  filter(!is.na(significance), !is.na(consistency)) |>
  ggplot(aes(
    x = significance,
    y = consistency,
    size  = number_of_studies,
    color = biomarkers,
    shape = biomarkers,
    fill  = biomarkers
  )) +

  geom_point(
    position = position_jitter(width = 2, height = 2),
    alpha  = 0.85,
    stroke = 1
  ) +

  # X-Axis
  scale_x_continuous(
    limits = c(-12, 112),
    breaks = c(0, 50, 100),
    labels = c("n.s. > s.", "s. = n.s.", "s. > n.s."),
    expand = expansion(mult = 0.05)
  ) +

  # Y-Axis
  scale_y_continuous(
    limits = c(-120, 120),
    breaks = c(-100, 0, 100),
    labels = c("↑", "–", "↓"),
    expand = expansion(mult = 0.06)
  ) +
```

```
  scale_color_manual(values = biomarker_colors, labels =
biomarker_labels) +
  scale_fill_manual(values = biomarker_colors, labels =
biomarker_labels) +
  scale_shape_manual(values = biomarker_shapes, labels =
biomarker_labels) +

  facet_wrap(~ neurodegenerative_diseases, ncol = 2) +

  labs(
    title = "Diagnostic Differentiation by Biomarkers Across
Neurodegenerative Diseases",
    x = "Significance of results",
    y = "Consistency of direction",
    color = "Biomarkers",
    shape = "Biomarkers",
    fill  = "Biomarkers",
    size  = "Number of Studies"
  ) +

  theme(
    legend.position = "right",
    plot.title = element_text(hjust = 0.5, size = 16, face =
"bold"),
    axis.text.y  = element_text(size = 14),
    axis.ticks.y = element_blank(),
    panel.grid.major = element_blank(),
    panel.grid.minor = element_blank(),
    panel.border = element_rect(colour = "black", fill = NA, size =
0.5),
    strip.text = element_text(size = 12)
  )

p
```
